# Reaction Mechanism, Challenges, and Strategies of High‐Energy‐Density Sodium‐Ion Batteries

**DOI:** 10.1002/advs.202519427

**Published:** 2026-01-14

**Authors:** Dan Yu, Yuxin Cui, Qinghao Chen, Lu Gao, Xia Liu, Tianci Li, Wenju Zhu, Quanxiang Li, Weimin Kang

**Affiliations:** ^1^ State Key Laboratory of Advanced Separation Membrane Materials School of Textile Science and Engineering Tiangong University Tianjin 300387 P. R. China; ^2^ School of Materials Science and Engineering Tianjin University Tianjin 300074 P. R. China; ^3^ Institute for Frontier Materials Deakin University Waurn Ponds Campus Locked Bag 20000 Geelong Victoria 3220 Australia; ^4^ School of Chemical Engineering and Technology Tiangong University Tianjin 300387 P. R. China

**Keywords:** conversion‐type cathode materials, reaction mechanisms, sodium‐ion batteries, specific capacity, volume expansion

## Abstract

Sodium‐ion batteries (SIBs) have attracted considerable research interest for large‐scale energy storage due to the natural abundance and wide geographic distribution of sodium. Among various cathode materials, conversion‐type cathodes have garnered particular attention due to their element richness, high specific capacity, and enhanced safety and reliability. However, their practical application faces challenges, including huge volume expansion, incomplete reversible conversion reactions, and severe side reactions. This review summarizes the unique advantages and critical issues of conversion‐type cathode materials, along with recent advances in various cathodes for SIBs. First, the reaction mechanisms of different conversion‐type cathode materials are analyzed and summarized to provide theoretical foundations for practical implementation. Particularly, we propose novel countermeasures addressing common cathode challenges, offering new perspectives for future research on these materials. Notably, composite conversion‐type cathodes demonstrate substantial potential as evidenced by their remarkable energy density. Future research should focus on in‐depth investigations of reaction mechanisms, modification strategies, and characterization techniques for conversion‐type cathodes, thereby advancing the development of high‐energy‐density cathode materials.

## Graphical abstract

Focusing on high‐energy‐density sodium‐ion batteries, this review highlights the advantages offered by conversion‐type cathode materials. The currently studied cathode materials are systematically introduced. By analyzing sulfur, oxygen, and transition metal halides and other conversion‐type cathodes, the key challenges associated with these cathodes are identified, and common strategies are proposed. The promising development prospects of conversion‐type cathode materials are emphasized.

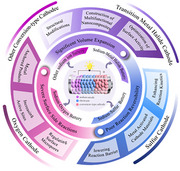

## 1 Introduction

**Figure 1:**
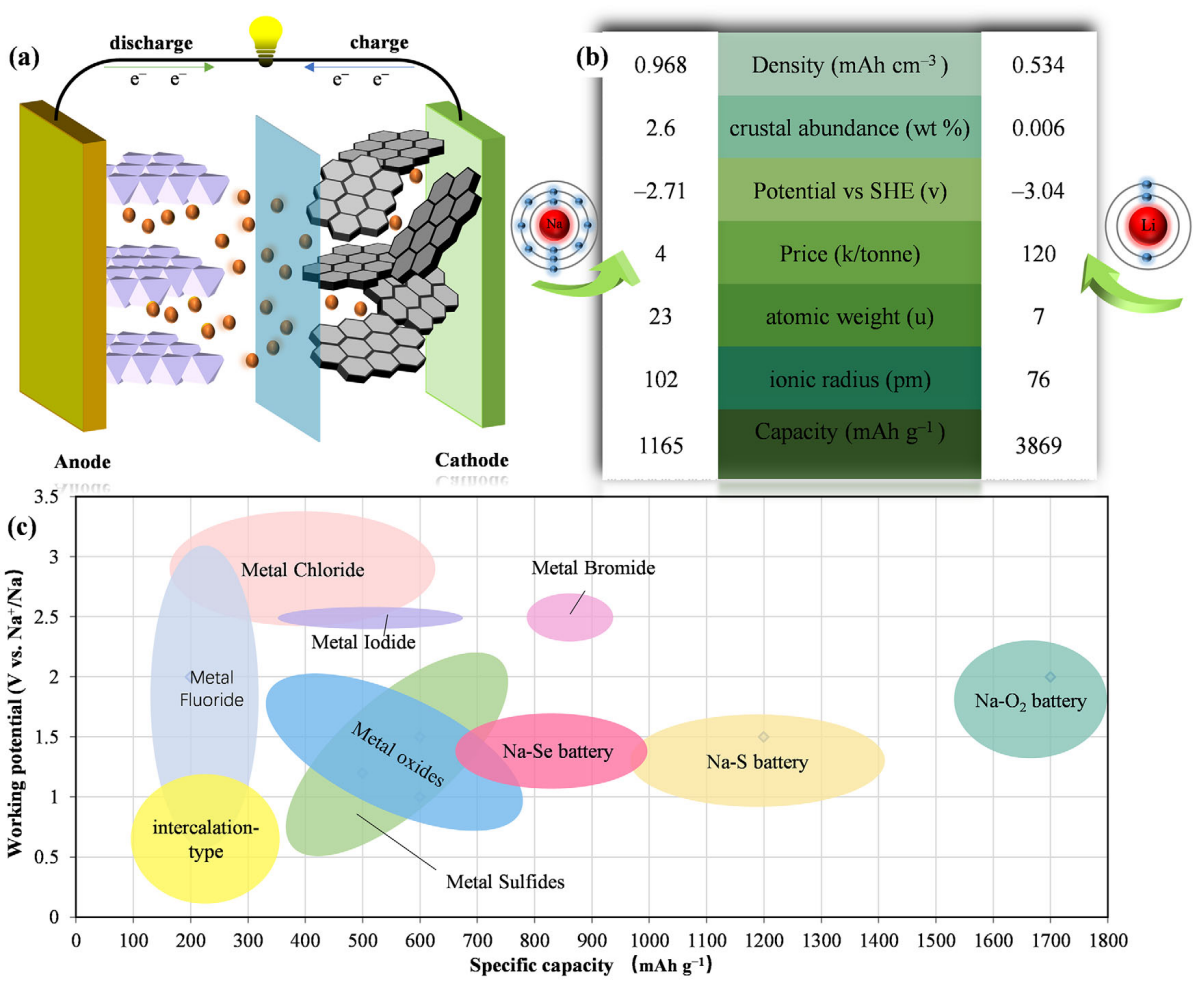
a) Schematic diagram illustrating the working mechanism of a sodium‐ion battery. b) Comprehensive comparison of sodium and lithium elements. c) Voltage capacity comparison of typical sodium‐ion battery conversion‐type and intercalation‐type cathode materials.

Driven by low‐carbon initiatives, the rapid growth of new energy vehicles has intensified competition in automotive batteries.^[^ ^1^ ^]^ Despite the dominance of lithium‐ion batteries (LIBs) in the energy field, lithium's scarcity (0.006 wt.% crustal abundance) constrains large‐scale applications while presenting challenges in safety and environmental impact.^[^ ^2^ ^]^ Sodium‐ion batteries (SIBs) emerge as a promising alternative for grid‐scale storage due to sodium's abundance (2.6 wt.%), unlimited seawater reserves, and widespread.^[^ ^3^, ^4^ ^]^ Although sharing electrochemical principles with LIBs (**Figure** **1**a), Na^+^ ions move back and forth between the cathode and anode, SIBs still face the problem of kinetic limitations.^[^ ^5^ ^]^ As depicted in Figure 1b, sodium's larger ionic radius (102 vs Li⁺’s 76 pm) and higher atomic mass (23 vs 7 u)^[^ ^6^ ^]^ cause slower ion diffusion, volume expansion issues, and reduced cyclability.^[^ ^7^, ^8^ ^]^ Despite these challenges, raw material availability and wide distribution of SIBs position them as a viable alternative to lithium‐ion batteries in the future.^[^ ^9^, ^10^ ^]^

Cathode selection critically determines SIB performance.^[^ ^11^ ^]^ It stores and provides energy, as well as holds the electrons and ions transferred from the anode during the discharge process, preparing them for recharging. For sodium‐ion batteries, highly stable cathodes are required to mitigate volume changes and extend cycle life.^[^ ^12^ ^]^ Moreover, using a cathode with high specific capacity, in conjunction with a sodium metal anode, can effectively enhance the battery's specific capacity. Currently, cathode materials are generally classified into two types based on their electrochemical reaction mechanisms: intercalation‐type and conversion‐type.^[^ ^13^ ^]^ Intercalation‐type (layered transition metal oxides,^[^ ^14^ ^]^ polyanionic compounds,^[^ ^15^ ^]^ Prussian blue/white analogues compound,^[^ ^16^ ^]^ and organic compounds^[^ ^17^ ^]^), offering structural stability but limited capacity due to single‐electron redox reactions and Na⁺ insertion constraints, which induce crystal distortion and conductivity issues. Conversion‐type (S, O_2_, Se, transition metal compounds), utilizing multi‐electron redox processes to achieve higher energy densities (Figure 1c).^[^ ^18^ ^]^ while accommodating large Na⁺ radii, though challenged by volume expansion, structural degradation, and pulverization during cycling.^[^ ^19^, ^20^ ^]^ Each type of conversion‐type cathode material offers unique advantages and research value.^[^ ^21^ ^]^ Sulfur, for instance, is advantageous due to its low toxicity, high abundance, and high specific capacity, and its commercialization is progressing from grid energy storage devices to new energy vehicles, making it a highly promising material for high‐energy‐density SIBs.^[^ ^22^ ^]^ Selenium, similarly, has emerged as an attractive cathode material in recent years due to its high ionic conductivity and competitive theoretical specific capacity.^[^ ^23^ ^]^ Oxygen cathodes offer sustainability, safety, reliability, and high energy density, holding promise for the development of safe, environmentally friendly, and efficient batteries.^[^ ^24^ ^]^ Transition metal compounds are also noteworthy for their wide variety, low price, and environmental friendliness. CuCl_2_, in particular, has shown reversible specific capacity close to the theoretical value, making it a promising candidate for high‐energy‐density batteries.^[^ ^25^ ^]^ Despite their advantages, conversion‐type cathodes face intrinsic challenges during sodium‐ion battery operation: crystal growth, volume expansion, structural damage, and electrode pulverization collectively induce rapid capacity decay.^[^ ^26^ ^]^ Addressing these problems through fundamental research is critical to harnessing the full potential of these materials.

**Figure 2:**
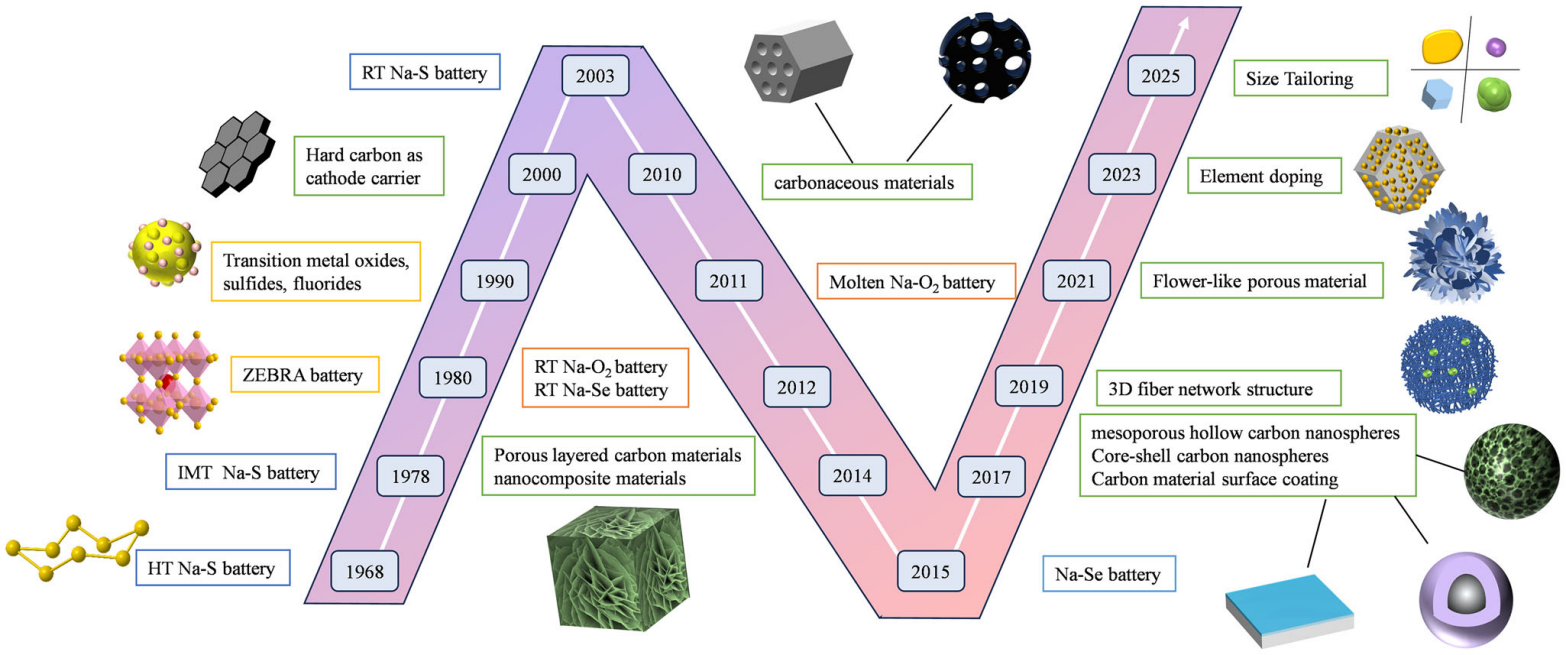
Schematic diagram illustrating the development of conversion‐type cathodes in sodium‐ion batteries.

Currently, growing research interest in conversion‐type cathode materials has spurred a proliferation of pioneering studies, elucidating their common advantages and challenges while underscoring the critical need for comprehensive reviews.^[^ ^27^ ^]^ **Figure** **2** illustrates the development of sodium‐ion batteries featuring conversion‐type cathodes and the trends in cathode structures studied in recent years. Among these, sulfur, oxygen, transition metal chlorides, and selenium have been the primary focus for sodium‐ion batteries. These materials enable high‐energy‐density batteries, including sodium–sulfur (Na–S),^[^ ^28^ ^]^ sodium–oxygen (Na–O_2_),^[^ ^29^ ^]^ sodium–selenium (Na–Se),^[^ ^30^ ^]^ and sodium‐metal chloride (ZEBRA) batteries.^[^ ^31^ ^]^ Each type of SIBs has distinct advantages and applications. Despite extensive research on developing and modifying conversion‐type cathodes, systematic comparative analyses of their reaction mechanisms remain scarce. Neither the stepwise reaction pathways within battery systems nor the fundamental similarities/differences in electrochemical processes across cathode types have been clearly established. Consequently, systematic summarization of reaction mechanisms in SIBs is essential to elucidate synergistic relationships between conversion‐type cathodes and other battery components, thereby enabling optimal utilization of these cathode materials.^[^ ^32^ ^]^ Furthermore, while offering significant benefits, conversion‐type cathode materials face common issues, including huge volume expansion, incomplete reversible conversion reactions, and side reactions.^[^ ^33^ ^]^ In severe cases, these issues may lead to battery failure and safety hazards.^[^ ^34^ ^]^ Therefore, it is crucial to summarize the common challenges faced by conversion‐type cathode materials and to compile corresponding solutions to address these problems.

In this review, we emphasize the distinctive advantages of conversion‐type cathode materials: wide distribution, high safety, and high energy density. A concise overview of key benefits and development trends for these cathodes in sodium‐ion batteries is presented. Subsequently, we conduct focused analyses of reaction mechanisms across major cathode systems, including sulfur (S), oxygen (O_2_), transition metal halides, and related materials, while systematically examining commonalities and divergences in reaction pathways among different batteries. Specifically, S cathodes undergo multistep conversion reactions, with Se cathodes exhibiting analogous processes. Other conversion‐type cathodes demonstrate simplified reaction pathways, typically completing within single or limited steps. Notably, all variants adhere to a unified conversion reaction equation. Furthermore, we also pay attention to the common challenges of conversion cathode, including huge volume expansion, incomplete reversible reactions, and severe side reactions. Concurrently, we compile targeted countermeasures from cutting‐edge research to provide strategic insights for practical implementation. Volume expansion is mitigated through structural porosity engineering, nano‐composite fabrication, and surface activity optimization. Limited specific capacity is enhanced via electrocatalytic additive, strategic metal doping, and 3D conductive network construction. Parasitic reactions are suppressed by metal‐polarity‐driven product anchoring, Catalytic surface modification, and Pore‐confinement strategies. Collectively, these approaches synergistically improve electrode integrity, energy density, and reaction selectivity in sodium‐ion batteries. At the end of the article, combined with the latest research in the field of conversion‐type cathode materials, future directions for the development of conversion‐type cathodes are envisioned. The review aims to provide insights into future research and potential applications of conversion‐type cathode materials in SIBs.

## 2 Conversion‐Type Cathode Materials

**Figure 3:**
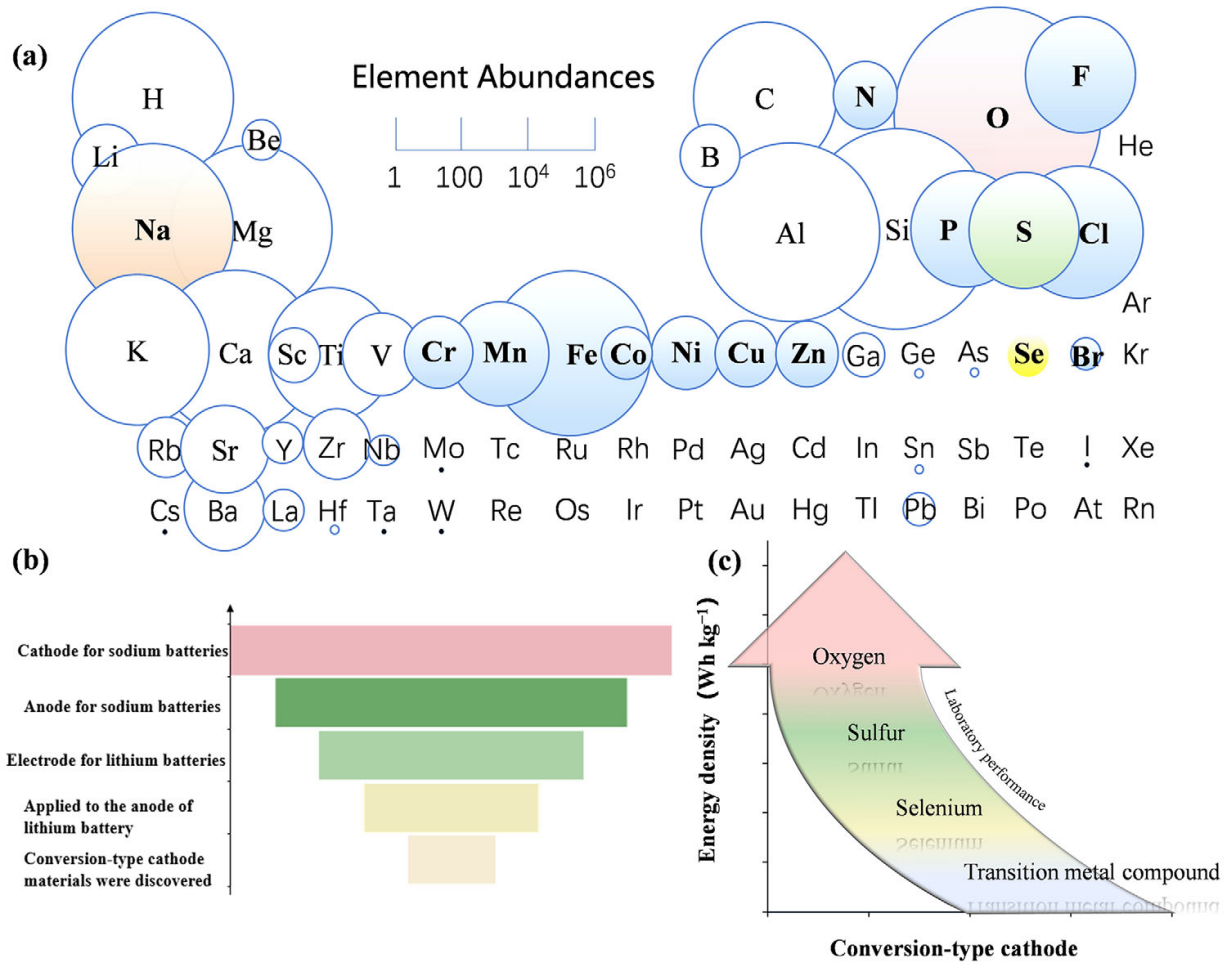
a) A periodic table illustrating the abundance of relevant elements in the Earth's crust. Reproduced with permission.^[^ ^36^ ^]^ Copyright 2018, John Wiley and Sons. b) Origin of the development of the conversion‐type cathode for sodium‐ion batteries. c) Theoretical specific capacity of various conversion‐type cathodes.

Conversion‐type cathode materials have garnered significant research interest for sodium‐ion batteries due to their high specific capacity, natural abundance, and wide distribution. These materials, which include sulfur (S), selenium (Se), oxygen (O), transition metal oxides (TMOs), transition metal sulfides (TMSs), and transition metal halides (TMHs), operate on a new conversion reaction mechanism for storing Na⁺ ions.^[^ ^35^ ^]^ **Figure** **3**a illustrates the abundance of these elements.^[^ ^36^ ^]^ For instance, S and O are among the most abundant elements on Earth.^[^ ^37^ ^]^ Similarly, transition metals, including nickel (Ni), iron (Fe), copper (Cu), and zinc (Zn), are widely available.^[^ ^38^ ^]^ From a safety perspective, many of these elements (e.g., S, O, N, P) are inherently stable and environmentally benign.^[^ ^39^ ^]^ Although halogens can be hazardous on their own, they are more stable and environmentally friendly when used in the form of salts in cathodes.^[^ ^40^ ^]^ Thus, conversion‐type cathode materials hold significant advantages in terms of distribution range, resource abundance, and safety, which will accelerate the development of high‐energy‐density cathode materials.

Among these materials, S cathodes stand out due to their remarkable theoretical specific capacity of 1672 mAh g^−1^ and a volumetric capacity of 3467 mAh cm^−3^, the highest among conversion‐type cathodes.^[^ ^41^ ^]^ The development of sodium–sulfur (Na–S) batteries has progressed from high‐temperature (HT, >300 °C) and intermediate‐temperature (MT) systems to more practical room‐temperature (RT) Na–S batteries.^[^ ^42^ ^]^ This evolution has mitigated severe safety issues associated with molten sodium and sulfur, such as violent reactions and the need for corrosion‐resistant materials.^[^ ^43^ ^]^ At the same time, RT Na–S has a higher theoretical energy density(1274 Wh kg^−1^). These improvements enable broader applications, including stationary grids and electric vehicles.^[^ ^44^ ^]^

Se cathodes have rapidly developed as an attractive alternative to sulfur. Se cathodes exhibit higher ionic conductivity (10^−3^ S m^−1^), which contributes to better rate performance and faster reaction kinetics.^[^ ^45^, ^46^ ^]^ Although its theoretical specific capacity (675 mAh g^−1^) is lower than S's, Se offers a competitive theoretical volume capacity of 3253 mAh cm^−3^.^[^ ^47^ ^]^ Research on Na–Se batteries began relatively late, around 2012, and has since revealed challenges similar to sulfur, including shuttle effects, slow kinetics, and large volume changes.^[^ ^48^ ^]^ So far, the Na–Se batteries are still in their early stages, but remarkable progress has been made.

O cathodes, utilized in sodium–oxygen (Na–O_2_) batteries, offer high theoretical specific and volume capacities (up to 1670 mAh g^−1^ and 2699 mAh cm^−3^, respectively), comparable to S.^[^ ^49^ ^]^ Early Na–O_2_ batteries operated at elevated temperatures, influenced by HT Na–S systems, but research has since shifted to room‐temperature systems.^[^ ^50^ ^]^ However, a major scientific challenge lies in the controversial nature of the discharge products, with ongoing debate between sodium superoxide (NaO_2_) and sodium peroxide (Na_2_O_2_) as the primary product, a situation less defined than in lithium–oxygen (Li–O_2_) and potassium–oxygen (K–O_2_) batteries.^[^ ^51^ ^]^ This ambiguity, coupled with high charging overpotentials, poor cycling performance, and various side reactions influenced by temperature, voltage, and catalysts, presents unique technological challenges for this gas–solid conversion reaction system.^[^ ^52^ ^]^

TMH cathodes are promising due to their wide variety, high energy density, and potential for long cycle life. Their theoretical specific capacities span a wide range, typically from 500 to 1500 mAh g^−1^.^[^ ^53^ ^]^ The development of sodium metal halide batteries, notably the ZEBRA battery introduced in the 1980s, which used NiCl_2_ and demonstrated a practical specific energy of 130 Wh kg^−1^.^[^ ^54^ ^]^ Compared to other systems, the sodium metal halide battery has been shown to offer greater safety and commercial viability.^[^ ^55^ ^]^ Among them, fluorides(F) are attractive due to high electronegativity, and chlorides(Cl) have been widely studied; bromides(Br) and iodides(I) remain less explored.^[^ ^56^, ^57^ ^]^ However, reaction mechanisms, especially for metal fluorides, are not yet fully understood, requiring further fundamental investigation.^[^ ^58^ ^]^

TMO cathodes, based on Fe, Cu, Mn, and Co, combine the advantages of transition metal compounds and O cathodes. They are known for good stability, simple preparation, environmental friendliness, and high specific capacities (e.g., 1007 mAh g^−1^ for Fe_2_O_3_, 926 mAh g^−1^ for Fe_3_O_4_, and 890 mAh g^−1^ for Co_3_O_4_).^[^ ^59^, ^60^ ^]^ Early research focused significantly on TMOs, but they suffer from severe volume changes during cycling, leading to active material pulverization and rapid capacity fade.^[^ ^61^ ^]^ What is worse, the conversion reaction can form various stable sodium oxides (Na_2_O, Na_2_O_2_, NaO_2_) and intermediate phases, increasing reaction irreversibility.^[^ ^62^, ^63^ ^]^ Future work needs to delve deeper into the crystallographic, kinetic, and thermodynamic evolution of their conversion reactions,^[^ ^64^ ^]^ so that better sodium ion storage performance can be obtained.

TMS cathodes, such as FeS^[^ ^65^ ^]^ and MnS,^[^ ^66^ ^]^ share the benefits of high abundance and high energy density with TMOs. They began being studied as early as the 1970s and were applied in sodium‐ion batteries after 2000.^[^ ^67^ ^]^ Compared to sodium metal oxide batteries, sodium metal sulfide batteries often exhibit a longer cycle life and smaller volume expansion. The final discharge product, Na_2_S, is generally considered more reversible than Na_2_O, and the weaker Na–S bond favors the conversion reaction, potentially leading to a higher specific capacity. Although their performance may not surpass that of S cathodes, TMSs hold considerable potential and warrant deeper analysis.

In summary, conversion‐type cathode materials offer a compelling pathway to high‐energy‐density sodium‐ion batteries, leveraging high accessibility and material sustainability. In the late 20th century, lithium‐ion battery research established the reaction mechanisms of conversion‐type anode materials. Subsequently, research has gradually shifted toward sodium‐ion batteries (Figure 3b) due to the exceptional compatibility of conversion‐type cathodes with large‐radius ions (e.g., Na⁺).^[^ ^68^ ^]^ Notably, to pursue higher energy densities, research began on sodium‐metal batteries assembled with sodium metal anodes and conversion‐type cathode materials. This field has developed rapidly over the past decade.^[^ ^69^ ^]^ However, during this research, challenges have emerged, including discrepancies between theoretical and practical specific capacities, significant volume changes, and sluggish kinetics. Therefore, a deeper investigation into these challenges is essential to leverage redox reactions for surpassing the capacity limitations of existing materials, ultimately delivering ultrahigh energy densities (Figure 3c).^[^ ^70^ ^]^

## 3 Reaction Mechanism of Conversion‐Type Cathode

Compared to intercalation‐type cathodes, conversion‐type cathode materials utilize conversion reactions to achieve higher energy density. Consequently, different reaction mechanisms will exhibit different electrochemical performances. As clearly illustrated in **Table** **1**, the essential features of conversion and intercalation mechanisms can be directly compared.^[^ ^71^ ^]^ Unlike an intercalation‐type cathode, the conversion reaction involves the breaking of chemical bonds, leading to the formation of new chemical substances.^[^ ^72^ ^]^ This process, coupled with multi‐electron transfer and the accommodation of large‐radius Na^+^, enables exceptionally high theoretical specific capacity. However, this advantage comes with trade‐offs: conversion‐type cathodes generally exhibit more substantial volume expansion, slower reaction kinetics, and shorter cycle life compared to intercalation‐type. Despite these challenges, the prominent advantages of conversion mechanisms, namely their high energy density and the elemental abundance of required materials, are particularly outstanding. These inherent strengths position them favorably to break through the current research bottleneck, paving the way for SIB with significantly enhanced energy density. Therefore, the conversion reaction mechanism will be discussed in detail below.

**Table 1:** Comparison of conversion and intercalation mechanisms.

Essential feature	Conversion mechanism	Intercalation mechanism
Reaction process	The breaking of chemical bonds and the formation of new substances.	Na^+^ insertion/extraction into/from the crystal structure
Electron transfer number	Multi‐electron redox processes	Single‐electron redox reactions
Na⁺ insertion	Accommodating large Na⁺ radii	Na⁺ insertion constraints
Theoretical specific capacity	Usually > 500 mAh g^−1^	< 200 mAh g^−1^
Volume change	Typically, 50–300%	< 10%
Reaction kinetics	Relatively slow	Relatively fast
Cycling stability	Poor Rapid capacity fading	High Thousands of cycles.
Key challenges	Volume expansion, side reactions, poor reversibility, slow kinetics	Limited capacity, Reliance on scarce elements(Co)
Inherent advantage	Relatively high theoretical energy density, elemental abundance	Exceptional cycle life

**Figure 4:**
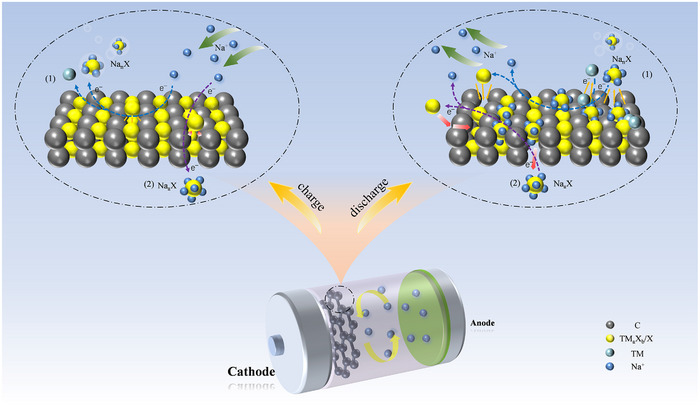
Diagram illustrating the conversion reaction process of conversion‐type cathodes in sodium batteries.

The conversion reaction process in sodium batteries parallels that of lithium battery conversion‐type cathodes, as illustrated in **Figure** **4**:^[^ ^73^ ^]^

(1) $$TM_{a}X_{b}+\left(b\;n\right)Na^{+}+\left(b\;n\right)e^{-}⇌\mathrm{aTM}+\mathrm{bN}a_{n}X$$

(2) $$X+\mathrm{aN}a^{+}+ae^{-}⇌Na_{a}X$$

Among them, Equation (1) represents the conversion formula,^[^ ^74^ ^]^ where TM stands for transition metals, such as Fe, Cu, Co, Mn, etc., and X denotes anions, such as S, N, P, F, etc. This formula is mainly applied to metal compounds with high‐valence metal ions. Equation (2) represents an extended conversion formula,^[^ ^40^, ^75^ ^]^ where X stands for S, Se, I, etc., and includes various sulfur and halogen elements, as well as O_2_. Since the cathode materials derived from this formula are free of heavy metals, they can provide a higher weight capacity than transition metal compounds, and some active products from Equation (1) are also observed in this reaction. Notably, both Equations (1) and (2) show that conversion‐type cathode materials do not contain Na^+^, necessitating the use of sodium metal as the anode in sodium batteries to further enhance the overall energy density of the battery.

From the above Equation, it is evident that the conversion reaction process is based on the bond cleavage of TM and X in TM_a_X_b_ compounds, leading to the formation of a new chemical bond between Na and X. Consequently, conversion‐type cathodes offer a higher theoretical specific capacity compared to intercalation‐type ones.^[^ ^76^ ^]^ During the initial discharge stage, the overall morphology of the TM_a_X_b_ nanoparticles remains unchanged. As reduction progresses, these nanoparticles become fully reduced, forming metallic nanoparticles dispersed within a matrix of the sodium‐containing product (Na_n_X). P. Poizot et al.^[^ ^74^ ^]^ utilized a Philips CM12 Transmission Electron Microscope (TEM) with selected area electron diffraction (SAED) mode and bright field images to observe that, upon complete reduction of CoO by lithium, 1000 CoO nanoparticles decomposed into 10–20 metal nanoparticles distributed in a lithium (Li_2_O) matrix, with Li_2_O nanoparticles surrounded by a solid electrolyte interface. Hook^[^ ^77^ ^]^ demonstrated that the resulting metallic nanoparticles are typically smaller than 5 nm, which induces significant volume changes. This process typically involves multiple stages, and often more than two products are present simultaneously in the same voltage range.^[^ ^78^ ^]^ For example, when S acts as the cathode of SIBs, a series of intermediate products, such as Na_2_S_8_, Na_2_S_6_, Na_2_S_5_, Na_2_S_4_, and other soluble polysulfides, can be produced, with the possibility of simultaneous formation.^[^ ^79^ ^]^ These intermediate products undergo conversion reactions among themselves, ultimately forming Na_2_S.^[^ ^80^ ^]^ Therefore, the multistep conversion reactions also contribute to the high specific capacity of the sulfur cathode.

To fully understand the advantages of conversion reactions, it is crucial to specify the energy density of the constituent SIBs, including the cathode potential and the amount of electricity stored per unit weight and volume of the cathode material. The theoretical specific capacity (by weight) and volume capacity, along with the theoretical potential, are key indices for evaluating the performance of conversion‐type cathode materials in sodium batteries.^[^ ^40^ ^]^ Specific capacity, as a primary index for assessing energy storage materials, directly influences the material's application potential. The maximum achievable capacity, known as the “ceiling,” is closely related to battery stability, safety, and other properties.^[^ ^81^ ^]^ However, SIBs with conversion‐type cathodes often exhibit poor electrochemical cycling stability, and the actual specific capacity usually falls short of the theoretical value.^[^ ^82^ ^]^ Additionally, since conversion‐type cathodes undergo volume changes, the volume capacity index is also important. Moreover, electric potential, an intrinsic battery attribute, reflects its ability to convert other forms of energy into electrical energy.^[^ ^83^ ^]^ A higher electric potential indicates a larger potential difference between the battery's positive and negative electrodes, resulting in greater charge migration in the circuit. This increased capacity enhances the battery's energy storage capability.^[^ ^84^ ^]^

According to the Nernst equation, the electric potential (emf), denoted as E, can be calculated from the following equation:

(3) $$\Delta_{r}G=n\Delta G_{f}\left(Na_{n}X\right)-\Delta G_{f}\left(TM_{a}X_{b}\right)=-\mathrm{nFE}$$

(4) $$E=\frac{\Delta_{r}G}{-\mathrm{nf}}=\frac{n\Delta G_{f}\left(Na_{n}X\right)-\Delta G_{f}\left(TM_{a}X_{b}\right)}{-\mathrm{nF}}$$

where Δ_*r*_ *G* is the Gibbs free energy change for the reaction (kJ mol^−1^), Δ*G_f_* is the Gibbs free energy change for the formation of the compound (kJ mol^−1^), and the standard thermodynamic data for Δ*G_f_* can be obtained from first‐principles calculations.^[^ ^85^ ^]^ F represents Faraday's constant (96 485 C mol^−1^), and n is the number of electrons involved in the reaction (mol).

The specific capacity C (mAh g^−1^) of the *TM_a_X_b_* cathode material can be calculated from Equation (5):

(5) $$C=\frac{\mathrm{nF}}{3.6M}$$

where M is the sum of the relative molecular weights of the positive and negative materials of the battery, and 3.6 in the calculation formula is for the convenience of converting the unit, using 3.6 C = 1 mAh to convert C mol^−1^ to mAh g^−1^.

In addition to this, according to the above calculations, the energy density and specific energy can also be derived, which is calculated by Equations (6) and (7):

(6) $$E_{M}=E\cdot C=\frac{\Delta_{r}\mathrm{GC}}{-\mathrm{nF}}=\frac{\left[n\Delta G_{f}\left(Na_{n}X\right)-\Delta G_{f}\left(TM_{a}X_{b}\right)\right]C}{-\mathrm{nF}}$$

(7) $$E_{V}=E_{M}\;\rho$$

where ρ in the above equation denotes the theoretical density (g cm^−3^).

**Figure 5:**
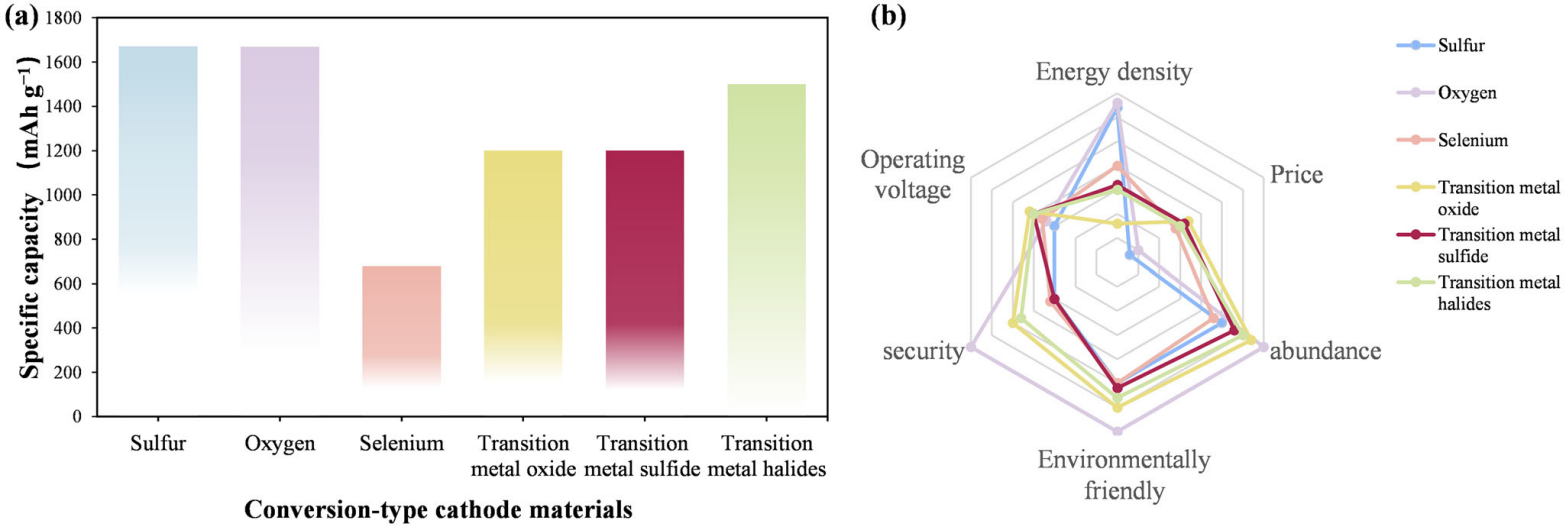
Comparison of a) specific capacity and b) performance across various conversion‐type cathodes.

In summary, conversion‐type cathode materials exhibit significant potential for achieving high‐energy‐density batteries, particularly due to the ultrahigh specific capacities offered by sulfur and oxygen cathodes. Although transition metal compounds typically deliver lower capacities, transition metal halides have demonstrated promising performance. To date, metal halide cathodes have achieved reversible specific capacities approaching their theoretical limits, positioning them as strong candidates for maximizing energy storage. Furthermore, fluorides exhibit higher redox potentials than other compounds. This elevated potential facilitates the formation and decomposition of reaction products, thereby enhancing reaction reversibility and ultimately improving battery‐specific capacity. Additionally, the ionic bonds formed between fluorine and metals—characterized by high electronegativity and lattice energy—contribute to product stability and reduce side reactions. Other conversion‐type cathode materials, including selenium cathodes, metal sulfides (TMSs), and metal oxides (TMOs), exhibit comparable performance to the aforementioned materials. **Figure** **5**a,b provides a comparison and analysis of the specific capacities and properties of various conversion‐type cathodes. Notably, sulfur, oxygen, and transition metal halides (TMHs) have attracted significant attention due to their respective unique advantages and are expected to drive sodium batteries toward higher energy densities in the future. Therefore, investigating the specific reaction mechanisms and clarifying performance distinctions among conversion‐type cathode forms is the cornerstone for advancing next‐generation cathode materials. Following this, the reaction mechanism of S, O, and TMHs and other conversion‐type cathodes (Se, TMOs, TMSs) in sodium‐ion batteries will be examined in detail.

### 3.1 Reaction Mechanism of Sulfur Cathodes

**Figure 6:**
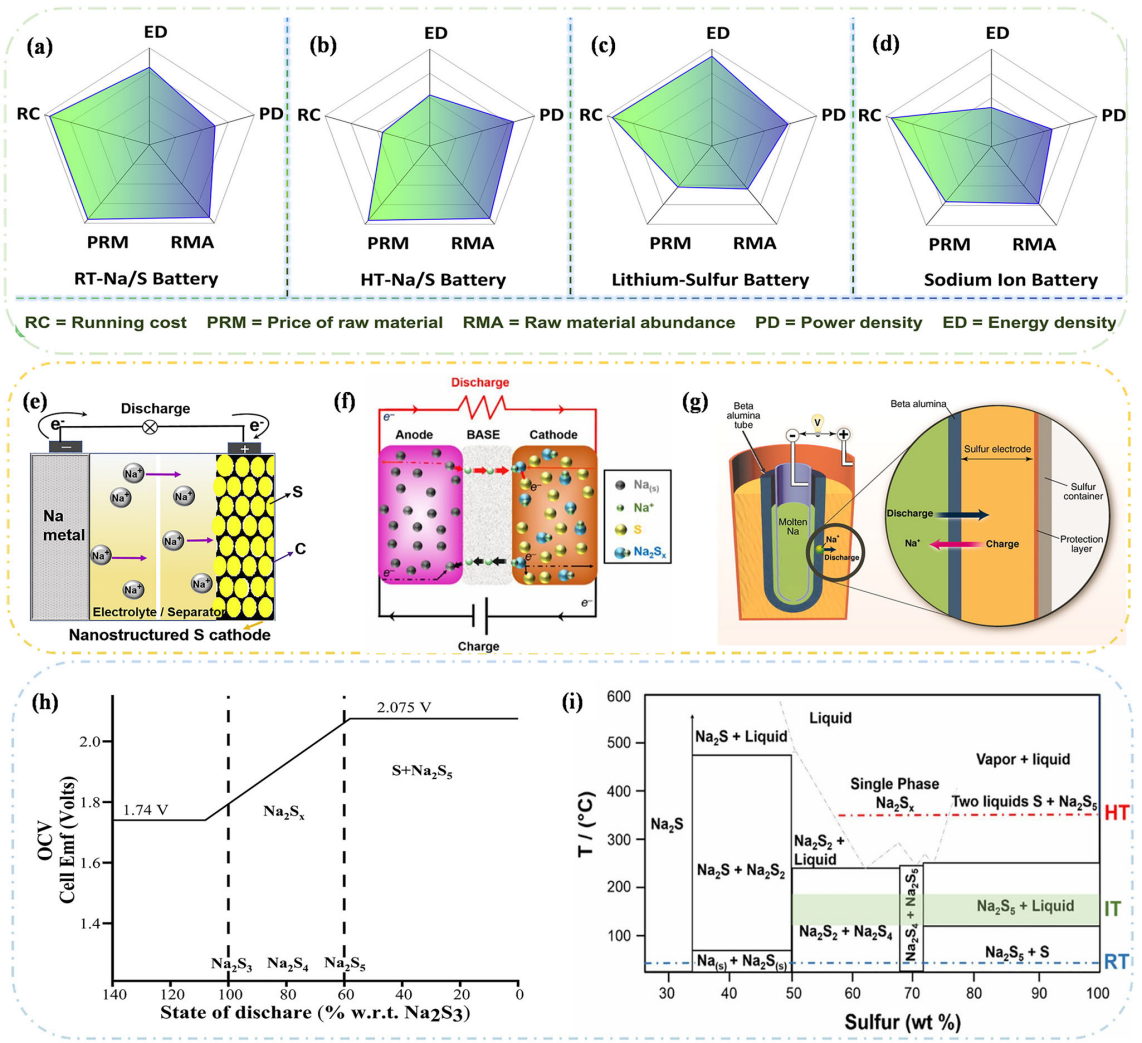
Multi‐scale experimental characterizations for ion‐transport and corresponding structures in batteries. Performance comparison of various sodium battery systems, including: a) room temperature Na–S battery, b) high temperature Na–S battery, c) Li–S battery, and d) sodium‐ion battery. Reproduced with permission.^[^ ^86^ ^]^ Copyright 2021, Elsevier. e) Structure and working principle of a room temperature Na–S battery. Reproduced with permission.^[^ ^43^ ^]^ Copyright 2020, Elsevier. f) Diagram illustrating the operation of the intermediate temperature Na–S battery. Reproduced with permission.^[^ ^99^ ^]^ Copyright 2019, the Electrochemical Society. g) Structure diagram of high high‐temperature Na–S battery. Reproduced with permission.^[^ ^79^ ^]^ Copyright 2017, John Wiley and Sons. h) Distribution diagram of the corresponding phase of high high‐temperature Na–S battery at different voltage stages. Reproduced with permission.^[^ ^94^ ^]^ Copyright 2008, Royal Society of Chemistry. i) Phase diagram of an Na–S battery system at three temperatures (HT, IT, and RT). Reproduced with permission.^[^ ^99^ ^]^ Copyright 2019, the Electrochemical Society.

Sulphur cathodes are the first to be used in sodium‐ion batteries due to their wide distribution, low toxicity, and high abundance. Their applications are divided into high‐temperature, medium‐temperature, and low‐temperature sodium–sulfur batteries.^[^ ^42^, ^43^ ^]^ In **Figure** **6**a–d, five characteristics of room temperature Na–S batteries, high temperature Na–S batteries, lithium–sulfur (Li–S) batteries, and sodium‐ion batteries were analyzed.^[^ ^86^ ^]^ Compared with Li–S batteries, Na–S batteries have higher actual energy density and longer cycle life.^[^ ^87^ ^]^ However, the safety issues of HT Na–S batteries have limited their application in electric vehicles.^[^ ^80^ ^]^ To achieve a broader range of applications for high‐energy‐density batteries, Na–S batteries have been continuously improved, leading to the development of intermediate temperature sodium–sulfur batteries and room temperature sodium–sulfur batteries (RT Na–S). The current RT Na–S not only overcomes the safety issue but also can be applied in the grid and even electric vehicles, making them the most promising SIBs.^[^ ^44^, ^88^ ^]^ This section will focus on the reaction mechanisms of the HT Na–S, IMT Na–S, and RT Na–S battery.

HT Na–S batteries operate at 300–350 °C and have been commercialized for energy storage devices due to their high energy density, long cycle life, and high efficiency.^[^ ^89^ ^]^ In the HT Na–S battery system (Figure 6e), molten sodium (*Tm* = 98 °C) serves as the negative electrode, while molten sulfur (*Tm* = 115 °C) inside the container acts as the positive electrode, and sodium‐β alumina ceramics act as the electrolyte. This ceramic electrolyte exhibits high ionic conductivity and is in close contact with the electrodes, resulting in lower ohmic polarization and thus reducing overall battery polarization. These batteries generally have a tubular structure, as shown in Figure 6g.^[^ ^90^, ^91^ ^]^ The reaction mechanism of HT Na–S batteries has been studied for decades, revealing the charge/discharge process, which can be summarized by the overall charge/discharge reaction equation:^[^ ^92^ ^]^

(8) $$2\mathrm{Na}+S⇌Na_{2}S_{x}\left(3\leq X\leq 5\right)\;E=2.0-1.78\;V\;\mathrm{at}\;350\;C$$

During operation, all active substances are in the molten state, and the melting points of Na_2_S_x_ (X ≥ 3) are below 300 °C.^[^ ^93^ ^]^ During the discharge process, at ≈2.08 V, S coexists with the first converted polysulfide Na_2_S_5_. As the voltage decreases, S and Na_2_S_5_ react with Na to form Na_2_S_4_, respectively, converting the two‐phase region formed by the immiscibility of S and Na_2_S_5_ into a single‐phase region, ultimately producing Na_2_S_3_ at 1.78 V (see Figure 6h).^[^ ^94^ ^]^ If deeply discharged, further solid Na_2_S_2_ is formed, which increases anodic resistance and prevents any further discharge reaction.^[^ ^95^ ^]^ Additionally, the high melting points of Na_2_S_2_ (Tm = 470 °C) and Na_2_S (Tm = 1168 °C) lead to the precipitation of solid Na_2_S_2_ and Na_2_S on the surface of the molten cathode and electrolyte if the discharge continues at 1.78 V. Therefore, HT‐Na/S batteries should not be discharged further at 1.78 V.^[^ ^96^ ^]^ As a result, the HT Na–S battery system (557 mAh g^−1^) can only achieve one‐third of the theoretical capacity of S, which is the maximum specific capacity of S to Na_2_S_3_. However, due to the battery's ability to undergo thousands of deep cycles and its low self‐discharge, the HT Na–S battery has been widely used in power grids and energy storage systems.

Compared with HT Na–S batteries, the new generation of intermediate‐temperature sodium–sulfur batteries (IMT Na–S, operating at 120 to 300 °C) offer advantages such as enhanced safety, scalability, and high cycling stability.^[^ ^97^, ^98^ ^]^ Figure 6f illustrates the working principle of IMT Na–S batteries.^[^ ^99^ ^]^ The reaction mechanism of IMT Na–S batteries is fundamentally similar to that of HT Na–S batteries (as described in Equation 8), but the chemicals involved in the sulfur reduction differ in the final step. As shown in Figure 6i, which compares the chemical processes of the three Na–S battery types against their operating temperatures,^[^ ^99^ ^]^ polysulfides exist in both molten and solid states within the mid‐temperature range of 120–300 °C. Additionally, the battery design features discs to enhance power output. It's worth noting that in IMT Na–S batteries, BASE exhibits higher ionic conductivity than in room temperature sodium–sulfur (RT Na–S) battery systems, resulting in faster charge and discharge rates.^[^ ^100^ ^]^ However, issues such as the corrosion and dissolution of sodium polysulfides persist at moderate temperatures.^[^ ^93^ ^]^

**Figure 7:**
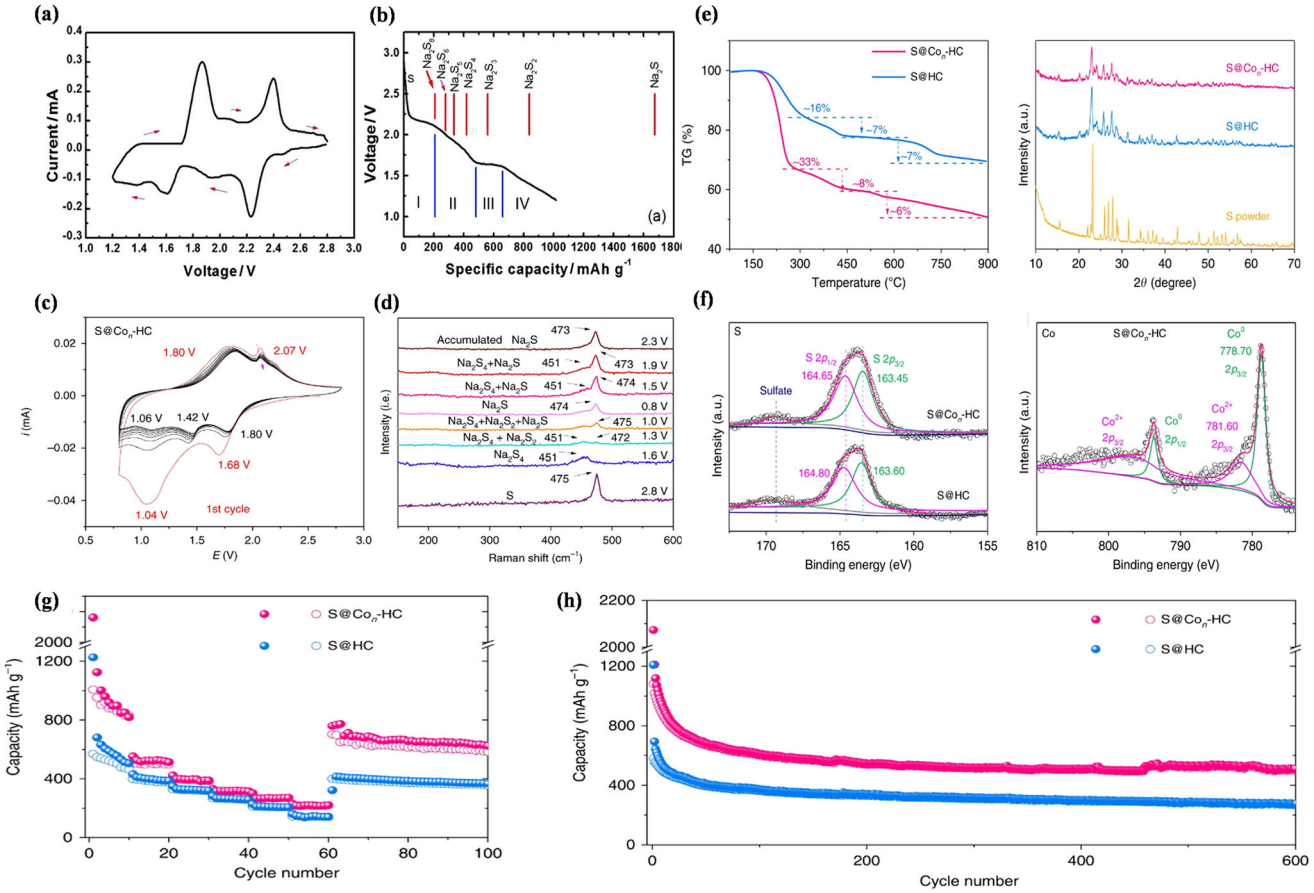
a) The CV of the Na–S battery operating at ambient temperature, and b) typical discharge curves in Na–S batteries at room temperature. Reproduced with permission.^[^ ^104^ ^]^ Copyright 2014, Wiley‐VCH. The mechanism of RT Na–S@Con‐HC batteries was characterized by c) cyclic voltammetry and d) in situ Raman spectroscopy. Thermogravimetric analysis of RT Na–S@Con‐HC batteries was performed using e) X‐ray diffraction and f) X‐ray photoelectron spectroscopy. g) Cycle performance and h) rate performance of S@Con‐HC and S@HC. Reproduced under the terms of the CC‐BY 4.0 license^[^ ^108^ ^]^ Copyright 2018, Bin‐Wei Zhang et al.

In RT Na–S batteries, the theoretical state of S is cyclic S_8_ because among the 30 solid isomers of S, the cyclic ortho configuration S (β‐sulfur, S_8_) is the most stable isomer at room temperature.^[^ ^101^ ^]^ During battery cycling, Na–S batteries operate through a series of electrochemical conversion reactions between Na and S ions. During the discharge process, the sodium metal anode undergoes an oxidation reaction to generate Na^+^ and electrons. Here, Na^+^ travels from the anode side to the cathode side through the organic electrolyte (internal circuit), while electrons reach the cathode through the external circuit. Subsequently, the S cathode accepts Na^+^ and electrons, resulting in the ring‐opening of S_8_,^[^ ^79^, ^102^ ^]^ which leads to the formation of discharge products. However, the conversion reaction for the formation of Na_2_S from S_8_ is not a one‐step reaction; it requires multiple steps, and different intermediates are formed.^[^ ^103^ ^]^ Due to the limitations of BASE in Na–S batteries, the electrolyte of RT Na–S batteries generally employs organic solvents (e.g., ethylene carbonate/propylene carbonate) and sodium salts (e.g., NaClO_4_). The CV and discharge curves in organic electrolytes are shown in **Figure** **7**a.^[^ ^104^ ^]^ As depicted in Figure 7b, the operating voltage range of RT Na–S batteries typically spans from 0.8 to 2.8 V. There are two prominent reduction peaks at 2.20 and 1.65 V in Na–S batteries, corresponding to the two discharge plateaus in the discharge curve. Therefore, the entire discharge curve is divided into four regions,^[^ ^23^, ^105^ ^]^ and the specific process of the discharge reaction of RT Na–S batteries (Figure 7a) is as follows:^[^ ^104^ ^]^

(9) $$Na_{2}S_{2}+2Na^{+}+2e^{-}\to 2{\mathrm{Na}}_{2}S$$

Region I is a high‐voltage plateau with a voltage interval of 2.2 to 2.8 V. According to Equation (9), a solid–liquid reaction occurs at 2.2 V.^[^ ^106^ ^]^ The solid monomeric sulfur (β‐S_8_) is reduced to a long‐chain sodium polysulfide, and the insoluble substance S is converted into the soluble substance Na_2_S_8_.

(10) $$3Na_{2}S_{2}+2Na^{+}+2e^{-}\to 8{\mathrm{Na}}_{2}S_{6}$$

(11) $$5Na_{2}S_{8}+6Na^{+}+6e^{-}\to 8{\mathrm{Na}}_{2}S_{5}$$

(12) $$Na_{2}S_{8}+6Na^{+}+2e^{-}\to 8{\mathrm{Na}}_{2}S_{4}$$

Region II is a tilted region with a voltage interval of 2.20 to 1.65 V. According to Equations (10)–(12), the process occurs as a liquid–liquid reaction, where soluble long‐chain Na_2_S_8_ is converted to soluble medium‐chain Na_2_S_4_. Additionally, Na_2_S_6_ and Na_2_S_5_ may form through other subtle reactions, involving a mutual conversion among soluble Na_2_S_6_, Na_2_S_5_, and Na_2_S_4_.^[^ ^96^ ^]^ Compared to lithium polysulfide (Li_2_S_x_, 4≤x≤8) generated during the discharge process of Li–S batteries, the generated sodium polysulfide (Na_2_S_x_, where 4 ≤ x ≤8) is more soluble in the liquid electrolyte and exhibits a stronger shuttle effect.

(13) $$3Na_{2}S_{4}+2Na^{+}+2e^{-}\to 4Na_{2}S_{3}$$

(14) $$Na_{2}S_{4}+2Na^{+}+2e^{-}\to 2Na_{2}S_{2}$$

Region III is a low‐pressure plateau region with a voltage of ≈1.65 V. According to Equations (13) and (14), a liquid–solid reaction occurs at this stage, where dissolved Na_2_S_4_ is converted to Na_2_S_3_ or Na_2_S_2_ intermediates, transitioning from soluble medium‐chain Na_2_S_4_ to insoluble medium‐ and short‐chain sodium polysulfides (NaPSs).^[^ ^80^ ^]^

(15) $$Na_{2}S_{2}+2Na^{+}+2e^{-}\to 2Na_{2}S$$

Region IV is the second tilted region, with a voltage interval of 1.65–1.20 V. According to Equation (15), a solid–solid reaction occurs at this stage, in which insoluble short‐chain Na_2_S_2_ is converted to the final product, Na_2_S.^[^ ^107^ ^]^ As shown in Figure 7c,d, taking the RT‐Na/S@Con‐HC battery as an example, the overall Na–S battery conversion reaction mechanism is indeed satisfied. Furthermore, thermogravimetric analysis (Figure 7e,f) and cycling rate performance (Figure 7g,h) demonstrate greater advantages than those of HT Na–S and IMT Na–S.^[^ ^108^ ^]^

**Figure 8:**
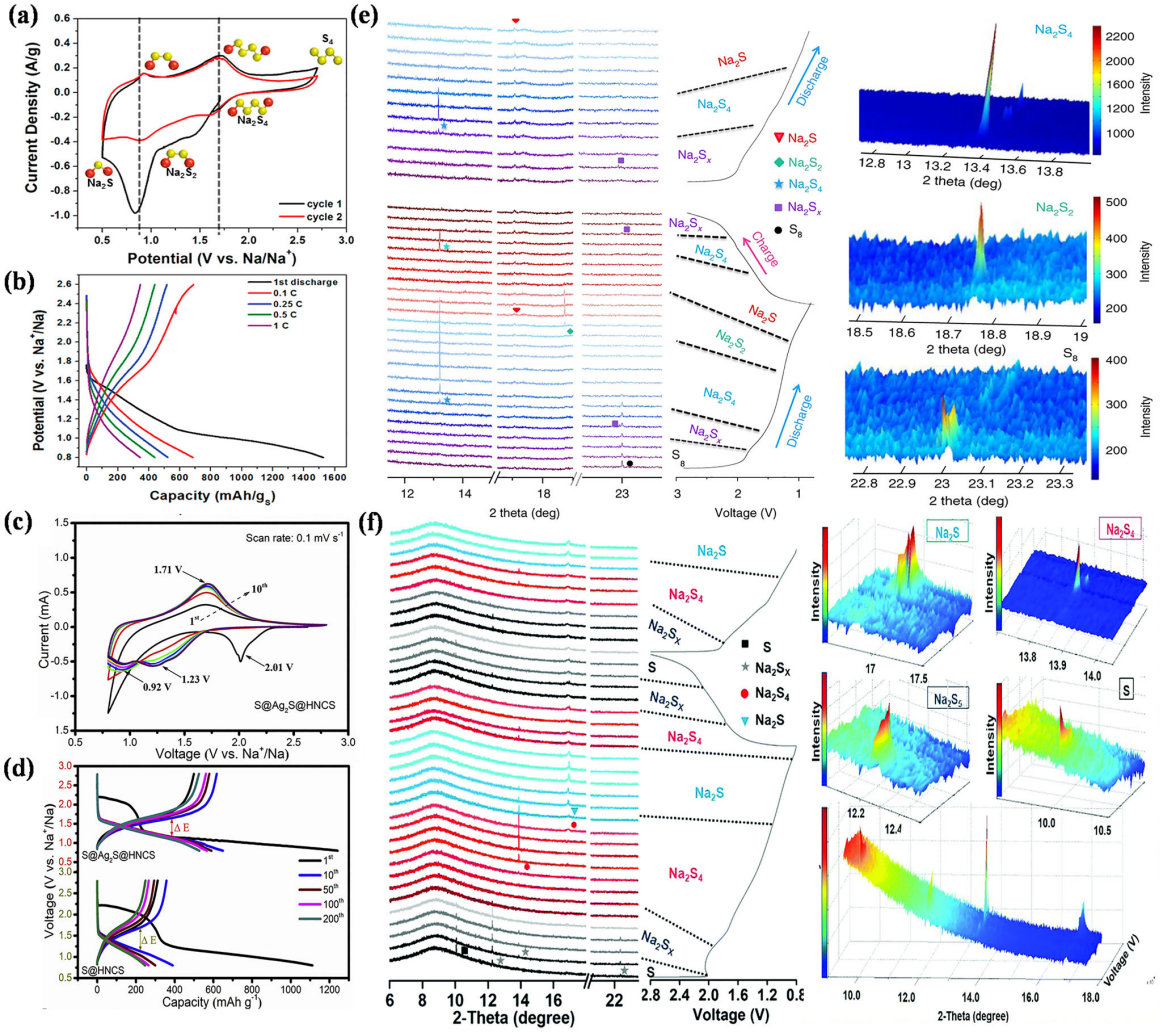
a) CV and b) galvanostatic charge/discharge profiles at various charging rates of sodium–sulfur battery with an S@C cathode. Reproduced with permission.^[^ ^113^ ^]^ Copyright 2019, ACS Publications. c) Charge–discharge curve and d) CV of sodium–sulfur battery with S@Ag_2_S@HNCS as the cathode. Reproduced under the terms of the Creative Commons CC‐BY license.^[^ ^111^ ^]^ Copyright 2021, Zichao Yan et al. In situ synchrotron XRD patterns of charged and discharged and contour plots of XRD patterns with selected theta for intermediate products of the room temperature sodium–sulfur battery, including e) S@Con‐HC and f) CN/Au/S as the cathode. Reproduced under the terms of the CC‐BY 4.0 license.^[^ ^108^ ^]^ Copyright 2018, Bin‐Wei Zhang et al. Reproduced with permission.^[^ ^112^ ^]^ Copyright 2008, RSC Publishing.

Therefore, during the conversion process, S electrodes typically undergo a solid–liquid–solid phase transition, and the conversion process is complex, especially in region II,^[^ ^109^ ^]^ where the reactions in this phase occur simultaneously and are influenced by the polysulfide chemical equilibrium. Since Na_2_S_2_ and Na_2_S_3_ coexist in region III, and only Na_2_S_2_ can be converted to Na_2_S, the capacity and discharge voltage of this phase depend on the competition between Equations (13) and (14). Additionally, the slow kinetics in region IV, due to the non‐conducting Na_2_S_2_ and Na_2_S, make this region potentially subject to hyperpolarization.^[^ ^79^, ^110^ ^]^ As shown in **Figure** **8**a–d, the CV curves and charge–discharge voltage diagrams of S@C cathodes and S@Ag_2_S@HNCS cathodes are similar to those of the original elemental sulfur cathodes, indicating that the conversion reaction adheres to the basic reaction mechanism.^[^ ^109^, ^111^ ^]^ Figure 8 presents the in situ synchrotron XRD patterns of room temperature Na–S batteries using different catalysts. For Na–S batteries employing S@Con‐HC as the cathode (Figure 8e), no S_8_ was found after charging, indicating that such Na–S batteries underwent a partially reversible conversion reaction. Simultaneously, it is also observed that the charging and discharging reaction processes of these Na–S batteries differ.^[^ ^108^ ^]^ Furthermore, in Na–S batteries with CN/Au/S as the cathode (Figure 8f), Na_2_S_4_ can be completely converted to S_8_.^[^ ^112^ ^]^ Although the two cells are both room‐temperature Na–S batteries, their conversion reaction processes vary. Therefore, the reaction process of Na–S batteries is quite complicated.

Overall, the sodium anode oxidizes to generate Na⁺ ions, which subsequently react with the sulfur cathode. This initiates the sequential formation of various intermediates until final reduction products are formed, constituting the complete conversion reaction pathway of the sulfur cathode. Under temperature variations, polysulfides (Na_2_S_x_) may form and undergo phase transitions. Particularly at lower temperatures, the conversion process becomes more complex, with ambiguous reaction sequences within specific voltage ranges. Based on the above mechanism, these batteries offer advantages including high efficiency, large scale, long cycle life, and high energy density. However, they face significant challenges such as the shuttle effect, substantial volume expansion, sulfur's low intrinsic conductivity, and sluggish conversion kinetics. Presented in **Table** **2** is a summary of sulfur cathode applications in sodium–sulfur batteries, featuring specific capacities and critical material properties reported in recent literature. Researchers have developed countermeasures, including structural confinement of polysulfides, catalysts to accelerate reversible reactions, and composite metallic materials to suppress side reactions. These modifications remain insufficient to fully address current challenges. Continuous improvement of cathodes is still imperative. The future application of Na–S batteries is expected to shift from grid storage devices to electric vehicles and specialized low‐temperature environments. This evolution represents a critical advancement toward batteries with enhanced safety, improved efficiency, and higher energy densities.

**Table 2:** Performance comparison of cathode materials in Na–S batteries.

Types of materials	Morphology	Current density [mA g^−1^]	Initial capacity [mAh g^−1^]	Cycle number	Capacity after circulation [mAh g^−1^]	Decay per cycle [%]	Capacity retention [%]	Refs.
S/MoN@CNFs	Nanofiber web	200	1170.00	100	990.00	0.1540	84.60	[22]
FeS2@ NCMS/S	Porous carbon microspheres	100	1471.00	300	524.00	0.2150	35.60	[114]
ACC‐40S	Slit ultramicropore	1000	720.00	2000	684.00	0.0025	95.00	[115]
S@GeOx/NC	Hierarchical porosity structure along with micro‐mesoporosity	100	1247.00	100	1017.00	0.1840	81.60	[116]
PAA‐based S‐PAN	Discrete globular particles	1000	1195.00	1000	1000.00	0.0160	84.00	[117]
S@CoPCo/NCNHC	Hollow cage structure	100	1101.00	220	592.00	0.2100	53.80	[118]
Co1–ZnS/C@S	Microsphere	100	1070.00	500	640.00	0.0800	60.00	[119]
S‐cZIF‐8	Stratified pore structure	0.1 C	1219.70	200	602.80	0.2530	49.40	[120]
SD/S1	Pore structure	0.5 C	903.00	200	674.00	0.1270	74.60	[121]
CNF/CoFe_2_O_4_/S	Fibrous reticular structure	0.1 C	1097.00	2800	829.70	0.0087	75.64	[122]
S@HPC/Mo_2_C	Hierarchical porous hollow carbon polyhedrons	200	1408.00	120	1098.00	0.1830	78.00	[123]
MoTe_2_/S	3D flower‐like	0.1 C	1015.00	300	1004.00	0.0030	99.00	[124]
NOC@MoS_2_	Flower‐like porous structure	1000	721.00	1000	390.00	0.0600	54.00	[125]
S/CoMoO_4_@rGO	Nanoflowers	100	662.00	100	520.00	0.2140	78.60	[126]
Ni@NPC/S	Stratified pore	100	830.10	300	633.70	0.0789	76.30	[127]
S@NPC‐700	Micropore/nanopore structure	0.1 C	1234.20	400	496.00	0.1500	40.00	[128]
S@Co,N‐MPC‐10%	Micropore	0.1 C	1335.09	200	1130.82	0.0500	84.70	[129]
SeS_2_/V_2_O_3_@C	Porous carbon nanorods	1000	1319.00	700	405.00	0.0990	30.70	[49]
S/P‐Fe_2_O_3_@Fe‐Ppy	Porous nanocube	500	1122.00	500	841.50	0.0500	75.00	[130]
S@MHCS	Mesoporous hollow nanospheres	0.1 C	1001.00	100	701.00	0.3000	70.00	[131]
Fe/FeS_2_‐PC@S	3D porous microspheres	5000	823.00	600	516.00	0.0700	62.70	[132]
MoS_2_/rGO/S	Flower‐like	2 C	350.00	1000	190.00	0.0457	54.30	[133]

### 3.2 Reaction Mechanism of Oxygen Cathodes

**Figure 9:**
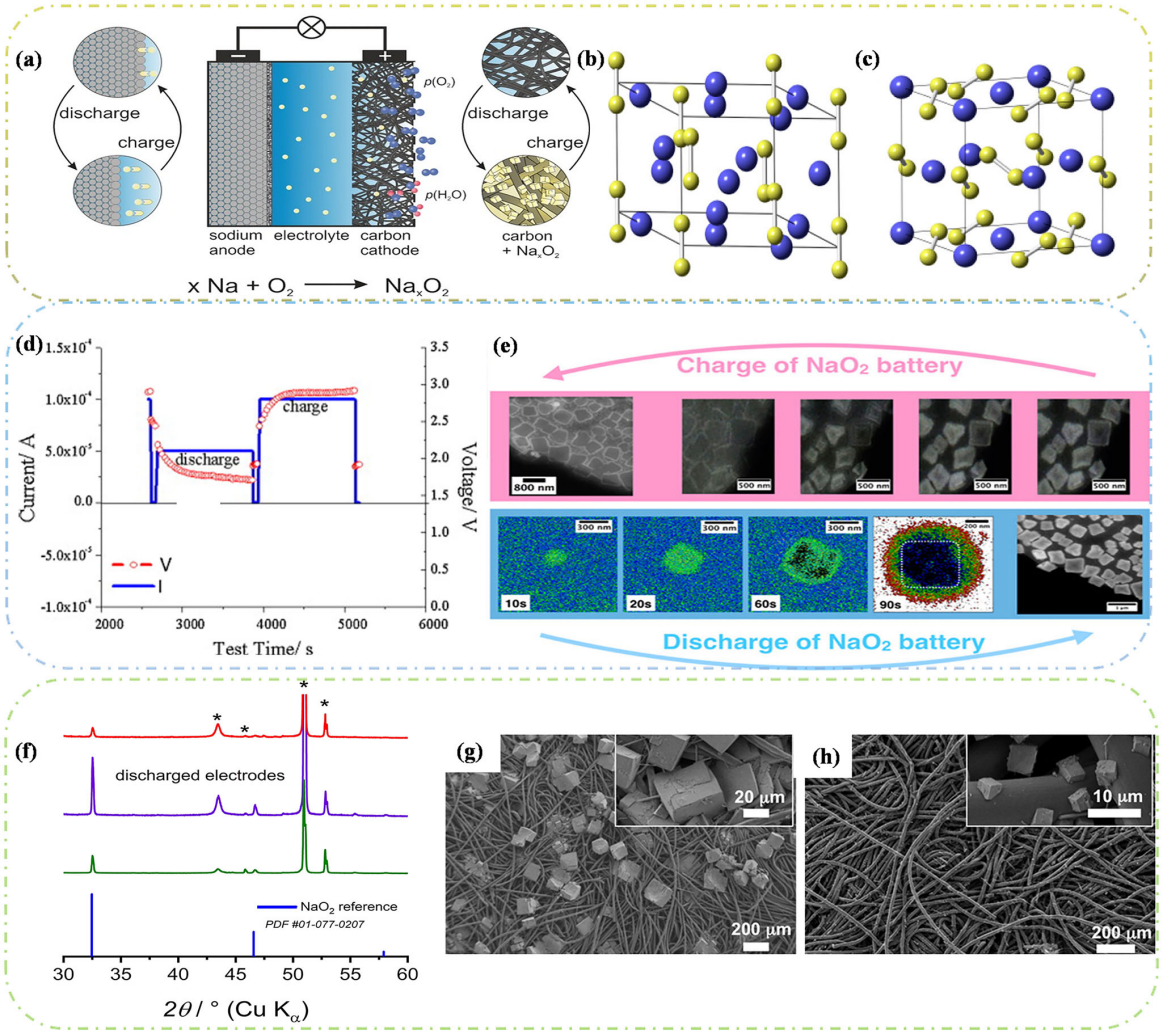
a) Working principle diagram of Na–O_2_ battery. Reproduced with permission.^[^ ^144^ ^]^ Copyright 2016, John Wiley and Sons. b) Crystal structures of hexagonal Na_2_O_2_ and c) Crystal structure of cubic (pyrite) NaO_2_. The yellow spheres represent oxygen atoms, while the blue spheres represent sodium atoms. Reproduced with permission.^[^ ^51^ ^]^ Copyright 2015, American Chemical Society. d) Discharge/charge curve of the Na–O_2_ battery. Reproduced with permission.^[^ ^138^ ^]^ Copyright 2011, Journal of Power Sources. e) Schematic diagram of the discharge and charging process of Na–O_2_ battery, as observed by transmission electron microscopy (TEM). Reproduced with permission.^[^ ^141^ ^]^ Copyright 2018, American Chemical Society. f) The PXRD pattern corresponding to the positive electrode of the Na–O_2_ battery. Three current densities are used: 0.025 mA cm^−2^ (green line), 0.25 mA cm^−2^ (purple line), and 0.4 mA cm^−2^ (red line). Peaks marked with a black asterisk (*) correspond to the sample holder and GDL. An SEM image of the positive electrode of NaO_2_‐based Na–O_2_ battery after discharge at g) 0.025 mA cm^−2^ and h) 0.4 mA cm^−2^. Reproduced under the terms of the Creative Commons CC BY license.^[^ ^142^ ^]^ Copyright 2022, Zarko P. Jovanov, Lukas Lutz, Juan G. Lozano, et al.

Oxygen Cathodes as a clean and sustainable energy source, their use as a cathode in sodium ion batteries is a challenging technology. Unlike other alkali metal–air batteries, the two main products of sodium–oxygen batteries (**Figure** **9**a) studied so far are sodium superoxide (the NaO_2_ structure is shown in Figure 9b)^[^ ^134^, ^135^ ^]^ and sodium peroxide (the Na_2_O_2_ structure is shown in Figure 9c),^[^ ^136^ ^]^ and the reaction mechanism of their batteries is slightly different.^[^ ^51^ ^]^ Therefore, this section will analyze the mechanism of Na–O_2_ batteries from the perspective of the discharge products.

NaO_2_‐based Na–O_2_ batteries exhibit high energy efficiency and energy density, with a theoretical specific capacity of 1105 Wh kg^−1^, which is six times higher than that of lithium‐ion (Li‐ion) batteries, making them a promising alternative to Li‐ion batteries.^[^ ^52^ ^]^ Since lithium and sodium belong to the same group in the periodic table, researchers initially believed that Na–O_2_ and Li–O_2_ batteries share the same reaction mechanism. A typical Na–O_2_ battery consists of a porous cathode with sufficient oxygen, a sodium anode, a separator between the two, and an electrolyte.^[^ ^137^ ^]^ However, ongoing exploration has revealed differences between the two types of batteries. First, the discharge/charge curve of Na–O_2_ batteries is illustrated in Figure 9d.^[^ ^138^ ^]^ The charging and discharging overpotentials of Na–O_2_ batteries are significantly lower than those of Li–O_2_ batteries. Consequently, Li–O_2_ batteries require more energy during charging and discharging, leading to unstable performance and poor cycling stability. Second, the operating voltage of Na–O_2_ batteries is lower than that of Li–O_2_ batteries. Most importantly, Li–O_2_ batteries are ultimately converted to lithium peroxide (Li_2_O_2_), and Na–O_2_ batteries experience competition between two products.^[^ ^139^ ^]^ The overall electrochemical reactions at the O_2_ cathode include the oxygen reduction reaction (ORR) and the oxygen evolution reaction (OER), analogous to the charge/discharge reactions in Li–O_2_ batteries.^[^ ^140^ ^]^ In NaO_2_‐based Na–O_2,_ analogous to the charge/discharge reactions in Li–O_2_ batteries.^[^ ^140^ ^]^ In NaO_2_‐based Na–O_2_ batteries, ORR involves a single electron transfer, where a single Na^+^ combines with O_2_^−^ precipitated from the cathode to form active sodium oxide. Similarly, OER reversibly decomposes the discharge products to produce oxygen(Figure 9e).^[^ ^141^ ^]^ The presence of the discharge product NaO_2_ has been confirmed in reports of Na–O_2_ batteries, as shown in Figure 9f.^[^ ^142^ ^]^ Importantly, NaO_2_ is a stable major product, regardless of the type of cathode used. The chemical reaction process is illustrated in Figure 9d:^[^ ^50^, ^143^ ^]^

(16) $$Na^{+}+2e^{-}+O_{2}⇌\mathrm{Na}O_{2}$$

The conversion reaction occurs with NaO_2_ as the discharge product, forming NaO_2_ in the form of cubes or nanorods at 2.263 V. Typical morphology is seen in Figure 9g,h, with cubes stacking on the cathode surface on a micron scale. In conjunction with past studies, it has been found that the NaO_2_ phase possesses a variety of polycrystalline states. Below 196 K, the stable structure of NaO_2_ is a tetragonal with a space group Pnnm. Between 196 and 223 K, the stable phase resembles that of NaCl rock salt (space group $\mathrm{Pa}\bar{3}$), with four oxydimers occupying the position of Cl^−^ in the structure, will follow different ⟨111⟩ directions. Above 223 K, while the oxygen dimers are still oriented along the ⟨111⟩ direction, the stable phase adopts a face‐centered cubic (FCC) structure with the space group $\mathrm{Fm}\bar{3}m$. Furthermore, the ionic conductivity in NaO_2_ is mediated by a mixture of negative sodium vacancies and positive oxygen dimer vacancies, as calculated by first principles.^[^ ^51^ ^]^ Therefore, at room temperature, the NaO_2_ crystal structure adopts an ordered $\mathrm{Pa}\bar{3}$ structure, as seen in Figure 9c, indicating that NaO_2_ is an insulator with a wide band gap.^[^ ^52^ ^]^ Due to the insulating nature of NaO_2_,^[^ ^145^, ^146^ ^]^ its crystal growth and decomposition mechanisms have been changed. Therefore, two routes for NaO_2_ crystal growth have been proposed. One is surface‐mediated, which, as the name suggests, involves the direct reduction and continuous deposition of O_2_ on the surface of a conversion‐type cathode using the outer surface of NaO_2_. The other is solution‐mediated, where the O_2_^−^ intermediate, first reduced from O_2_ through the cathode surface, grows by nucleation and crystallization with Na^+^ in solution.^[^ ^147^, ^148^ ^]^ Currently, numerous studies have demonstrated that the growth and decomposition of NaO_2_ crystals during charging and discharging processes satisfy the solution‐mediated mechanism.^[^ ^43^ ^]^ Moreover, with the introduction of a proton phase‐transfer catalyst, the reaction process can be expressed as follows in terms of the discharge process.

(17) $${\mathrm{HA}+O}_{2}^{-}⇌HO_{2}+A^{-}$$

(18) $$HO_{2}+Na^{+}⇌\mathrm{Na}O_{2}+H^{+}$$

**Figure 10:**
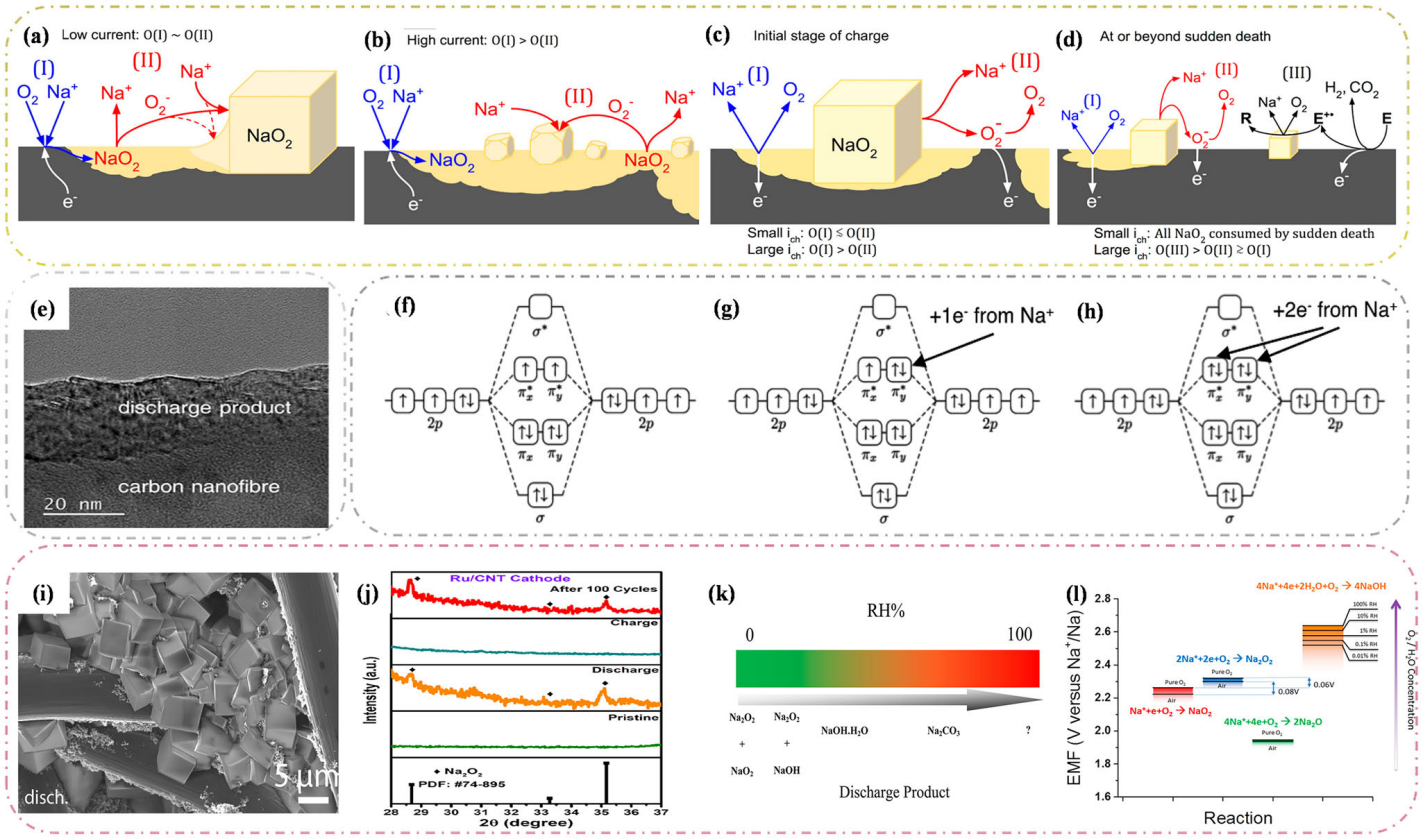
Schematic of a proposed mechanism based on solution‐mediated discharge processes at a) low current rates and b) high current rates. The proposed mechanism during charge c) before sudden death and d) following sudden death. Reproduced with permission.^[^ ^149^ ^]^ Copyright 2017, American Chemical Society. e) High‐resolution transmission electron microscopy (HRTEM) image of a thin film deposited on a carbon nanofiber electrode during discharge. Reproduced under the terms of the Creative Commons CC BY license.^[^ ^142^ ^]^ Copyright 2022, Zarko P. Jovanov, Lukas Lutz, Juan G. Lozano, et al. Molecular orbital (MO) diagrams for f) oxygen (O_2_^0^), g) superoxide (O_2_^1−^), and h) peroxide (O_2_^2−^) dimers. Reproduced with permission.^[^ ^51^ ^]^ Copyright 2015, American Chemical Society. i) Soluble NaO_2_ that may be formed when Na–O_2_ batteries were detected using a rotating ring electrode (RRDE) experiment. Reproduced with permission.^[^ ^175^ ^]^ Copyright 2015, American Chemical Society. j) All‐solid‐state Na–O_2_ batteries generate Na_2_O_2_ at room temperature. Reproduced with permission.^[^ ^170^ ^]^ Copyright 2022, American Chemical Society. k) Diagram illustrating the variation of discharge products with humidity in sodium–oxygen batteries (containing impurities); l) potential products present in sodium–oxygen batteries under pure oxygen or dry/wet air conditions. Reproduced with permission.^[^ ^173^ ^]^ Copyright 2015, American Chemical Society.

Protons are supplied by residual water molecules or acids in the electrolyte, which react with the reduction process at the cathode to form O_2_^−^, subsequently reacting at the cathode surface to produce HO_2_. The reaction in Equation (17) can occur because superoxide (O_2_^−^) is itself basic. Due to its solubility, H_2_O acts as a carrier into the solution, where it reacts with the Na^+^ to form the NaO_2_ crystals. As illustrated in **Figure** **10**, the solution‐mediated mechanism leads to the formation of large NaO_2_ crystals at a low rate(Figure 10a). As expected from the established theory of crystal growth, at high rates, NaO_2_ is more inclined to surface‐mediated film formation(Figure 10b), although the solution‐mediated mechanism still accounts for a significant portion of NaO_2_ formation.^[^ ^149^ ^]^ Similarly, the crystal decomposition during charging can be explained(Figure 10c,d).^[^ ^24^, ^52^, ^150^ ^]^

In contrast, the Na_2_O_2_‐based Na–O_2_ batteries have a higher theoretical specific capacity of 1602 Wh kg^−1^, which is nine times greater than that of Li‐ion batteries. However, the charging overpotential of Na–O_2_ batteries is not superior to that of Li–O_2_ batteries. There is an equal number of studies examining the discharge products NaO_2_ and Na_2_O_2_ in Na–O_2_ batteries, but there is a disagreement regarding the formation of Na_2_O_2_. One group of researchers suggests that NaO_2_ undergoes a disproportionation reaction to produce Na_2_O_2_^[^ ^143^, ^144^ ^]^ (see Equation 19), and this has been verified.^[^ ^146^ ^]^ Another part believes that Na_2_O_2_ can be generated directly through a two‐electron reaction as follows;^[^ ^151^, ^152^ ^]^

(19) $$2\mathrm{Na}O_{2}\to Na_{2}O_{2}+O_{2}$$

(20) $$2Na^{+}+2e^{-}+O_{2}⇌Na_{2}O_{2}$$

The Na–O_2_ battery produces Na_2_O_2_ at 2.33 V in the form of particles or thin films (Figure 10e), and as shown in Figure 9b, the Na_2_O_2_ crystals have a hexagonal structure of space‐based P62m.^[^ ^142^ ^]^ By adding some catalysts, researchers have successfully detected the presence of Na_2_O_2_ using XRD and Raman spectroscopy,^[^ ^153^, ^154^ ^]^ but in ether‐based electrolytes and hydrophobic carbon cathodes without catalysts, it could not be detected due to the low crystallinity and low content of Na_2_O_2_. In many Na–O_2_ battery studies, NaO_2_ is the main product, with only small amounts of Na_2_O_2_ that are difficult to detect. However, by removing all NaO_2_ in some form, researchers have been able to observe Na_2_O_2_. Jovanov et al.^[^ ^142^ ^]^ used a large amount of electrolyte to dissolve NaO_2_ and demonstrated through spectroscopy that Na_2_O_2_ forms during discharge in ether‐based Na–O_2_ batteries at high overpotentials. Unfortunately, the accumulation of small amounts of Na_2_O_2_ can lead to passivation and rapid degradation of the battery electrodes. During continuous cycling, Na_2_O_2_ continues to accumulate and becomes difficult to remove from the battery due to its inherent stability.^[^ ^155^ ^]^ To remove Na_2_O_2_, the battery must be charged at a high overpotential. Unfortunately, there is no conclusive evidence regarding the growth mechanism of Na_2_O_2_.

The discharge products of Na–O_2_ batteries remain uncertain, possibly due to the position of the elements.^[^ ^156^ ^]^ In alkali metal–oxygen batteries, lithium and potassium are in the same group, and the discharge product for Li–O_2_ batteries is lithium peroxide (Li_2_O_2_),^[^ ^157^, ^158^ ^]^ while K–O_2_ batteries produce potassium superoxide (KO_2_).^[^ ^159^, ^160^ ^]^ Thus, the presence of two discharge products makes sense for sodium, which is positioned between lithium and potassium. Considering all three alkali metal–oxygen batteries, it can be concluded that the stability of these superoxides follows this order: KO_2_ > NaO_2_ > LiO_2_.^[^ ^161^ ^]^ This creates challenges in studying the discharge products of Na–O_2_ batteries. Although both NaO_2_ and Na_2_O_2_ can be produced, their properties differ. Kinetically, the formation of NaO_2_ through a one‐electron reaction allows for a faster cycling rate than Na_2_O_2_ formed through a two‐electron transfer. Thermodynamically, the Gibbs free energy of lattice formation for Na_2_O_2_ is −449.7 kJ mol^−1^, slightly lower than that of NaO_2_ (−437.5 kJ mol^−1^) at 298 K,^[^ ^162^ ^]^ with an energy difference of only 12 kJ mol^−1^. This is also reflected in their standard electrode potentials (a 0.07 V difference), indicating that Na_2_O_2_ is theoretically more stable.^[^ ^163^ ^]^ However, in practice, thermodynamic stability is also influenced by the size of the grains formed. Nano‐sized NaO_2_ is more stable than Na_2_O_2_ due to its lower surface energy, favoring its growth.^[^ ^164^ ^]^ Consequently, determining which is more stable between the two is thermodynamically complex. Furthermore, the molecular orbital diagrams of O_2_, O_2_^−^, and O_2_^2−^ (Figure 10f–h) show that the two oxygen atoms undergo SP orbital hybridization and exhibit Paramagnetism due to the presence of unpaired electrons in the $\pi_{x}^{*}$ and $\;\pi_{y}^{*}$ antibonding orbitals.^[^ ^51^ ^]^ O_2_^−^ has nine electrons in the 2p orbital, resulting in one unpaired electron (located on the $\pi_{x}^{*}$ in Figure 10g), while O_2_^2−^ has no unpaired electron. According to the molecular orbital theory, the bond levels for O_2_, O_2_^−^, and O_2_^2−^bond levels are 2, 1.5, and 1, respectively. O_2_^−^ reduces the O─O atomic spacing from 1.6 to 1.35 Å compared to O_2_^2−^, making O_2_^−^ more magnetic. Consequently, O_2_^−^ is highly reactive and prone to side reactions with electrodes or electrolytes.^[^ ^165^ ^]^ Experimental results indicate that the composition of discharge products is closely related to the catalyst, gas components, and electrolyte.^[^ ^166^, ^167^ ^]^ For example, cubic crystals of NaO_2_ are produced in pure oxygen (Figure 10i),^[^ ^168^, ^169^ ^]^ thin films are formed in the presence of Na_2_O_2_ under dry air (Figure 10j),^[^ ^170^, ^171^ ^]^ and CO_2_ leads to the formation of Na_2_O_2_, Na_2_C_2_O_4_, and Na_2_CO_3_ (Figure 10k).^[^ ^172^ ^]^ In humid environments, NaOH is formed (Figure 10l),^[^ ^173^ ^]^ while using diethylene glycol dimethyl ether (DME) and sodium trifluoromethanesulfonate (NaSO_3_CF_3_) as electrolytes produces cubic NaO_2_ particles.^[^ ^174^ ^]^

In conclusion, the reaction mechanism of the Na–O_2_ batteries involves the conversion reaction between sodium and oxygen, leading to the accumulation of discharge products on the cathode surface. Although this process appears relatively straightforward, the composition of these products remains debated. Current consensus holds that the primary products are sodium superoxide (NaO_2_) and sodium peroxide (Na_2_O_2_). When NaO_2_ dominates as the discharge product, the batteries exhibit high round‐trip efficiency and exceptionally low charging overpotentials. However, NaO_2_’s inherent instability causes battery passivation and eventual failure. Conversely, Na_2_O_2_ typically demonstrates higher charging overpotentials and inferior cycling stability. These batteries offer compelling advantages, including abundant resources, high energy density, and environmental friendliness. Nevertheless, challenges such as short cycle life, insufficient capacity, and parasitic reactions significantly impede their development. As evidenced in **Table** **3**, Na–O_2_ batteries remain in an early developmental phase. Future research should focus on more in‐depth in situ characterization techniques and innovative electrode materials. This will enable efficient utilization of natural gas resources for next‐generation electric vehicle energy storage systems, align with sustainable development, and pave the way for high‐security, high‐energy‐density storage solutions.

**Table 3:** Performance comparison of cathode materials in Na–O_2_ batteries.

Air electrode	Morphology	Current density [mAh g^−1^]	Specific capacity [mAh g^−1^]	cycle number	Main discharge product	Electrolyte	Refs.
Ni/NaNO_3_/KNO_3_/CsNO_3_/SS	—	0.20 [mA cm^−2^]	16 [mAh cm^−2^]	400	Na_2_O_2_	Ni/salt β‐Al_3_O_2_	[24]
ArGO_N	3D structures with interconnected pore channels	100	6.61 [mAh cm^−2^]	128	NaO_2_	1 m NaClO_4_ DME	[135]
CoB‐900/CNT	Porous sheet structure	100	11482	124	Na_2_O_2_	0.5 m NaCF_3_SO_3_/TEGDME	[149]
m‐RuO_2_‐B‐rGO	Porous stratified structure	0.05 [mA cm^−2^]	0.50 [mAh cm^−2^]	>100	NaO_2_ and Na_2−x_O_2_	1 m NaCF_3_SO_3_ in tetraethylene glycol dimethyl ether	[154]
SPC	Pore	20	100	>100	NaOH	Na–β″‐Al_2_O_3_	[165]
C@NiCo_2_O_4_	Urchin‐like structure	50	6500	120	Na_2_O_2_	1 m NaClO_4_ in TEGDME	[176]
Co‐ECNCFs	Porous network	200	6102	112	NaO_2_ and Na_2_O_2_ ⚫2H_2_O	0.5 m NaCF_3_SO_3_ in TEGDME	[177]
PCS	Hierarchical porous spheres	1000	500	400	NaO_2_	0.5 m sodium triflate in diglyme	[178]
MnCo_2_O_4_/C	Loose porous spherical structure	0.10 [mA cm^−2^]	7709.40	130	composed of Na^+^ and O^+^	1.0 m NaClO_4_ in PC	[179]
ALD CNT@Co_3_O_4_	Spongy structure	150	715	13‐14	NaO_2_ and Na_2_O_2_	NaCF_3_SO_3_/DEGDME	[180]
COCT	Multihole	100	4687	62	NaO_2_ and Na_2_O_2_	0.5 mol L^−1^ NaCF_3_SO_3_ in TEGDME	[181]
Ru‐NPs@N‐rGO	—	—	500	100	NaO_2_	0.5 mol L^−1^ NaSO_3_CF_3_ in diethylene glycol dimethyl ether	[182]

### 3.3 Reaction Mechanism of Metal Halide Cathodes

Halogens are located in Group VIIA of the periodic table, among which F and Cl are more abundant in nature. Studies have focused on transition metal chlorides (MCl) and metal fluorides (MF) as cathodes for SIBs.^[^ ^183^, ^184^ ^]^ However, Br, I, and other metals forming metal halide cathodes have been less explored in the context of SIB cathodes.^[^ ^185^, ^186^ ^]^Metals such as Ni, along with high‐abundance options like Fe, Cu, and Zn, are commonly used as metal sources. Therefore, this section will focus on the reaction mechanisms of MCl and MF conversion‐type cathodes in SIBs and analyze the existing studies on Br and I.

#### 3.3.1 Transition‐Metal Chloride Battery

Among the transition‐metal halide cathode materials, MCl was the first conversion‐type cathode investigated for SIBs, referred to as sodium‐metal chloride batteries (ZEBRA).^[^ ^187^ ^]^ Similar to Na–S batteries, ZEBRA batteries consist of a molten sodium anode, MCl encapsulated by a molten salt electrolyte as the cathode, and a BASE solid electrolyte, which is required to operate at high temperatures to achieve sufficient ionic conductivity.^[^ ^188^ ^]^

**Figure 11:**
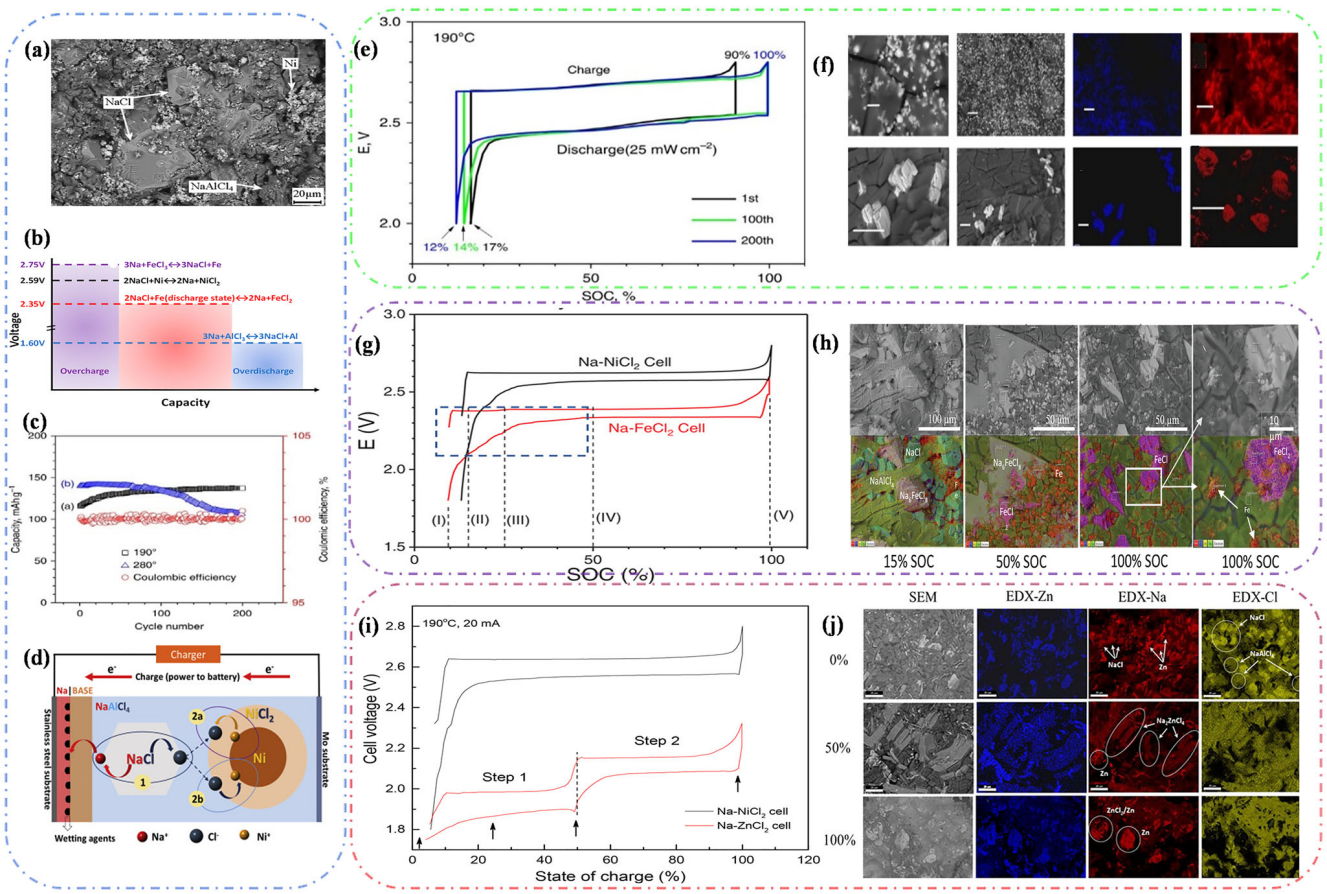
a) Microstructure of the zebra battery cathode surface. Reproduced with permission.^[^ ^191^ ^]^ Copyright 2012, Elsevier. b) Reaction mechanism of the Na‐FeCl_2_ battery during normal charge and discharge, as well as overcharge and overdischarge. Reproduced under the terms of the CC‐BY 4.0 license.^[^ ^53^ ^]^ Copyright 2021, Xiaowen Zhan et al. c) Capacity retention and Coulomb efficiency diagram of medium‐temperature Na‐NiCl_2_ battery. Reproduced under the terms of the CC‐BY 4.0 license.^[^ ^198^ ^]^ Copyright 2016, Guosheng Li et al. d) Schematic diagram of Na‐NiCl_2_ battery structure and charging reaction mechanism. Reproduced with permission.^[^ ^186^ ^]^ Copyright 2020, Elsevier. e) Voltage profiles for planar IT Na‐NiCl_2_ batteries, and f) SEM image of the cathode material of planar IT Na‐NiCl_2_ batteries. Reproduced under the terms of the CC‐BY 4.0 license.^[^ ^198^ ^]^ Copyright 2016, Guosheng Li et al. g) Typical voltage and charge distribution of Na‐NiCl_2_ and Na‐FeCl_2_ batteries, and h) backscattered SEM and EDX superimposed elemental mapping images of IT Na‐FeCl_2_ at different discharge capacities(15%, 50%,100%). Reproduced with permission.^[^ ^200^ ^]^ Copyright 2022, John Wiley and Sons. i) Voltage curve of the Na‐ZnCl_2_ battery compared to the Na‐NiCl_2_ battery at 190 °C, and j) SEM image of the fracture surface of the NaCl‐Zn cathode. Reproduced with permission.^[^ ^205^ ^]^ Copyright 2018, American Chemical Society.

Among the current research on ZEBRA batteries, the Ni‐based ZEBRA battery, also known as the sodium nickel chloride (Na‐NiCl_2_) battery, has been the most extensively studied. Its cathode consists of NiCl_2_ encapsulated by sodium tetrachloroaluminate (NaAlCl_4_).^[^ ^188^ ^]^ During the discharge process, Na^+^ migrates from the sodium anode through the solid electrolyte and reacts with NiCl_2_ at the cathode, resulting in the formation of Ni and NaCl on the cathode surface. However, the energy density of the Na–NiCl_2_ battery is only 100–120 Wh kg^−1^.^[^ ^189^ ^]^ **Figure** **11**a shows the cathode morphology at room temperature as observed using scanning electron microscopy,^[^ ^190^, ^191^ ^]^ and the discharge reaction of the Na‐NiCl_2_ battery at 300 °C is given by (Equation 21):^[^ ^192^, ^193^ ^]^

(21) $$\mathrm{NiC}l_{2}+2Na^{+}+2e^{-}⇌\mathrm{Ni}+2\mathrm{NaCl}$$

At 2.58 V, Na‐NiCl_2_ batteries directly form Ni particles and cubic NaCl crystals. The Ni particles increase at a decreasing rate with the number of battery cycles, growing to ≈5–10 µm before stabilizing.^[^ ^91^ ^]^ When overcharging occurs, NaAlCl_4_ in the outer cathode layer reacts with Ni to produce Na, AlCl_3,_ and NiCl_2_. Conversely, during over‐discharge, aluminum (Al) is produced. It is worth noting that even a small amount of overcharging or over‐discharging can lead to a degradation in battery performance, while significant overcharging or over‐discharging may result in battery failure.

FeCl_2_ cathodes offer potential advantages over NiCl_2_ cathodes. Notably, Cathode materials are easier to obtain due to high iron abundance.^[^ ^194^ ^]^ The mechanism of Na‐FeCl_2_ batteries is similar to that of Na‐NiCl_2_ batteries, as both produce Fe and NaCl during discharge. However, some batteries generate a variety of intermediate phases with the general formula Na_8‐x_FeX_8_. At 250 °C, a two‐phase reaction occurs (Figure 11b),^[^ ^53^ ^]^ which can be expressed as:

(22) $$4\mathrm{FeC}l_{2}+6Na^{+}+6e^{-}⇌Na_{6}\mathrm{FeC}l_{8}+3Fe$$

(23) $$Na_{6}\mathrm{FeC}l_{8}+2Na^{+}+2e^{-}⇌8NaCl+Fe$$

Briefly, the Na‐FeCl_2_ battery reacts from needle‐like FeCl_2_ crystals to produce rock salt‐structured Na_6_FeCl_8_ crystals with defective $\mathrm{Fm}\bar{3}m$ groups at 2.353 V, and the presence of Na_6_FeCl_8_ has been observed experimentally.^[^ ^195^ ^]^ Additionally, 1/8 of the octahedral sites were found to be empty, and upon comparison with the ion arrangement diagram of FeCl_2_, the distribution of Cl^−^ and the occupancy of Fe^−^ are extremely similar. This similarity facilitates the first and rapid conversion of FeCl_2_ to Na_6_FeCl_8_. When the voltage is decreased to 2.341 V, the final product, cubic NaCl crystals, is formed. This structure is the same as that of Na_6_FeCl_8_, suggesting that NiCl_2_ reacts with FeCl_2_ through the same reaction mechanism, although the reaction process of FeCl_2_ is more complex and produces an intermediate phase. Similarly, when the Na‐FeCl_2_ battery reacts at 350 °C, a three‐phase reaction occurs,^[^ ^196^ ^]^ producing Na_2_FeCl_4_ and Na_2_Fe_3_Cl_8_ crystals. Their structural phases are completely different from those of Na_6_FeCl_8_, being orthorhombic (Pbam) and triangular ($R\bar{3}m$) phases, respectively. However, compared with NaCl, the Cl^−^ array in these products is influenced by the array of Cl^−^ in NaCl.

To achieve further market penetration of Na‐MH batteries, intermediate temperature sodium‐metal halide (IT Na‐MH) batteries have gained strong interest due to breakthroughs in ceramic electrolytes, which have facilitated their development.^[^ ^197^ ^]^ These batteries, also known as planar Na‐MH batteries, reduce the operating temperature to 190 °C, providing high energy density (Figure 11c).^[^ ^198^ ^]^ Notably, the overall redox reactions of the mid‐temperature Na‐NiCl_2_ and Na‐FeCl_2_ batteries remain the same as those at high temperatures (the comparable view of the previous content will not be repeated in the later description).^[^ ^184^, ^199^ ^]^ The specific energy density of planar Na‐NiCl_2_ batteries (Figure 11d) is 35 Wh kg^−1^, which is lower than that of conventional tubular Na‐NiCl_2_ batteries (95–120 Wh kg^−1^) (Figure 11e,f),^[^ ^186^, ^198^ ^]^ while the specific energy density of planar Na‐FeCl_2_ batteries can exceed 295 Wh kg^−1^(Figure 11g,h).^[^ ^200^, ^201^ ^]^

During this period, ZnCl_2_ cathode materials were significantly developed, providing a theoretical basis for further exploration of other metal chlorides.^[^ ^202^ ^]^ Due to the complex chemistry of the Na‐ZnCl_2_ battery, the cycling process involves the formation of a liquid phase (requiring temperatures >253 °C). This causes the temperature to gradually decrease, progressing from 300 °C to 280 °C, then to 240 °C, and finally reaching 190 °C.^[^ ^203^ ^]^ At 280 °C, the Na‐ZnCl_2_ battery develops four different phases: solid NaCl, solid Na_2_ZnCl_4_ (ZnCl_2_ mole fraction of 0.33), a salt–liquid phase (with a ZnCl_2_ mole fraction of 0.62–0.78), and solid ZnCl_2_, which occurs in four steps.^[^ ^55^ ^]^ In contrast, at 190 °C, two steps and three solid phases are experienced, as clearly illustrated by the battery charge–discharge curve (Figure 11i). Unlike the single platform of the Na‐NiCl_2_ battery, which is also divided into two regions, as shown in the phase diagram, the discharge reaction process can be expressed as follows:^[^ ^204^ ^]^

(24) $$2\mathrm{ZnC}l_{2}+2Na^{+}+2e^{-}⇌Na_{2}\mathrm{ZnC}l_{4}+\mathrm{Zn}$$

(25) $$Na_{2}\mathrm{ZnC}l_{4}+2Na^{+}+2e^{-}⇌4\mathrm{NaCl}+\mathrm{Zn}$$

The reaction process of the Na‐ZnCl_2_ battery is extremely similar to that of the Na‐FeCl_2_ battery. At 2.13 V, a banded Na_2_ZnCl_4_ layer is produced at the cathode interface (Figure 11j), which leads to a fast reaction rate because it does not cover the cathode surface, resulting in excellent multiplicative performance.^[^ ^205^ ^]^ When the ZnCl_2_ is fully reacted, the second step of the reaction will be carried out directly. At 1.94 V, Zn precipitates from the Na_2_ZnCl_4_ intermediate. However, excessive discharge continues to react with NaAlCl_4_, and the resulting NaCl covers the Zn surface, causing cathode passivation and hindering the conversion reaction.

The CuCl_2_ cathode has been directly investigated at medium temperatures of 175 °C and 150 °C,^[^ ^206^ ^]^ offering the potential to achieve high‐energy‐density batteries. The same reaction mechanism as that of Na‐ZnCl_2_ batteries was clearly demonstrated for the two‐step conversion reaction of Na‐CuCl_2_ batteries, with the presence of Cu^2+^ converted to Cu^+^.^[^ ^207^ ^]^ The production of needle‐like CuCl_2_ after charging was observed, which showed excellent long‐term cycling performance.^[^ ^25^ ^]^

#### 3.3.2. Transition‐Metal Fluoride Battery

**Figure 12:**
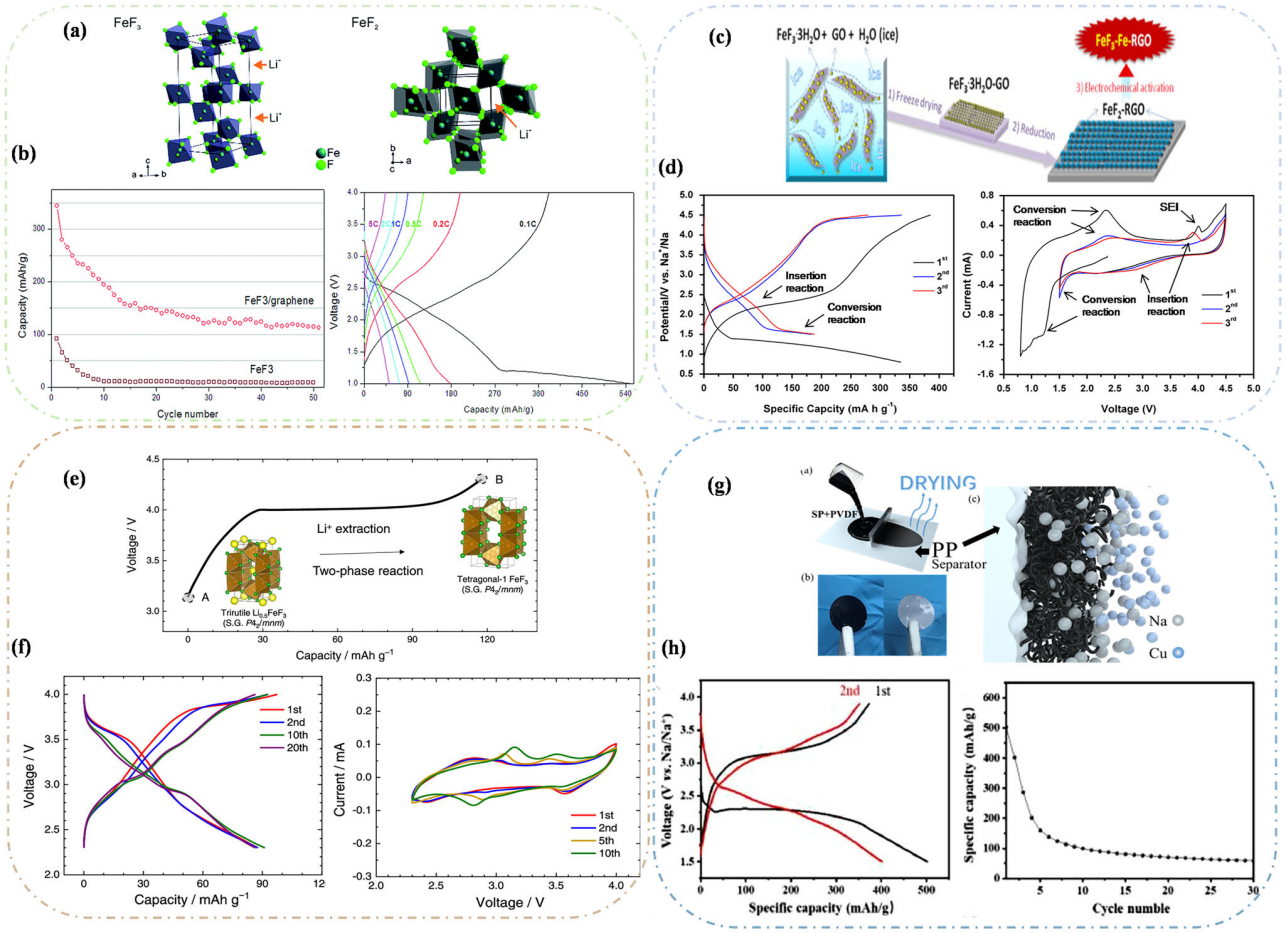
a) Structure diagrams of ReO_3_ for FeF_3_ and rutile of FeF_2_. Reproduced under the terms of the CC‐BY 4.0 license.^[^ ^217^ ^]^ Copyright 2013, Christoph P. Guntlin et al. b) Discharge curves of FeF_3_ cathode and FeF_3_/graphene cathodes, along with the cyclic performance of FeF_3_/graphene at different C magnifications. Reproduced with permission.^[^ ^212^ ^]^ Copyright 2015, RSC Publishing. c) Synthesis process diagram of FeF_3_ ‐Fe‐RGO composite material, and d) charge and discharge curve, along with voltage distribution for FeF_3_ ‐Fe‐RGO composites generated in situ by FeF_2_‐RGO composites. Reproduced with permission.^[^ ^215^ ^]^ Copyright 2014, Elsevier. e) Preparation diagram of the Na/tetragonal^−1^ FeF_3_ electrode. f) Charge–discharge curve and voltage distribution for the Na/tetragonal^−1^ FeF_3_ battery at a cutoff voltage of 2.3–4.0 V. Reproduced with permission.^[^ ^216^ ^]^ Copyright 2022, American Chemical Society. g) Preparation process for C coating separators, and h) charge–discharge curve and cycle performance of the CuF_2_‐KB electrode. Reproduced with permission.^[^ ^221^ ^]^ Copyright 2022, Elsevier.

Transition‐metal fluorides are promising cathode materials for SIBs due to their wide availability, low toxicity, and high capacity. Additionally, elemental fluorine has gained attention because of its high electronegativity and significance in energy conversion and storage.^[^ ^208^ ^]^ Commonly used cathodes for sodium‐metal fluoride batteries include metal trifluoride (MF_3_) and metal difluoride (MF_2_) (**Figure** **12**a), such as FeF_3_, CuF_2_, FeF_2_, CoF_2_, and so on.^[^ ^209^ ^]^ Compared with lithium‐metal fluoride batteries, the mechanism of metal fluorides as cathodes in sodium batteries has been less extensively studied, primarily focusing on iron‐based fluorides, while other studies have leaned toward modifications of conversion‐type cathodes.^[^ ^210^ ^]^ Therefore, this section will analyze the mechanism of MF_3_ and MF_2_ based on iron‐based materials.

Based on existing studies, the SIBs battery with iron‐based fluoride consists of a sodium anode, a composite of iron‐based fluoride as the cathode, an inorganic electrolyte, and a separator.^[^ ^211^ ^]^ Unlike ZEBRA batteries, first, Fe‐based fluoride has a wide bandgap (the energy bandgap of FeF_3_ is 5.96 eV) due to the high ionic nature of the Fe─F bond. A larger energy bandgap indicates greater insulating properties, leading to poor electrochemical performance and rapid capacity degradation. Consequently, the direct use of FeF_3_ as the cathode in batteries cannot meet the essential performance requirements (see Figure 12b).^[^ ^212^ ^]^ Therefore, iron‐based fluoride must be compounded with carbon materials, among others. In contrast, sodium metal fluoride batteries utilize inorganic electrolytes, such as commonly used propylene carbonate^[^ ^212^ ^]^ and ethylene carbonate/diethyl carbonate,^[^ ^213^ ^]^ while ZEBRA batteries typically employ a BASE. As a result, research on sodium metal fluoride batteries has been conducted at room temperature, with no studies on high‐temperature states. The metal trifluoride is exemplified by FeF_3_ ($R\bar{3}c$), and the discharge reaction process generally occurs within the range of 1.0–4.0 V as follows:^[^ ^212^, ^214^ ^]^

(26) $$\mathrm{Fe}F_{3}+Na^{+}+e^{-}⇌\mathrm{NaFe}F_{3}$$

(27) $$\mathrm{NaFe}F_{3}+2Na^{+}2e^{-}⇌\mathrm{Fe}+3\mathrm{NaF}$$

During the cycling of Na‐FeF_3_ batteries, the insertion of Na^+^ converts Fe^3+^ to Fe^2+^ within the voltage interval of 1.2–4.0 V. In the voltage interval of 1.0–1.2 V, the Fe^2+^ containing phase is converted to Fe metal. However, FeF_3_ can be synthesized through various methods, leading to differences in the exact reaction processes. Additionally, different reaction mechanisms can be observed depending on the number of cycles the battery undergoes, with variations occurring in the final phase of the reaction.

For example, Ma et al.^[^ ^215^ ^]^ prepared FeF_3_–Fe–RGO composites using metallic iron electrochemically generated in situ from FeF_2_ grains and active FeF_3_, as illustrated in Figure 12c. The conversion reaction of FeF_2_ was observed, with the amount of FeF_2_ gradually decreasing as the number of cycles increased. Importantly, the oxidation/reduction peaks at 1.5 and 2.3 V, shown in Figure 12d, indicate the gradual transformation of FeF_2_ into FeF_3_, ultimately stabilizing the reaction process as described in Equation (26).

Zheng et al.^[^ ^216^ ^]^ utilized FeF_3_ prepared by electrochemical degradation of Li_0.5_FeF_3_ as the cathode for sodium battery (Figure 12e). Initially, tetragonal FeF_3_ is transformed into tetragonal Na_x_FeF_3_, which then undergoes further phase transformations through the insertion and extraction of Na^+^, resulting in the formation of orthorhombic NaFeF3. As the number of cycles increases, a new transformation reaction is observed, notably between the cubic FeF_3_ and the tetragonal Na_x_FeF_3_, while tetragonal FeF_3_ continues to convert into tetragonal Na_x_FeF_3_. Additionally, the Li_0.5_FeF_3_ feedstock contains Li^+^, leading to the occurrence of side reactions involving Li^+^ during the process. The basic performance of the Na/tetragonal^−1^ FeF_3_ battery can be observed in Figure 12f. Currently, the mechanism of FeF_3_ is less studied and requires further investigation.

Compared to the complex reaction mechanism of FeF_3_, metal difluoride (MF_2_) exhibits a simpler mechanism, following a basic discharge reaction process as described by^[^ ^217^, ^218^ ^]^

(28) $$MF_{2}+2Na^{+}+2e^{-}⇌M+2\mathrm{NaF}$$

(29)

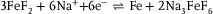

where M represents transition metals such as Fe, Co, Mn, Cu, etc., Fe is typically fully reversible. Notably, FeF_2_ has a band gap of 3.14 eV, which provides better cycle retention compared to other metal difluorides. Given the predominance of Fe‐based studies, FeF_2_ will be used as an example. Unlike other metal‐based compounds, FeF_2_ undergoes a disproportionation reaction (Equation 29) during the conversion process, producing ultrafine Fe nanocrystals ranging from 1 to 4 nm and Na_3_FeF_6_. The Fe nanoparticles can further create a bicontinuous framework of conductive networks, enhancing the battery's electrical conductivity.^[^ ^219^ ^]^ However, Na_3_FeF_6_ constitutes the majority of the discharge products. During charging, a portion of Na_3_FeF_6_ is converted back to FeF_3_, which is challenging to reactivate through subsequent redox processes, leading to insufficient cycling performance for the battery.^[^ ^220^ ^]^ Additionally, the particle size of FeF_2_ generated during charging and discharging is larger than that of the original FeF, which results in expansion and contraction of the active material and contributes to battery volume expansion issues.^[^ ^213^ ^]^

In contrast to FeF_2_, CuF_2_ undergoes (Equation 28) a one‐step conversion reaction and achieves a first discharge capacity of 502 mAh g^−1^, which is close to its theoretical specific capacity.^[^ ^56^ ^]^ The current process and fundamental properties of CuF_2_ composite cathode materials are illustrated in Figure 12g,h. However, during cycling, Cu tends to dissolve and form an irreversible coating on the electrode surface, leading to a decline in the reversibility of the CuF_2_ cathode. As a result, while the CuF_2_ cathodes show promising high specific capacity, further improvements are needed to enhance their performance.^[^ ^221^ ^]^

#### 3.3.2 Transition‐Metal Bromide, Metal‐Iodide Batteries

Transition metal bromides and iodides have emerged as promising candidates for the next generation of mass‐producible SIBs due to their high reversible redox properties, high energy density, and abundance. Furthermore, in light of the high temperature working environment and low safety associated with ZEBRA batteries, the study of metal halides has expanded from chloride(Cl^−^) to t bromide (Br^−^) and iodide (I^−^) compounds.^[^ ^222^ ^]^ However, challenges such as thermodynamic instability and the shuttle effect significantly hinder their development.^[^ ^223^ ^]^ Consequently, the exploration of SIBs utilizing transition metal bromides and iodides as cathodes has been limited, resulting in only a preliminary understanding from existing studies.

(30) $$\mathrm{Ni}l_{2}+2Na^{+}+2e^{-}⇌\mathrm{Ni}+2\mathrm{NaI}$$

**Figure 13:**
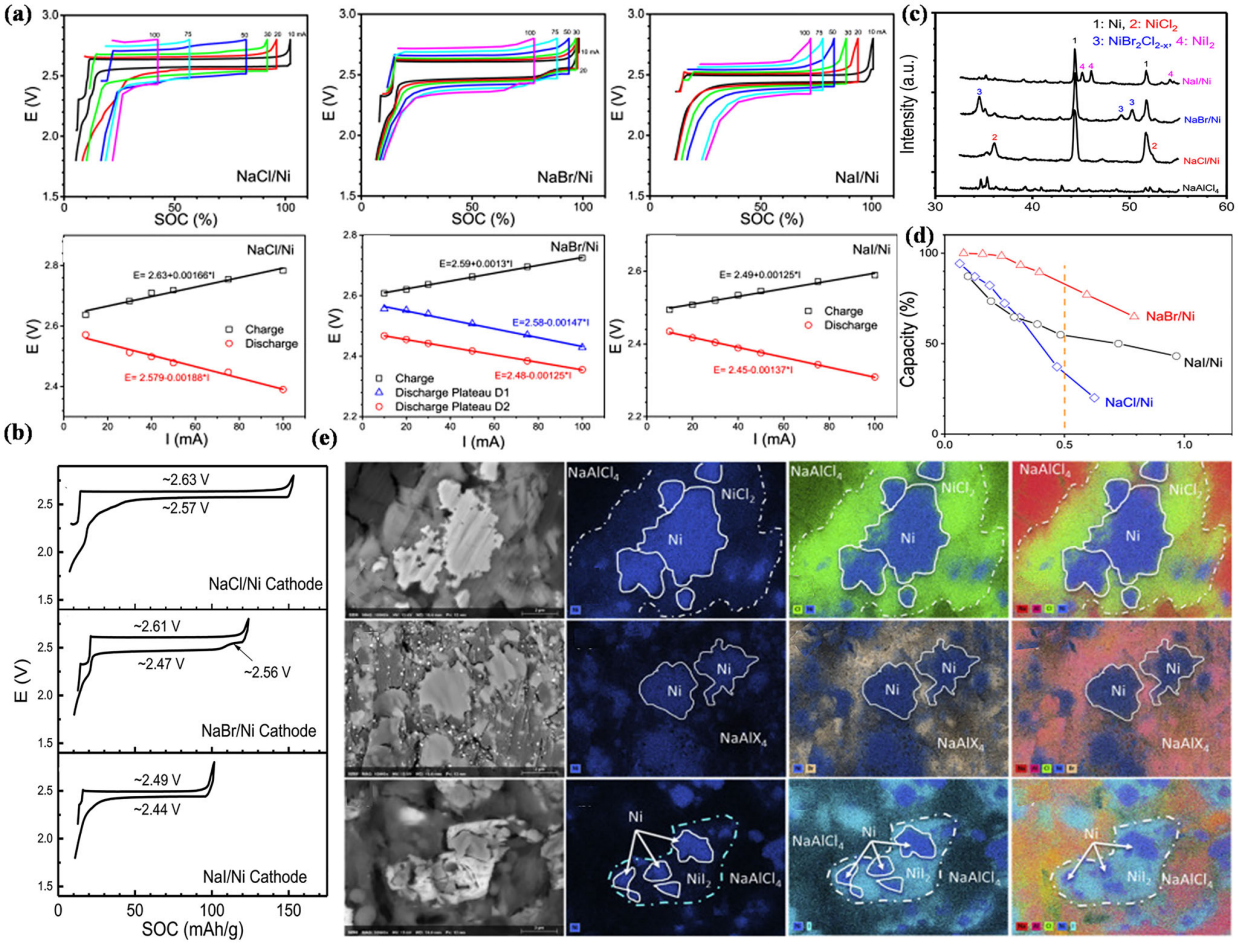
Electrochemical properties of NaCl/Ni, NaBr/Ni, and NaI/Ni cathodes. a) Voltage distribution diagram and relationship between voltage and current; b) Typical voltage profile; c) XRD profile at 100% state of charge; d) Capacity retention rate versus C rate; e) SEM image and EDX elemental mapping of the cathode extracted from the battery. Reproduced with permission.^[^ ^186^ ^]^ Copyright 2020, Elsevier.

The Na‐NiI_2_ battery undergoes a one‐step conversion reaction at 190 °C and 2.46 V, while the Na‐NiBr_2_ battery experiences an extremely complex reaction process. As shown in the voltage distribution plots and XRD patterns of the three types of batteries—Na‐NiCl_2_, Na‐NiBr_2_, and Na‐NiI_2_ (**Figure** **13**a–c)—the single‐platform characteristics of the Na‐NiI_2_ and Na‐NiCl_2_ batteries confirm the one‐step conversion process during the conversion reaction of the NaI/Ni and NaCl/Ni batteries, resulting in the production of NiI_2_ and NiCl_2_ during the charging reaction.^[^ ^224^ ^]^ In contrast, the NaBr/Ni battery exhibits two discharge platforms and a stepwise discharge process due to the formation of multiple NaBr_1‐x_Cl_x_ phases (where x = 0.1, 0.2, 0.3, 0.4) during the discharge process, allowing the battery to demonstrate improved multiplicative performance (Figure 13d).^[^ ^186^ ^]^ Unfortunately, Br^−^ participates in side reactions with the electrolyte (AlCl_4_^−^). Even more concerning is that the end product of the battery during charging is not NiBr_2_ as traditionally theorized, but rather NiBr_2‐x_Cl_2x_ (Figure 13e).^[^ ^185^ ^]^ Additionally, Zhu et al.^[^ ^225^ ^]^ proposed a computational model to predict the performance of Na/CuI_2_ batteries at the laboratory scale using the Nernst–Planck–Poisson (NPP) formula based on the solution of the transient conservation approach. The charge–discharge reaction process of CuI_2_ as a cathode is suggested to be analogous to that of the NiI_2_ cathode.

In general, the reaction mechanism of sodium‐metal halide (Na‐MH) batteries is relatively well‐understood, facilitating further research on transition metal halide cathodes. Due to the diverse range of transition metal halides available, they hold significant promise for large‐scale development. However, substantial variations in their reaction pathways present research challenges. Among these halides, fluorides and chlorides have advanced more rapidly, while bromides and iodides remain in the early stages of investigation. Notably, the Na‐NiBr_2_ battery exhibits the most complex reaction mechanism. Given the above mechanism, Na‐MH batteries demonstrate high theoretical specific capacity, good rate performance, long cycle stability, and high safety. **Table** **4** compiles recent progress in modifying conversion‐type cathodes for Na‐MH batteries. Despite research beginning decades ago, progress has been slow, indicating a long road ahead for future development. Advancing this field requires a deeper exploration of the fundamental mechanisms governing metal halides in sodium batteries and their practical application. Such research is crucial for achieving practical capacities approaching theoretical limits, thereby enabling real‐world implementation. This progress will bring the realization of practical high‐capacity batteries closer to fruition and pave a promising path for the development of low‐conductivity, high‐capacity electrode materials.

**Table 4:** Performance comparison of cathode materials in Na‐MH batteries.

Types of materials	Morphology	Current density (mA g^−1^)	Initial capacity (mAh g^−1^)	Cycle number	Capacity after circulation (mAh g^−1^)	Decay per cycle (%)	Capacity retention (%)	Refs.
NiCl_2_‐rGO	3D hierarchical	—	457.5	50	116.6	1.290	25.5	[187]
Fe/NaCl	Particle	10 (mA cm^−1^)	298.0	200	295.00	0.015	99.0	[200]
FeF_3_/graphene	Sheet‐like structure	0.3 C	344	50	115.80	1.327	33.7	[212]
FeF_2_‐rGO‐PAA	Nanocomposite	200	135.0	200	120.00	0.056	88.9	[213]
FeF_3_ – Fe – RGO	Sheet structure	100	130.0	1000	70.00	0.046	53.8	[215]
FeF_2_@NGC	Spherical nanoparticle	300	271.0	500	214.20	0.042	79.0	[220]
CuF_2_‐KB	Nanocomposite particle	0.05 C	502.0	30	59.00	2.955	11.3	[56]
NCCNs	Nanofibrous architecture	67.6	126.0	100	102.06	0.190	81.0	[226]
FeF_3_·0.33H_2_O/AlPO_4_	Hierarchical mesoporous hollow structure	20	290.0	80	211	0.340	72.8	[227]
FeF_3_·0.33H_2_O@C	Pomegranate‐like structure	0.1 C	178.9	4	159.80	2.675	89.3	[228]
FeF_3_·0.33H_2_O@rGO	Hierarchical mesoporous	1 C	172.3	100	101.00	0.414	58.6	[229]
FeF_3_ · 0.33H_2_O@3D‐OMCs	Nanocomposite	60	183	100	125.00	0.317	68.3	[230]
FeF_3_·0.33H_2_O/MWCNTs	Mesoporous spherical	23.7	350.4	50	123.5	1.295	35.2	[231]
FeF_3_‐CNF	Network	20	230.0	100	161.00	0.300	70	[232]
FeF_3_·0.33H_2_O	Raspberry‐like hexagonal star‐shaped	0.1 C	193.0	100	150.00	0.222	77.7	[233]
FeF_2_@MHCS	Hollow carbon spheres	30	220.0	40	190.00	0.340	86.4	[234]
FeF_2_@GC	Nanoparticles wrap the carbon layer	50	611.4	300	120.50	0.268	19.7	[235]
FeF_3_·0.33H_2_O	Flower‐like mesoporous	0.1 C	283.0	100	190.00	0.329	67.1	[236]
FeF_3_⚫0.33H_2_O/C	Open mesoporous structure	1 C	276.4	50	193.50	0.600	70.0	[237]
FeF_3_/C	Highly‐graphitized porous branch‐like carbon framework	75	286.0	100	126.70	0.557	44.3	[238]

### 3.4 Reaction Mechanisms of Other Cathodes

In addition to the most representative S, O, and TMHs cathodes mentioned above, there are other metal compounds, such as Se, TMOs, and TMSs, which have been mentioned before, and relatively little research has been done on these conversion‐type cathodes. Se cathodes and S cathodes have similar conversion processes in sodium ion batteries, including transition metal selenides that have emerged in recent years, which are similar to the conversion reactions of TMOs and TMSs. However, TMOs may also have more complex intermediate phases that require further clarification. The conversion reactions of TMOs and TMSs are similar to those of TMHs. Therefore, this section categorizes the reaction mechanisms of Se, TMOs, and TMSs cathodes in sodium‐ion batteries as other conversion cathodes for detailed discussion. Selenides that have emerged in recent years are similar to the conversion reactions of TMOs and TMSs. However, TMOs may also have more complex intermediate phases that require further clarification. The conversion reactions of TMOs and TMSs are similar to those of TMHs. Therefore, this section categorizes the reaction mechanisms of Se, TMOs, and TMSs cathodes in sodium‐ion batteries as other conversion cathodes for detailed discussion.

Although the development of the Se cathode began much later than that of the S cathode, the electrochemical reaction mechanisms of the two are very similar. The Se cathode has a higher volumetric energy density and electrical conductivity.^[^ ^239^ ^]^ In general, Se has different molecular forms, including cyclic, chain‐like, and small‐molecule forms, which lead to different specific conversion reaction processes.^[^ ^131^ ^]^ The discharge process can be attributed to the following:^[^ ^240^, ^241^, ^242^ ^]^

(31)

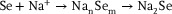




**Figure 14:**
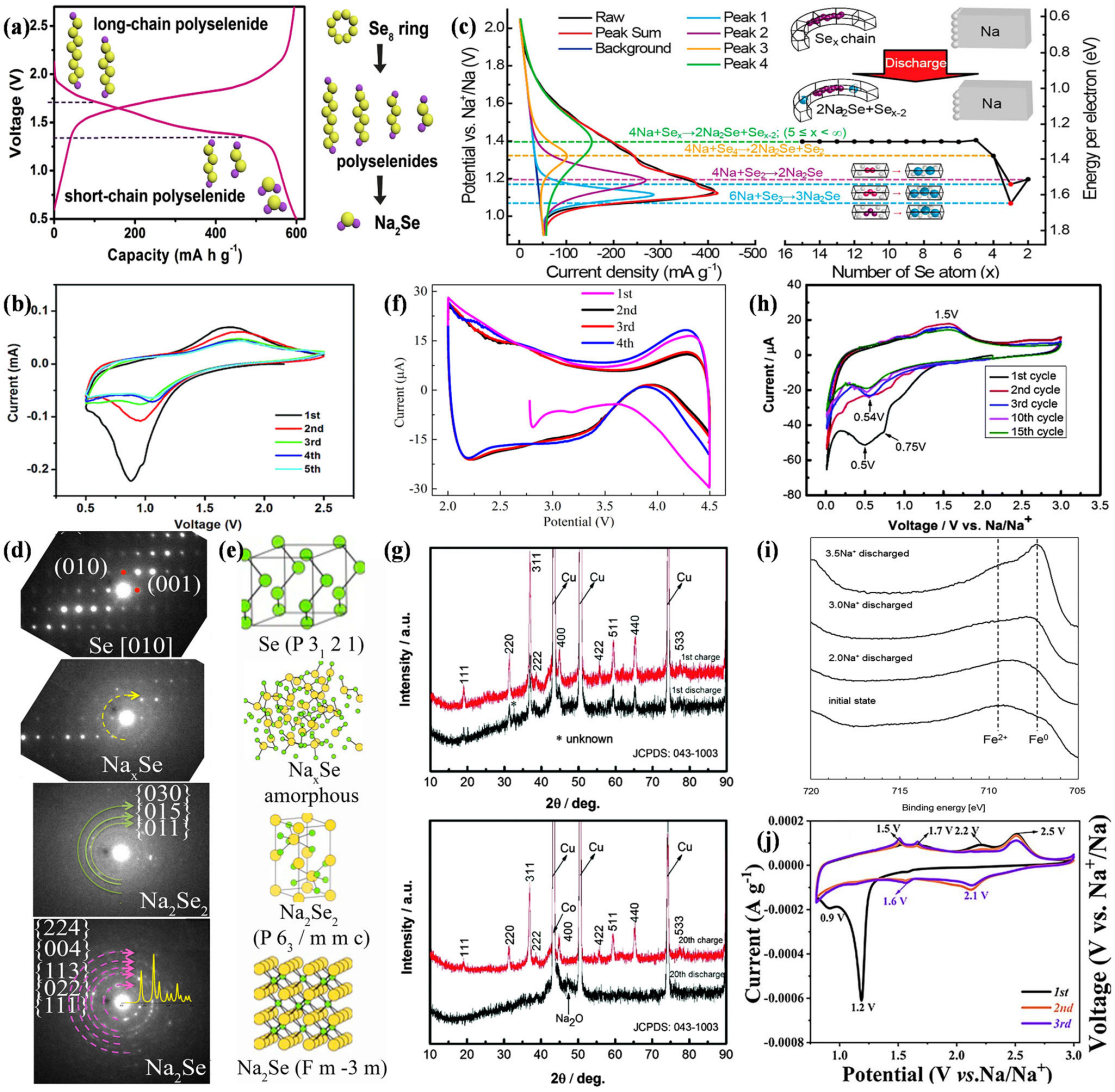
a) CV curves of a sodium ion battery with Se–C composite cathode at a scanning rate of 0.1 mV s ^−1^. Reproduced with permission.^[^ ^240^ ^]^ Copyright 2019, Elsevier. b) Cyclic voltammograms of the CPAN/Se fiber electrodes in Na–Se batteries at a scan rate of 0.1 mV s^−1^. Reproduced with permission.^[^ ^241^ ^]^ Copyright 2014, Royal Society of Chemistry. c) Electrochemistry of a Na–Se_x_ battery in Cycle 1. Reproduced with permission.^[^ ^242^ ^]^ Copyright 2016, American Chemical Society. d) The evolution of electron diffraction patterns in the sodiation process. And e) the illustration of the atomic structures of Se, amorphous Na_x_Se, crystalline Na_2_Se_2,_ and Na_2_Se phases appeared in the sodiation process. Reproduced with permission.^[^ ^244^ ^]^ Copyright 2016, American Chemical Society. f) CV curves of α‐Fe_2_O_3_ nanoceramics at a scanning rate of 0.1 mV s^−1^. Reproduced under the terms of the CC‐BY 4.0 license.^[^ ^247^ ^]^ Copyright 2021, Hanqing Dai et al. g) Ex situ XRD profile of Co_3_O_4_ electrode in full charge and discharge state during the first and 20th cycles. And h) the cyclic voltammogram recorded of Co_3_O_4_ electrodes at a scan rate between 0.01 and 3.0 V. Reproduced under the terms of the CC‐BY 3.0 license.^[^ ^250^ ^]^ Copyright 2014, Md Mokhlesur Rahman et al. i) XPS spectra of FeS_2_ during the first discharge/charge cycle. Reproduced with permission.^[^ ^251^ ^]^ Copyright 2014, Elsevier. j) CV curves of C@Fe_1−x_S/FeS_2_ composite in SIBs for the initial three cycles at 0.1 mV s^−1^. Reproduced with permission.^[^ ^254^ ^]^ Copyright 2024, Elsevier.

Among them, n is between 0.5 and 2, and m is between 1 and 8. In the Na–Se battery, the binding of Se and Na^+^ ions produces the intermediate product sodium polyselenide, which eventually forms the discharge product sodium selenide (Na_2_Se). Similarly, the inherent low electronic conductivity and shuttability of the higher‐order polyselenides (such as Na_2_Se_4_, Na_2_Se_6_, and Na_2_Se_8_) produced in the intermediate process lead to their poor electrochemical performance.^[^ ^23^ ^]^ For cyclic Se_8_, long‐chain polyselenides (Na_2_Se_n_, 4≤n≤8) will first form on a 1.7 V platform, followed by the formation of short‐chain polyselenides at ≈1.3 V. Upon further discharge to 0.5 V, Se is completely converted to solid Na_2_Se, as shown in **Figure** **14**a.^[^ ^240^ ^]^ Another is that Se is embedded in the form of small molecules in the matrix as the cathode. In the typical CV of the Na–Se battery (shown in Figure 14b), the Na–Se battery only has a pair of reversible redox peaks, indicating that the Sex chain undergoes a single‐phase transformation reaction to form insoluble Na_2_Se. During this period, no soluble intermediate phase Li_2_Se_x_ (x ≥ 4) is formed, which is consistent with previous studies on Se cathodes.^[^ ^239^, ^243^ ^]^ The chain‐like Sen is more complicated. First, Sen is converted to Na_2_Se, and then partially oxidized to small molecules Se_n−2_,5≤n (e.g., Sn_4_). After five cycles, Sn_4_ is completely converted to Sn_2_, and finally, all of it forms Na_2_Se. The detailed process can be seen in Figure 14c.^[^ ^242^ ^]^ Another theory is that the chain‐like Se_n_ successively goes through the amorphous phases Na_0.5_Se, Na_2_Se_2_, and Na_2_Se. After sufficient sodiumation, all intermediate phases completely transition to Na_2_Se. Figure 14d,e shows the structural phase changes during the sodiumation process.^[^ ^244^ ^]^

The theoretical specific capacity of TMOs cathodes is between 600–1300 mA h g^−1^, with the advantages of being high security, resource‐rich, and environmentally friendly.^[^ ^245^ ^]^ However, pure TMOs have the problems of low conductivity and electrode pulverization, resulting in a lower actual specific capacity and thus affecting the cycle life.^[^ ^246^ ^]^ Therefore, there has been little research on TMOs as cathodes. Taking Fe_2_O_3_ as an example, the conversion reaction can be expressed as:^[^ ^247^ ^]^

(32) $$Fe_{2}O_{3}+6Na^{+}+6e^{-}⇌2Fe+3Na_{2}O$$

The conversion reaction of the sodium‐metal iron oxide battery occurs between 2 and 4.5 V. After discharge, metal Fe nanoparticles with high electrical conductivity and electrochemically active Na_2_O are produced.^[^ ^248^ ^]^ During the subsequent charging process, Fe and Na_2_O gradually change until the end of the conversion reaction, when most Fe and Na_2_O are converted to Fe_2_O_3_. Therefore, the α‐Fe_2_O_3_ nanoceramics are highly reversible during the charging and discharging process, as can be seen in Figure 14f. With repeated cycles, the sodium‐metal iron oxide battery undergoes a pseudocapacitive reaction, which causes slight changes in the electrochemical activity of the particle surface and thus affects the reaction.^[^ ^249^ ^]^ In theory, Co_3_O_4_ also has the same conversion reaction process. And the in situ XRD test of the electrode material after the cycle confirmed the existence of the Co_3_O_4_ phase and the disappearance of Na_2_O (Figure 14g). At the same time, it can be seen in Figure 14h that the conversion reaction of nanostructured Co_3_O_4_ is reversible in the voltage range of 0.01–3.0 V.^[^ ^250^ ^]^

Although the development of TMSs cathodes began almost simultaneously with that of TMOs, there have been more studies on TMSs as cathodes in sodium‐ion batteries. This may be because the presence of the S element makes sodium metal sulfide batteries potentially have better electrochemical properties than Na–S batteries.^[^ ^251^ ^]^ However, problems such as the dissolution of polysulfides and pulverization of the electrode are also faced, resulting in a decline in specific capacity and poor rate performance.^[^ ^252^ ^]^Among sodium metal sulfide batteries, Fe‐based sulfides have been studied more than Cu‐ and Co‐based ones, and the conversion reaction mechanism of FeS_2_ as a cathode in sodium ion batteries is clearer.^[^ ^253^, ^254^ ^]^ Therefore, FeS_2_ is used as a representative of TMSs cathodes to analyze the conversion reaction process.

(33) $$FeS_{2}+xNa^{+}+xe^{-}⇌Na_{x}FeS_{2}\;x\leq 2$$

(34) $$Na_{x}FeS_{2}+\left(4-x\right)Na^{+}\left(4-x\right)e^{-}⇌2Na_{2}S+Fe$$

In a NaFeS_2_ battery, the conversion reaction at the FeS_2_ cathode satisfies Equations (33) and (34), and FeS_2_ will produce Na_2_S and Fe metal nanoparticles through a complex intermediate phase.^[^ ^255^ ^]^ As early as 2008, Kim et al. first proposed that FeS_2_ in the cathode would produce sodium sulfide and iron during discharge, but did not provide a clear explanation for the severe tilting of the second discharge curve.^[^ ^256^ ^]^ It was not until six years later that the Kitajou team used XPS spectroscopy to observe the first charge and discharge of the FeS2 cathode (as shown in Figure 14i), and deduced the conversion reaction of the sodium iron sulfide battery based on the conversion reaction of the lithium metal iron sulfide battery (i.e., when x = 2). In the same year, it was proposed that the intermediate product was a new NaFeS_2_ phase.^[^ ^257^ ^]^ And Zhu et al. proposed that after the first charge and discharge, the conversion reaction produced irreversible FeS_2_, which caused a difference with the subsequent charge and discharge curves.^[^ ^258^ ^]^ Subsequently, it was also calculated using first‐principles calculations that the cathode would produce the intermediate product NaxFeS_2_ (x = 1.5). Recently, Huang and his colleagues proposed a more complex conversion reaction process. As can be seen in Figure 14j, different peaks appear at different voltage positions, representing different conversion reaction processes, but the value of x needs to be further confirmed.^[^ ^254^ ^]^ Therefore, the conversion reaction of the above equation occurs in the sodium metal iron sulfide battery between 0.6 and 3V, but the detailed intermediate products are not clear. In the future, the reaction mechanism and intermediate product structure of the TMSs cathode will require in‐depth thinking and research.

In general, the conversion reactions of other conversion‐type cathode materials in sodium‐ion batteries are not yet clear, and in‐depth research in this area is lacking. Based on the existing mechanism, these sodium‐ion batteries have the advantages of high specific capacity and high abundance. **Table** **5** summarizes contemporary engineering strategies applied to conversion‐type cathodes in Na‐MH batteries. In the future, a more in‐depth exploration of the mechanism and application of conversion‐type cathode materials such as Se, TMOs and TMSs that are waiting to be developed in sodium batteries will help to promote the development process of conversion‐type cathode materials, thereby improving the overall specific capacity of the battery and becoming the most powerful reserve material for sodium batteries that achieve long cycle and high rate performance.

**Table 5:** Performance comparison of other conversion‐type cathode materials in Na‐ion batteries.

Types of materials	Morphology	Current density (mA g^−1^)	Initial capacity (mAh g^−1^)	Cycle number	Capacity after circulation (mAh g^−1^)	Refs.
Se–C	Carbon nanotube‐containing composite	50	600.00	50	265.00	[48]
Se_8_/C	Mesoporous carbon composites	0.25 C	485.00	380	340	[239]
CPAN/Se	Organic carbon skeleton	0.3C	615.00	300	410.00	[241]
Se@PCNFs	Porous carbon	0.05	595.00	80	520	[259]
C/Se	Carbon‐bonded and encapsulated selenium composites	100	605	50	258.00	[260]
Se@CNFs‐CNT	Flexible free‐standing material for 3D interconnected	0.5	515.00	240	410.00	[261]
Se/(CNT@MPC)	Microporous carbon	0.1	661.00	100	522.00	[242]
Se‐CCN	Carbon Nanosheet	0.2C	585.00	500	515.00	[262]
Se‐NCMC	Selenium into the pores of the membrane	0.1C	521.00	150	511.00	[263]
N‐doped Fe_3_C@C/Se	Porous adsorption material	0.1C	620	100	555.00	[264]
Se–C composite	Jackfruit‐like hollow structure	0.2C	1000.00	100	593.90	[240]
HCS@Se	Hollow carbon spheres	0.1 c	609	100	512.00	[30]
MoSe_2_/C	Coaxial‐cable composites	0.5	550.00	100	423.00	[265]
α‐Fe_2_O_3_	Nanoceramics	25	692.50	800	201.80	[247]
CoS_2_ /MWCNT	Nanocomposites	100	823.00	100	568	[266]
FeS@C	Yolkshell spheres	100	—	100	545.00	[267]
FeS_2_	Spherical shape	60	530.00	100	450.00	[258]
CuS	Flower‐like microspheres	31	348.6	100	41.80	[268]
FeS_2_@C nanorods	Porous sea cucumber‐like	20000	—	10000	160.00	[253]
U‐Cu_7_S_4_NPs@G	Unique interconnected graphene conductive networks	100	585.50	2000	409.90	[269]
FeS_2_‐C	Substrate‐free flexible carbon‐coated	0.5C	644.37	400	579.93	[270]
C@Fe_1−x_S/FeS_2_	Dragon fruit‐like morphology	500	—	500	370.40	[254]

In conclusion, while conversion‐type cathode materials exhibit diverse reaction pathways, they share a common underlying reaction mechanism. Specifically, sulfur (S) undergoes multistep conversion reactions forming polysulfides, oxygen (O) experiences a single‐step conversion reaction yielding distinct discharge products and transition metal halides, due to their variety, display unique conversion characteristics. Nevertheless, all can be fundamentally described as reversible reactions involving Na⁺ with the cathode material. Consequently, these cathode materials inherently offer the same key advantages: high theoretical specific capacity, abundant resources, and enhanced safety. These attributes will drive the transformation of SIBs toward higher efficiency and greater energy density. Analysis of the aforementioned mechanisms reveals areas where understanding remains incomplete. Persistent investigation and in‐depth analysis of the fundamental mechanisms are therefore essential for future progress. Furthermore, these cathodes face shared challenges, such as volume expansion, side reactions, and low practical specific capacity. Although mitigation strategies have been developed to date, ongoing theoretical research is needed to devise novel modification approaches to continuously address these issues. Leveraging the intrinsic advantages of conversion‐type cathode materials will accelerate the development journey of sodium‐ion batteries as next‐generation energy storage systems.

## 4 Challenges and Strategies of Conversion‐Type Cathodes

### 4.1 Significant Volume Expansion

Volume expansion of conversion‐type cathode material is a common phenomenon during the discharge process of sodium batteries and represents a significant issue in sodium battery research.^[^ ^271^ ^]^ This volume expansion primarily occurs due to the reduction of Na^+^ within the cathode material during discharge. Specifically, as the discharge process unfolds, the conversion‐type cathode experiences the migration of electrons and ions, leading to conversion reactions on the cathode side. This results in the deposition or solidification of discharge products either on the surface or within the cathode.^[^ ^272^ ^]^ Compared to the initial state, the size of the cathode changes significantly due to the volume changes of these products, ultimately causing volume expansion in the cathode.

From the perspective of object size, size effects become noticeable when an object's dimensions deviate from their original state, indicating a volume change. This can occur at various scales, from atoms to nanometers, and from nanometers to micrometers.^[^ ^273^ ^]^ It is also this size effect that allows for the investigation of new electrode charge storage mechanisms, such as conversion reactions.

**Figure 15:**
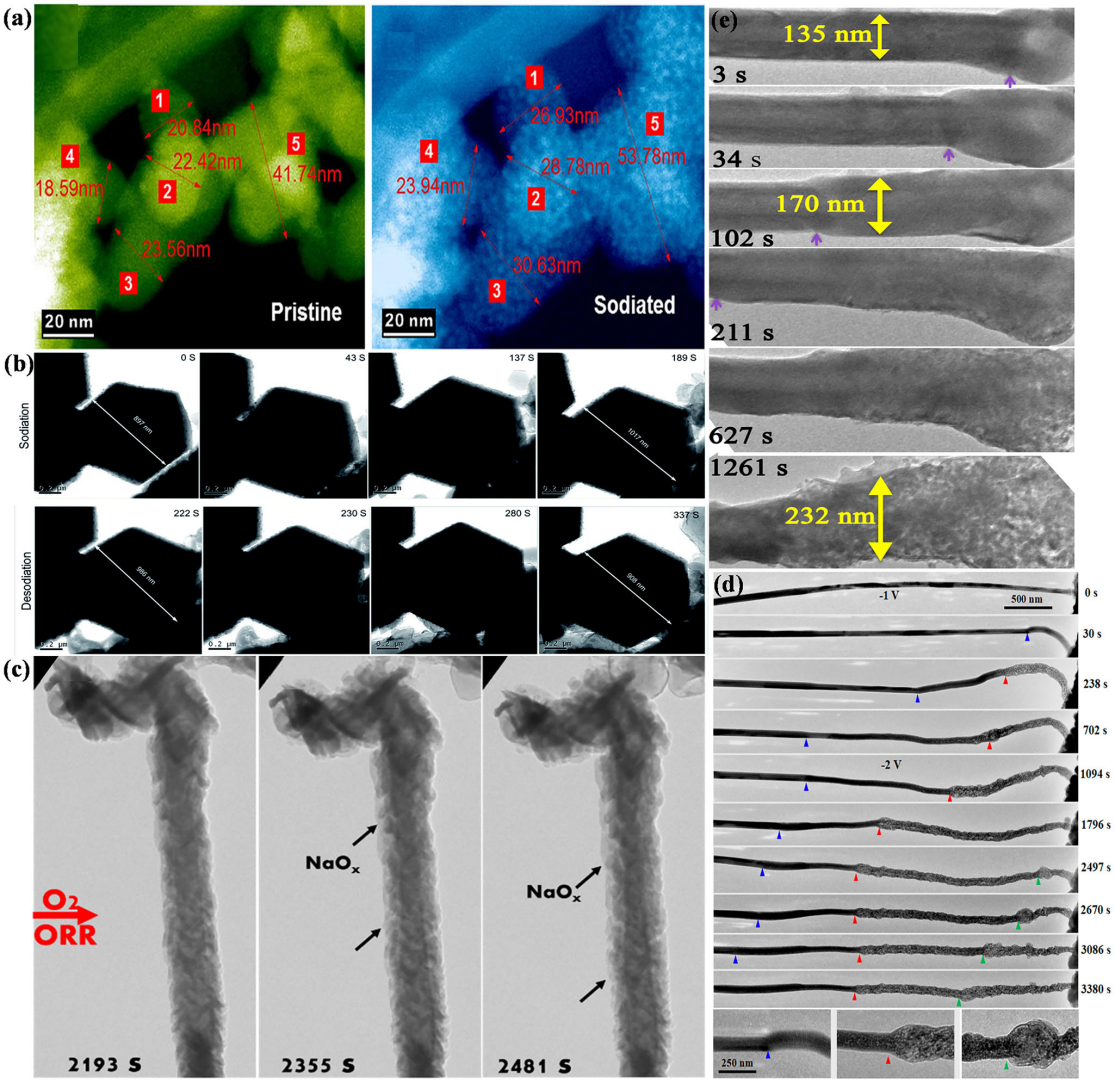
The image illustrates the volume change of the conversation‐type cathode. a) Comparison of size changes of raw and mediated FeF_2_ nanoparticles. Reproduced with permission.^[^ ^219^ ^]^ Copyright 2014, American Chemical Society. b) In situ TEM images of the Co_1_‐ZnS/C@S cathode before and after charging and discharging. Reproduced with permission.^[^ ^119^ ^]^ Copyright 2013, Royal Society of Chemistry. c) Na–O_2_ battery changes before and after discharge reaction. Reproduced with permission.^[^ ^278^ ^]^ Copyright 2020, American Chemical Society. d) Cubic volume change of the cathode structure when the CuO NW cathode is discharged in an oxygen environment. Reproduced with permission.^[^ ^279^ ^]^ Copyright 2018, American Chemical Society. e) The morphological evolution of individual selenium nanotubes during the sodiation process in a Na–Se battery. Reproduced with permission.^[^ ^244^ ^]^ Copyright 2016, American Chemical Society.

Compared with lithium batteries, sodium batteries experience more severe volume expansion,^[^ ^274^ ^]^ primarily because the sodium ion radius is 25–55% larger than that of lithium ions. Additionally, there are two reaction processes for the same substance in these battery types. For example, FeF_2_ serves as a cathode in lithium batteries and undergoes only a single step of conversion reaction. In contrast, the situation is more complex in sodium batteries, where FeF_2_ experiences a disproportionation reaction alongside the conversion reaction.^[^ ^275^ ^]^ He et al.^[^ ^219^ ^]^ demonstrated that Fe^2^⁺ transforms into Fe⁰ and Fe^3^⁺ during sodiation/desodiation, further exacerbating volume expansion (**Figure** **15**a).

#### 4.1.1 Manifestations of Volume Expansion

The volume expansion in conversion‐type cathodes is generally greater than that in intercalation‐type cathodes. This substantial volume expansion can adversely affect the physical and chemical performance of the battery. First, the cathode structure may become damaged, leading to structural degradation after repeated cycling. This degradation can compromise the integrity of the cathode structure and, in severe cases, result in electrode fracture. Such issues can negatively impact the battery's electrochemical performance, including cycling stability, reversible capacity loss, and slow kinetics, and may even pose safety risks. Therefore, effectively addressing the volume expansion of cathode materials during the charging and discharging processes has become a pressing challenge in the development of conversion‐type SIBs. The effects of the volume expansion phenomenon on various types of conversion‐type cathodes are outlined as follows:

Na–S battery: According to the currently available research literature, it is known that during the conversion reaction of the sulfur cathode, the complete precipitation to Na_2_S leads to a volume expansion of ≈170% in room‐temperature (RT) Na–S batteries.^[^ ^109^, ^276^ ^]^ This is significantly greater than the 80% volume change observed in lithium–sulfur batteries,^[^ ^277^ ^]^ which tends to result in more substantial pulverization of the cathode. During the cycling process of RT Na–S batteries, singlet sulfur undergoes a sequence of transformations: S → Na_2_S_8_ → Na_2_S_x_ (4 ≤ x ≤ 6) → Na_2_S_x_ (x = 2, 3) → Na_2_S. The mass density of singlet sulfur is approx ≈ 2.07 g cm^−3^, while the mass density of the final discharge product, sodium sulfide, decreases to 1.86 g cm^−3^. This 0.2 g cm^−3^ difference in density before and after cycling contributes to the volume expansion observed in RT Na–S batteries.^[^ ^117^ ^]^

Yan et al.^[^ ^118^ ^]^ observed the discharge process of S@CoP–Co/NCNHC and S@NCP using in situ transmission electron microscopy, which illustrated the volume change of the solid S@NCP cathode during a 10‐min discharge. It was clearly observed that the diameter of the S@NCP cathode increased from 204 to 247 nm, while the diameter of the S@CoP‐Co/NCNHC cathode expanded from 371 to 392 nm over 20 min of discharge. Liu et al.^[^ ^119^ ^]^ also documented the sodiation/desodiation of Co1‐ZnS/C@S using in situ transmission electron microscopy (Figure 15b), providing further evidence that the Na–S cell undergoes volume expansion.

Na–O_2_ battery: In Na–O_2_ batteries, the primary discharge product is sodium superoxide (NaO_2_), although it can sometimes be accompanied by a disproportionation reaction that generates sodium peroxide (Na_2_O_2_). There are instances where only NaO_2_ or Na_2_O_2_ is produced. The formation of these products, coupled with oxygen release during disproportionation reactions, leads to repeated volume expansion in Na–O_2_ batteries, with the expansion rate reaching up to 200%. This subsequently induces irregular crack formation on the cathode surface.

Han and his coworkers^[^ ^278^ ^]^ investigated the reaction process of Cu‐based air cathodes using in situ transmission electron microscopy. They found that the copper itself reacts to form a composite of copper clusters, causing a volume expansion of ≈67%. Upon the introduction of gaseous O_2_, the oxygen reduction reaction (ORR) produces additional NaO_x_ particle products, which further increase in size from the initial diameter of 360–543 nm in Figure 15c.

Similarly, Liu's group^[^ ^279^ ^]^ observed the redox process of Na–O_2_ batteries utilizing copper oxide nanowires (NWs) as air cathodes. The copper oxide nanowires underwent three consecutive large volume expansions, measuring 70%, 340%, and 580%, respectively. Transmission electron microscopy (TEM) images in Figure 15d reveal that the initially smooth surfaces of the CuO NWs gradually increase in size over time, with cracks, holes, and significant structural damage appearing.

Na‐MH battery: When transition‐metal halides are used as the cathode, the conversion reaction and the formation of discharge products on the cathode side lead to volume changes. Particularly, multiple conversions and phase transitions cause the volume to expand repeatedly, resulting in capacity decline until battery failure. Gao et al.^[^ ^226^ ^]^ observed nickel (Ni) and sodium chloride (NaCl) before and after cycling using scanning electron microscopy (S‐3400N or SU8220). The SEM images showed that the size of Ni increased from the original 100–500 nm to 1 µm after 50 cycles. Additionally, the particle size of NaCl grew from 1–4 µm to 20–30 µm, which proves that the volume expansion of the cathode of the Na‐NiCl_2_ battery can reach 200% after cycling.

Other batteries: In addition to the above batteries, other sodium‐ion batteries composed of a converted cathode also exhibit significant volume expansion. In Na–Se batteries, similar to S cathodes, the Se cathode undergoes a multistep conversion to Na_2_Se, generating various polyselenides and resulting in a significant volume expansion of 336%. Li et al.^[^ ^244^ ^]^ used in situ TEM to observe the sodiumation process of selenium nanotubes. As shown in Figure 15e, the volume of the selenium nanotubes changed three times, which is because the sodium–selenium battery undergoes a three‐step reaction. The first step of volume expansion was 58%, from an initial 135 nm selenium nanotube to 170 nm. The expansion rate at this stage was faster than the physical and chemical processes. Subsequently, small crystalline nanoparticles formed on the surface of the selenium nanotubes, causing the diameter to expand to 282 nm. In addition, Ma et al.^[^ ^280^ ^]^ have also investigated the volume expansion issue in Na–Se batteries, demonstrating that Se undergoes ≈400% volume expansion during cycling. In contrast, Li–Se batteries exhibit a volume expansion rate of 180%, indicating that the volume change problem is particularly severe in Na–Se batteries.^[^ ^281^ ^]^ Furthermore, unfortunately, although TMOs and TMSs were investigated relatively early, studies on their volume expansion remain scarce and lack specific numerical data.

#### 4.1.2 Strategies Toward Stable Cathode

It is important to note that using S, O_2_, MH, and other conversion‐type materials directly as cathodes can limit the reaction kinetics of the battery due to the inherent insulating properties of these materials. This limitation exacerbates the uncontrolled formation of by‐products, leading to a deterioration in the battery's cycle life. To enhance the electrochemical performance of batteries, most studies typically opt to use cathode carriers to prepare composite conversion‐type cathodes. These carriers improve the electrical conductivity of the cathode material while providing a more stable environment and acting as a barrier against by‐products. Velinkar et al. investigated the influence of carbon cathodes on discharge products in sodium–oxygen (Na–O_2_) batteries, demonstrating that the discharge–charge energetics and deactivation are critically governed by the carbon cathode material.^[^ ^282^ ^]^ Currently, the most commonly studied cathode carriers include materials such as carbon, graphene, and acetylene black. The following section outlines the strategies for mitigating volume expansion in composite conversion‐type cathodes, which can be categorized into three main types: modulation of cathode carrier structure, construction of multifunctional nanocomposite cathode carriers, and optimization of cathode carrier surface activity.

##### Structural Modifications

Increasing the specific surface area of the conversion‐type cathode by modulating the structure of the cathode carrier can enhance its ability to adapt to the volume changes of discharge products, thereby maintaining structural integrity. Specifically, certain solvents are often used to etch the surface of the cathode carrier or to synthesize carriers with a large specific surface area using various substances. This process creates rough surfaces characterized by features such as holes and gaps. These structural modifications provide additional space for storing discharge products, which can help restrict their growth and alleviate volume expansion in the cathode. For instance, Sungjemmenla et al. demonstrated that the microporous carbon‐based sulfur host obtained through etching with hydrochloric acid exhibited desirable electrochemical performance.^[^ ^120^ ^]^ Common cathode carrier structures include porous or hollow frameworks and specially designed structures.

##### Porous or Hollow Structure Design

One of the most common approaches to structural control of cathode carriers is the use of porous or hollow structures. This design extends the cycle life of the cathode by forming pores on the outer surface and cavities inside, which buffer the stress caused by volume expansion, both spatially and mechanically.^[^ ^122^ ^]^ During the structural modulation process, chemical or physical methods are employed to transform the cathode surface from smooth to rough, creating holes or interstices on the surface. The micropores facilitate the carrying of reactants, while mesopores or macropores allow the electrolyte to infiltrate, enabling the conversion reaction to occur within the micropores. This configuration confines the discharge of products within the interstices, restraining the size effect of these products and further alleviating volume expansion through the hollow structure.

**Figure 16:**
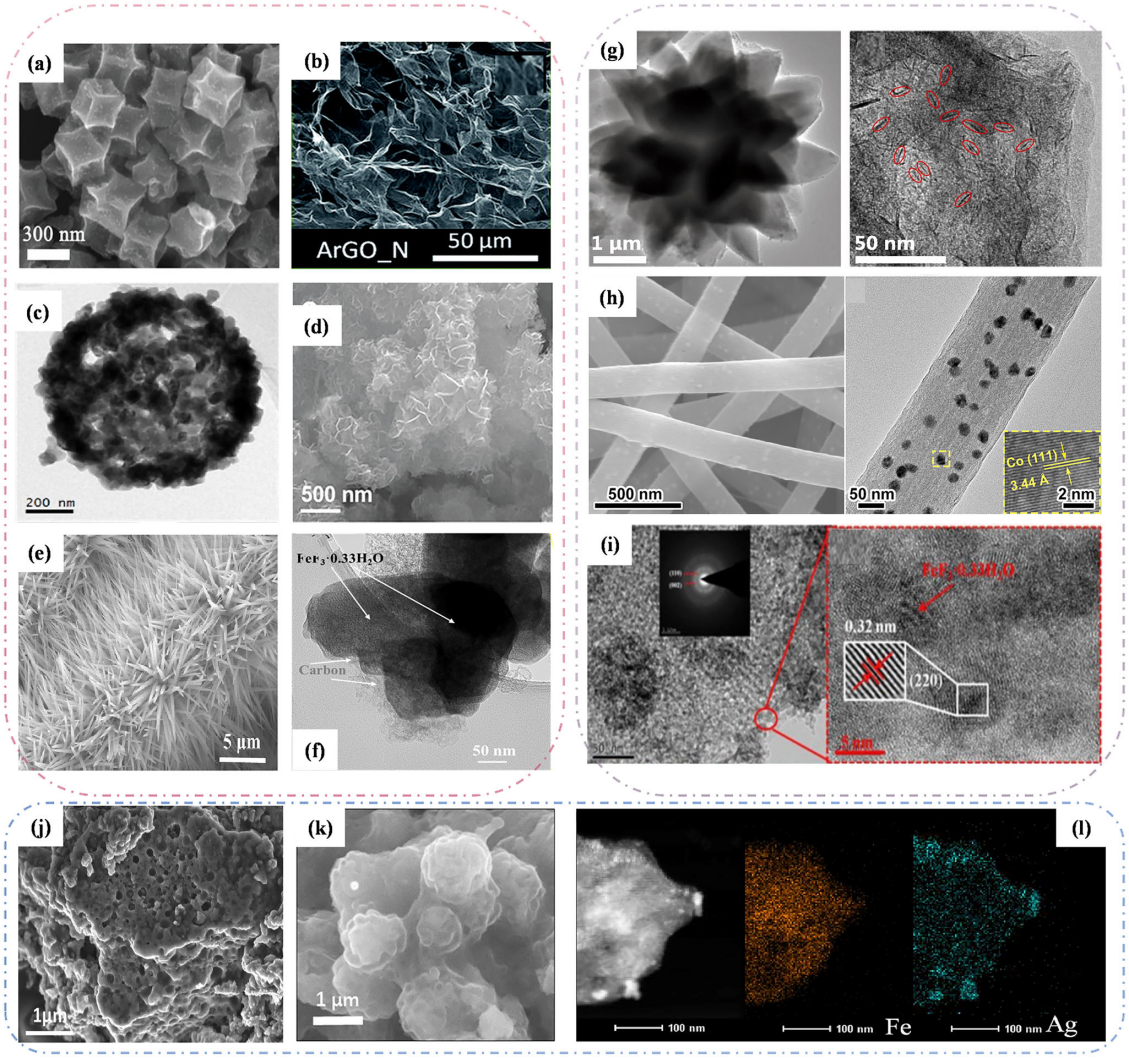
Conversion‐type cathode side volume expansion solution. a) SEM images of the S@HPC/Mo_2_C. Reproduced with permission.^[^ ^123^ ^]^ Copyright 2022, John Wiley and Sons. b) SEM images of the ArGO_N. Reproduced under the terms of the CC BY‐NC 3.0 license.^[^ ^135^ ^]^ Copyright 2018, Marina Enterría et al. c) HRTEM image of FeF_3_⚫0.33H_2_O.Reproduced with permission.^[^ ^227^ ^]^Copyright 2018, Elsevier. d) SEM images of the MoTe_2_. Reproduced with permission.^[^ ^124^ ^]^Copyright 2023, John Wiley and Sons. e) SEM image of C@NiCo_2_O_4_‐NAs. Reproduced with permission.^[^ ^176^ ^]^ Copyright 2019, Elsevier. f) High power TEM image of FeF_3_⚫0.33H_2_O@C nanocomposites. Reproduced with permission.^[^ ^228^ ^]^ Copyright 2022, Elsevier. g) TEM images of NOC@MoS_2_ composites. Reproduced with permission.^[^ ^125^ ^]^ Copyright 2024, Elsevier. h) SEM and TEM images of Co‐ECNCFs. Reproduced with permission.^[^ ^177^ ^]^Copyright 2017, Elsevier. i) TEM images and HRTEM images of FeF_3_⚫0.33H_2_O @3D‐OMCs. Reproduced under the terms of the Creative Commons Attribution 4.0 License.^[^ ^230^ ^]^ Copyright 2018, Rui Zhang et al. j) SEM image of Ni@NPC/S in RT Na–S battery. Reproduced with permission.^[^ ^127^ ^]^ Copyright 2023, Elsevier. k) SEM image after discharge of the PCS electrode. Reproduced with permission.^[^ ^178^ ^]^ Copyright 2017, John Wiley and Sons. l) TEM images and corresponding element mapping maps of Fe and Ag in the selected region of FeF2‐RGO composites after 50 charge and discharge cycles. Reproduced with permission.^[^ ^215^ ^]^ Copyright 2014, Elsevier.

For example, Zhou's group^[^ ^123^ ^]^ utilized a core–shell structure prepared from ZIF‐8 and ZIF‐67, along with (NH_4_)_6_Mo_7_O_24_⚫4H_2_O, to obtain heat‐treated HPC. They then created an S@HPC/Mo_2_C composite cathode via the melt diffusion method for use in Na‐S batteries. As shown in **Figure** **16**a, scanning electron microscopy (SEM) images of HPC/Mo_2_C reveal the presence of micropores (mainly concentrated at 0.52, 0.85, and 1.5 nm), mesopores (centered at 3.38 nm), and a hollow structure characterized by homogeneous polyhedral particles between 300 and 400 nm. The slight shrinkage observed on the surface is attributed to the growth of cobalt‐catalyzed carbon nanotubes during carbonization, while the porous structure plays a crucial role in determining the form of sulfur, directly impacting electrochemical performance. Even when sulfur reacts with the electrolyte, the cavity morphology remains intact, thereby mitigating issues related to volume changes.

Enterría and colleagues^[^ ^135^ ^]^ employed a simple Hummers' method to oxidize graphene suspensions, preparing graphene aerogels of rGO (room temperature), ArGO_N (−195 °C), and ArGO_U (−7 °C) under different temperature conditions as cathode carriers for Na‐O2 batteries. Their investigation revealed that the graphene aerogels featured wide mesopore sizes (5–25 nm) and narrow macropores (1200 nm) interconnected with each other, as observed through scanning electron microscopy (SEM) imaging in Figure 16b. The narrow pores were formed due to the creation of small and randomly oriented aggregates when the graphene oxide suspension was rapidly frozen. Among the three substances, ArGO_N exhibited the largest actual density, achieving a significant pore volume, which effectively accommodates the generated NaO_2_ particles and mitigates volume expansion.

Liu's team^[^ ^227^ ^]^ prepared hollow porous FeF_3_ centroid 0.33H_(2)_O microspheres via a solvothermal method, using non‐hydrated ferric nitrate (Fe(NO_3_)_3_⚫9H_2_O, Aldrich) as the iron source and hydrogen fluoride (HF) as the fluorine source (Figure 16c). They modified the structure with AlPO_4_ to produce HTB‐structured FeF_3_⚫0.33H_2_O, characterized by unique tunneling and serving as the cathode for Na‐MH batteries. The FeF_3_·0.33H_2_O presents hollow microspheres ≈1 µm in size, utilizing a porous shell and internal voids to facilitate electrolyte penetration into the inner part of the electrode while limiting the volume change during the sodiation process.

##### Special Structure Design

Beyond simple porous or hollow structures, cathode carriers can also be engineered with special structures, enabling the synthesis of cathode carriers with multiple configurations from various substances. This design advantageously increases the specific surface area, providing more space for accommodating discharge products and thus alleviating volume expansion. Researchers typically modulate cathode carriers into common shapes found in nature,^[^ ^283^ ^]^ such as flower‐like, egg‐like, sourdough‐like, and pomegranate‐like forms. Among these, the flower‐like design is the most prevalent due to its maximization of specific surface area, with the petals creating additional voids that buffer volume expansion by accommodating discharge products.

For example, Gao's group^[^ ^124^ ^]^ employed a solvothermal reaction to prepare 3D flower‐like molybdenum telluride nanorods composed of numerous nanosheets. SEM observations of the MoTe_2_ (Figure 16d) revealed that the abundance of MoTe_2_ nanosheets served as a physical barrier to mitigate volume changes of sulfur during the sodiation process. MoTe_2_ features a mixture of mesopores and macropores, which provide sufficient space to limit the volume expansion of sulfur.

Liu and his colleagues^[^ ^176^ ^]^ synthesized C@NiCo_2_O_4_‐NAs with a sea urchin structure as an O_2_ cathodic carrier using the hydrothermal method in conjunction with carbon paper. Observations of the morphology and microstructure of both carbon paper and C@NiCo_2_O_4_‐NAs (Figure 16e) showed that the porous carbon paper, with its 3D interwoven structure inside the sea urchin, provided a transport path for electrons and oxygen. The vertically aligned NiCo_2_O_4_ nanoneedles on the exterior created additional open space to accommodate discharge products, thereby inhibiting partial volume expansion and alleviating cathode passivation.

Zhang et al.^[^ ^228^ ^]^ prepared FeF_3_⚫0.33H_2_O@C cathode materials with a pomegranate structure through hydrothermal synthesis and fluorination, utilizing ferrocene as the iron source, acetone as the carbon source, and ammonium fluoride (NH_4_F) as the fluorine source. TEM and SEM images of FeF_3_⚫0.33H_2_O@C in Figure 16f demonstrated that FeF_3_⚫0.33H_2_O nanoparticles were not only uniformly distributed throughout the composite but were also tightly encapsulated. The presence of H_2_O as a filler within the large hexagonal cavity of the FeF_3_⚫0.33H_2_O crystals facilitates the insertion/extraction of large‐radius sodium ions, providing ample surface area to alleviate volume expansion.

Wang's team^[^ ^267^ ^]^ prepared FeS@C nanospheres with a yolk–shell structure. The nanospheres consist of single‐crystal FeS yolks with an average size of 170 nm and a porous carbon shell with a thickness of 30 nm. And the specially manufactured 20 nm gap between the yolk and the carbon shell can withstand the large volume change of FeS. Finally, it is calculated that the volume change of Na‐FeS@C is 170%, and the 16.5 nm gap space is sufficient to accommodate the volume expansion of the FeS yolk. This method allows the electrode to maintain its original shape without sacrificing specific capacity.

##### Construction of Multifunctional Nanocomposites

The preparation of nanocomposites using advanced nanotechnology enhances the capacity of cathode carriers to accommodate discharge products by transforming them into nanoscale materials. This development addresses the limitations of traditional cathode carriers, which often have fixed and restricted spaces, thus improving their overall performance. Nanotechnology enables the fabrication of nanofiber networks or frameworks using various materials, transforming rough surfaces into linear fibrous structures. This modification significantly increases the surface area available for product distribution within the cathode current collector. Furthermore, the elasticity of the nanofiber network acts like an “elastic bandage,” buffering stress from discharge product formation and enabling the system to effectively withstand repeated volume changes. Common methods for producing these nanofiber networks include electrospinning, frequently applied to materials like carbon. Recently, research focus has shifted toward utilizing Metal–Organic Frameworks (MOFs) and Covalent Organic Frameworks (COFs) to create composite materials that efficiently leverage 3D frameworks. These structures provide greater space to accommodate volume changes. Additionally, metallic and non‐metallic elements are being incorporated into the cathode current collector to enhance its functionality.

For example, Wu's group^[^ ^125^ ^]^ synthesized N, O co‐doped flower‐like porous carbon carriers (NOC) from Ni‐MOF precursors, then coated them with MoS_2_ nanosheets to form NOC@MoS_2_ composites. TEM characterization of NOC@MoS_2_ (Figure 16g) revealed that the nanosheets were stable and uniformly dispersed on the carbon surface. The MOF framework significantly increased the surface area and provided sufficient space for the neighboring nanosheets, allowing for a higher sulfur loading while limiting volume changes during the (de)sodiumation process.

Ma and his coworkers^[^ ^177^ ^]^ employed electrostatic spinning to produce binder‐free n‐doped carbon fibers (Co‐ECNCFs) for O_2_ cathodes. Transmission electron microscopy (Figure 16h) showed that the smooth, fibrous surface of Co‐ECNCFs interlaced to form a dense network structure. Co particles were observed to be anchored or embedded within these fibers, which enabled NaO_2_ to form a film‐like product at the Co particle locations, distributing it uniformly. The elasticity of the surrounding nanofibers alleviated the pressure exerted by volume changes, further mitigating volume expansion.

Zhang's team^[^ ^230^ ^]^ developed FeF_3_⚫0.33H_2_O@3D‐OMCs nanocomposites as the cathode of Na‐HM batteries through a nanocasting technique. The TEM and HRTEM images (Figure 16i) demonstrated that the FeF⚫0.33H_2_O@3D‐OMCs nanocomposites were perfectly encapsulated within a three‐dimensionally ordered mesoporous carbon (3D‐OMCs) matrix. This nanofiber network structure not only promoted the penetration of electrolyte ions but also suppressed the volume changes of discharge products, maintaining the structural stability of the FeF_3_⚫0.33H_2_O@3DOMCs nanocomposite and minimizing volume variations during Na^+^ insertion/degassing.

Wang et al.^[^ ^269^ ^]^ Synthesized U‐Cu_7_S_4_NPs@G as the cathode of the sib by a simple method of anchoring ultrafine Cu_7_S_4_ nanoparticles on graphene nanosheets (U‐Cu_7_S_4_NPs@G). This generated U‐Cu_7_S_4_NPs@G nanocomposite forms covalent bonds with S, and when Na+ is inserted into Cu_7_S_4_, there is sufficient space between the ultrafine nanoparticles for volume expansion. The UCu_7_S_4_NPs electrochemically restructured in a conductive graphene network are evenly distributed and effectively accommodate volume changes and mechanical stresses, avoiding agglomeration during repeated electrochemical cycling, thereby obtaining a cathode with a stable structure.

##### Optimization of Surface Activity

Uneven deposition of products on the surface of the conversion‐type cathode materials during battery charging and discharging is the primary cause of volume expansion. By optimizing the surface activity of the cathode carrier, it is possible to induce a reduction in the size of the discharge products after the conversion reaction. This optimization promotes uniform dispersion of discharge products across the cathode surface, thereby suppressing drastic volume changes in the cathode structure during charging and discharging cycles. Specifically, introducing some substances into the cathode carrier and increasing its surface activity through methods such as heat treatment and freezing can effectively adjust the morphology and distribution of discharge products. This approach minimizes volume changes, preserves the structural integrity of the cathode, and extends its lifespan.

For example, He et al.^[^ ^127^ ^]^ utilized phosphorus‐rich and nitrogen‐rich chelating resin with NiCl_2_⚫6H_2_O pyrolysis to synthesize Ni_2_P@C composites, embedding nickel particles to form Ni@NPC composites for Na–S battery cathodes. Scanning electron microscopy (SEM) images of the Ni@NPC/S (Figure 16j) showed that elemental sulfur was uniformly dispersed and confined within the carbon matrix. This uniform dispersion facilitated storage and electrolyte diffusion of sulfur, controlled the conversion reaction between sulfur and sodium sulfide to prevent the formation of long‐chain soluble polysulfides, stabilized short‐chain polysulfides, and inhibited repeated volume changes, thus maintaining structural integrity.

Similarly, Sun et al.^[^ ^178^ ^]^ formed MnO/C nanocomposites using a chemical vapor deposition (CVD) method, employing porous MnO as a template. After removing MnO, they obtained layered carbon spheres (PCSs) for Na‐O_2_ battery cathodes. The SEM image of the PCS electrode after discharge (Figure 16k) revealed that the surface was covered with membrane‐like discharge products instead of micro‐sized NaO_2_ cubes. The PCS electrode's structure provided more active sites for NaO_2_ growth, leading to the formation of a membrane‐like morphology that decomposed more easily than the typical micro‐sized cubes. This structural adjustment alleviated volume changes and ensured the stability of the cathode.

In another example, Ma's team^[^ ^215^ ^]^ synthesized FeF_2_–RGO composites, which in situ generated FeF_3_–Fe–RGO composites for Na‐MH batteries. TEM images of the Fe and Ag regions after charge–discharge cycling (Figure 16l) showed that the metallic Fe discharge products were uniformly distributed and stable throughout the cycling process. The minimal residual metallic Fe in the FeF_3_–RGO system contributed to a reduced degree of volume change on the cathode side, further mitigating volume expansion.

### 4.2 Poor Reaction Reversibility

Compared to other cathode materials, the primary advantage of conversion‐type cathodes lies in their high theoretical specific capacity and energy density. However, a significant gap persists between this theoretical potential and the achieved specific capacity in practical applications. The theoretical specific capacity defines the maximum energy storage capability of a battery, which is intrinsically linked to its stability and safety. When the actual specific capacity matches the theoretical value, achieving 100% capacity utilization, the battery's energy storage potential is fully realized. This represents an ideal state—maximizing resource efficiency and enhancing recyclability—which is a key future goal for conversion‐type cathodes. Nevertheless, in experimental sodium‐ion batteries, the actual specific capacity consistently falls short of the theoretical value and often exhibits rapid capacity degradation.

#### 4.2.1 Manifestations of Reaction Irreversibility

In addition, the energy density of the battery is proportional to the specific capacity and electric potential, which is a higher requirement relative to the value of the battery's specific capacity. A fast conversion reaction efficiency drives a higher energy density of the battery, which also increases the specific capacity. Unfortunately, the complexity of some of the conversion reactions, the presence of multiple substances at the same time, and the slowness of the reaction process will also reduce the actual specific capacity. As a result, the maximum advantage of the conversion‐type cathodes cannot be utilized, which seriously hinders the research process of high‐specific‐capacity batteries.^[^ ^284^ ^]^ The effects of the phenomenon of low actual specific capacity on different types of conversion‐type cathodes are as follows:

Na–S Battery: Among the SIBs composed of conversion‐type cathodes, Na–S batteries have the highest theoretical specific capacity, which is the key to realizing high specific capacity batteries. Existing studies show that the S cathode can reach a theoretical specific capacity of 1675 mA h g^−1^ with complete discharge, and the theoretical energy density of Na–S batteries can reach 1274 Wh Kg^−1^.^[^ ^285^ ^]^ Since the energy density is directly proportional to the specific capacity and the electric potential, the theoretical energy density/theoretical specific capacity of Na–S is less than 1, and the smaller the electric potential is, the worse the conversion efficiency of the battery is. Therefore, it clearly shows that the conversion reaction of Na–S batteries is slow. Of course, due to the formation and shuttling of polysulfides during the conversion process, it also affects the reaction rate of the battery.

**Figure 17:**
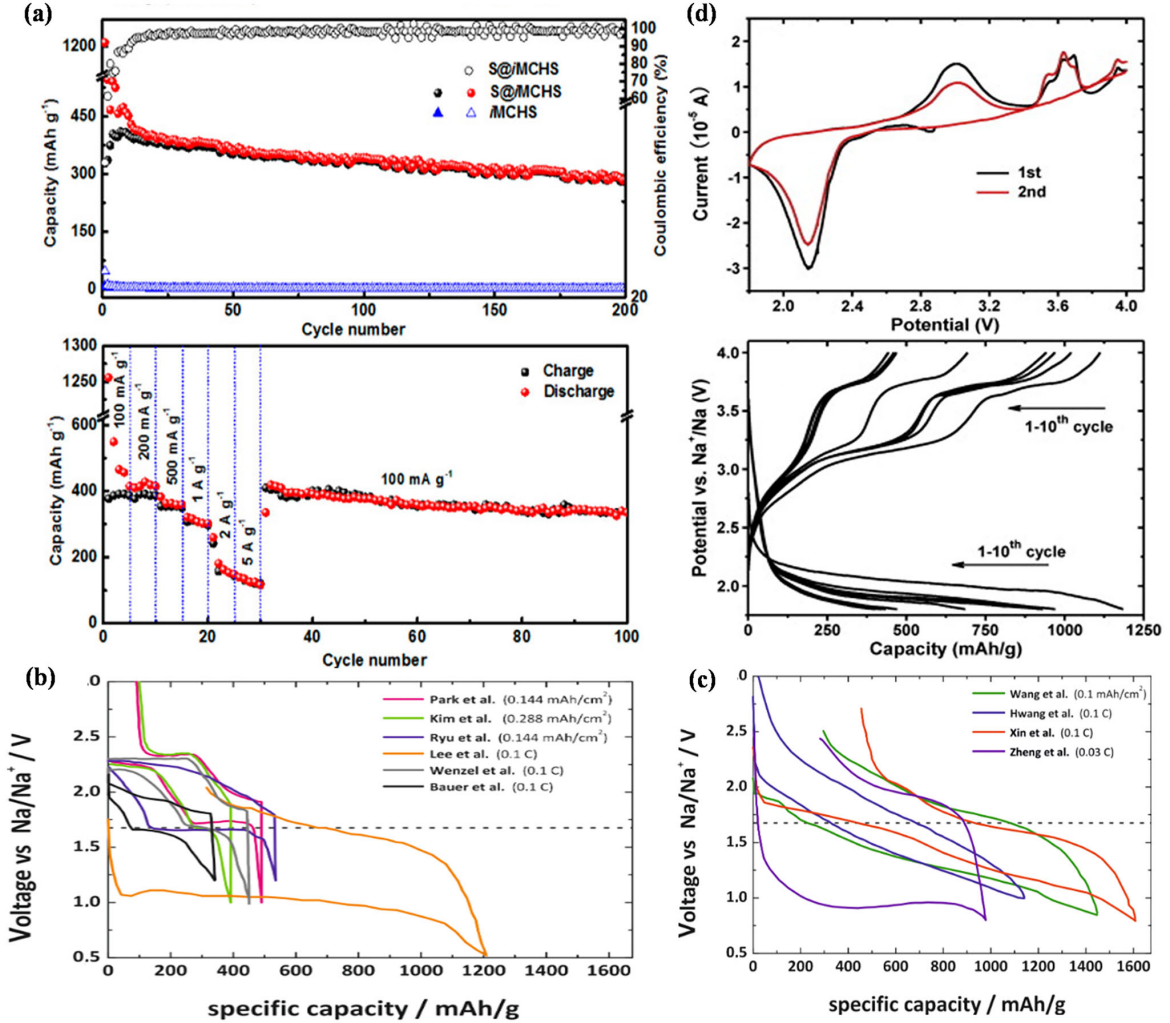
The image shows the irreversible specific capacity of the conversation‐type cathode. a) Specific capacity variation distribution of S@iMCHS cathode and rate capability at various current densities of the RT‐Na/S@iMCHS battery. Reproduced with permission.^[^ ^286^ ^]^ Copyright 2016, American Chemical Society. b) Na–S Batteries with ether‐based electrolytes. c) Na–S Batteries with carbonate electrolytes. Reproduced under the terms of the CC‐BY 2.0 license.^[^ ^287^ ^]^ Copyright 2015, Philipp Adelhelm et al. d) The initial two‐cycle profiles of cyclic voltammograms and Typical charge and discharge voltage distribution of NiCo_2_O_4_‐based cathode carrier of oxygen. Reproduced with permission.^[^ ^289^ ^]^ Copyright 2014, Elsevier.

Wang et al.^[^ ^286^ ^]^ proposed interconnected mesoporous carbon hollow nanospheres (iMCHS) as the sulfur cathode carriers, and with the help of **Figure** **17**a, tested the cycling performance of the S@iMCHS and iMCHS electrodes at 100 mA g^−1^. They found that, initially, the utilization of the S discharge capacity was as high as 1215 mAh g^−1^, but after 200 cycles, the capacity could only reach 292 mAh g^−1^, proving that the conversion reaction is irreversible. The generated Na_2_S not only did not undergo the oxidation reaction but also unevenly deposited on the cathode surface, resulting in a serious decline in battery capacity.

Similarly, Mou's group^[^ ^128^ ^]^ used N‐self‐doped porous carbon (NPC) sheets as the sulfur host material, and the initial discharge specific capacity was calculated to be up to 1,213.9 mAh g^−1^ based on the theoretical specific capacity of S. However, the reversible specific capacity was reduced to 418.9 mAh g^−1^ after 400 cycles at 0.5 °C. Thus, it is strongly proven that the actual specific capacity of Na–S batteries is low, and the capacity loss is large. In addition, the charge–discharge curves of existing Na–S batteries in different electrolytes (Figure 17b,c) show that the actual specific capacity is much different from the theoretical value.^[^ ^287^ ^]^

Na–O_2_ Battery: Compared with the S cathode, the theoretical specific capacity of the O_2_ cathode can be as high as 1670 mAh g^−1^, and the theoretical energy density of Na–O_2_ batteries can be as high as 3,164 Wh Kg^−1^.^[^ ^288^ ^]^ At a comparable specific capacity, the O_2_ cathode has faster reaction kinetics due to the fact that the conversion reaction steps and products of the Na–O_2_ battery are simpler compared to the S cathode. However, the additional reaction between the cathode and the electrolyte in Na–O_2_ batteries leads to a gap between the actual specific capacity and the theoretical value.

Hartmann et al.^[^ ^34^ ^]^ utilized a pure carbon cathode for Na–O_2_ batteries and achieved a discharge capacity of only 300 mAh g^−1^, which is much lower than the theoretical value. In addition, Liu et al.^[^ ^289^ ^]^ used NiCo_2_O_4_ nanosheets/Ni foam composites as a carbon‐free cathode carrier for Na–air batteries, and with the help of a typical charge/discharge voltage distribution diagram of NiCo_2_O_4_‐based electrodes (Figure 17d). It was observed that the first discharge capacity was 1185 mAh g^−1^, but only 33.8% of the initial discharge capacity (401 mAh g^−1^) remained at the 10th cycle. Thus, it is proven that the capacity of Na–O_2_ batteries decays too fast to ensure the cycling performance of the battery, even if the initial state reaches a high specific capacity.

Na‐MH Battery: Although the theoretical specific capacity of metal halide cathodes is relatively low compared to S and O_2_ cathodes, ranging from ≈500 to 1500 mAh g^−1^, the current specific capacity utilization is up to 95%, making them the most promising of the first battery with high specific capacity. In addition, among transition metal compounds, metal halide cathodes have the highest theoretical capacity and can be applied to various solid–liquid electrolytes. However, their specific capacity is not maximized due to their insulating properties and side reactions, etc.

Li et al.^[^ ^198^ ^]^ constructed a planar intermediate‐temperature (IT) Na‐NiCl_2_ battery, and with the help of the test on the specific capacity of the battery at 190 °C (Figure 11c), it was found that the initial capacity of the IT Na‐NiCl_2_ battery was 106 mAh g^−1^. Even after 200 cycles, the specific capacity stabilized at 137 mAh g^−1^, which is still far from the theoretical specific capacity, thus proving the low reversible specific capacity of Na‐MH batteries.

Other Batteries: In other conversion cathode batteries, there are more serious problems of low specific capacity and capacity attenuation. For example, a Na battery with a synthetic FeS_2_ cathode has a first discharge capacity of 447 mAh g^−1^ at room temperature, but after 50 cycles, the discharge capacity is only 70 mAh g^−1^. Kim and co‐workers^[^ ^67^ ^]^ also reported a sodium/pyrite FeS_2_ battery with a first discharge capacity of 630 mAh g^−1^, and the discharge capacity only remained at 85 mAh g^−1^ after 50 cycles. Similarly, for Na–Se/C batteries, a lower capacity was also observed, with a specific capacity of 265 mAh g^−1^ obtained after 50 cycles at 50 mA g^−1^.^[^ ^48^ ^]^

#### 4.2.2 Strategies Toward High Specific Capacity

Therefore, based on the current research on the conversion‐type cathodes (S, O_2_, and MH), it is essential to find ways to utilize cathode materials efficiently, summarize the experiences from existing high specific capacity batteries, and leverage the advantages of batteries with high energy densities to address the issues of low specific capacity utilization. In SIBs with conversion‐type cathodes, a common strategy to improve their reversible specific capacity is to introduce additives to lower the reaction barrier. Additionally, higher specific capacity can be obtained by adding (non)metallic elements to activate the product capacity and constructing a 3D network structure to enhance the Na^+^ transport rate. In the following sections, the strategies to improve the specific capacity will be described in detail from the above three aspects.

##### Lowering Reaction Barrier

To improve the electrochemical performance of batteries, additives are often incorporated to leverage their properties, thereby lowering the conversion reaction potential barrier and increasing the specific capacity to some extent. Based on the mechanism of the conversion reaction, which requires the transfer of multiple electrons, more energy is needed to facilitate rapid conversion. Typically, an additive is added to the cathode side of the battery to provide energy for the conversion reaction, promoting it to occur more easily and improving the reaction kinetics of sodium batteries.^[^ ^290^ ^]^ Moreover, the introduction of additives reduces the potential energy of the conversion reaction, shortens the time required for the reaction to proceed, accelerates product formation, and enhances conversion efficiency, ultimately increasing the battery's specific capacity.

For example, Zhang and Gong et al.^[^ ^122^ ^]^ prepared carbon nanofiber (CNF)‐based composites containing ferromagnetic CoFe_2_O_4_ as a carrier for S. By applying an external magnetic field, they increased the Na–S bond length in the product, while the spin‐polarized ferromagnetic additive lowered the reaction kinetic barrier. This resulted in the unprecedented specific capacity of 1097 mAh g^−1^ for SIBs based on the CNF/CoFe_2_O_4_/S cathode. Cyclic voltammetry (CV) measurements of the CNF/CoFe_2_O_4_ electrodes, both with and without the external magnetic field at a scan rate of 0.4 mV s^−1^, showed a minimal potential difference between the reduction and oxidation peaks, indicating improved conversion kinetics of the sodium polysulfides (SPSs).

**Figure 18:**
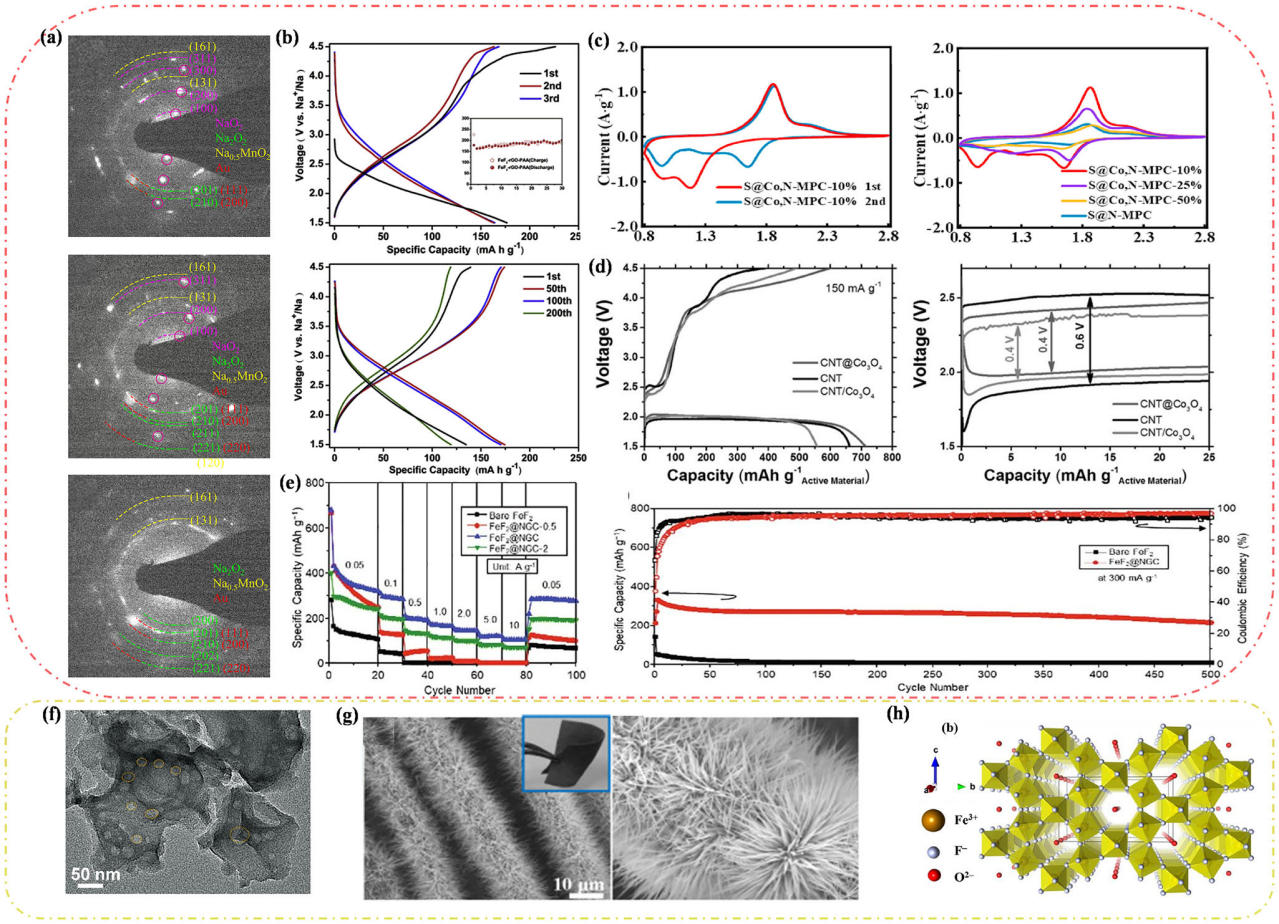
Summary diagram of irreversible specific capacity in conversion‐type cathodes. a) The variation of diffraction spots in the electron diffraction pattern (EDP) of the Au/MnO_2_ air cathode shows the changes in NaO_2_ during discharge. Reproduced with permission.^[^ ^291^ ^]^ Copyright 2019, Elsevier. b) Charge and discharge curves of the first three cycles of the FeF_2_‐rGO‐PAA electrode. The inset section displays the specific capacity distribution and voltage profiles for various cycles of the FeF_2_‐rGO‐PAA electrode. Reproduced with permission.^[^ ^213^ ^]^ Copyright 2019, Elsevier. c) First two cyclic voltammetry (CV) curves of S@Co, N‐MPC‐10%. The second CV curves of S@N‐MPC and S@Co, N‐MPC composites are also shown. Reproduced with permission.^[^ ^129^ ^]^ Copyright 2023, Elsevier. d) Comparison of charge and discharge profiles of sodium–air batteries (SABs) with atomic layer deposition (ALD) CNT@Co_3_O_4_, CNT/Co_3_O_4_ nanocomposite, and pristine CNT air electrodes at 150 mA g^−1^. Reproduced with permission.^[^ ^180^ ^]^ Copyright 2017, John Wiley and Sons. e) Rate capability at various rates from 0.05 to 10 A g ^−1^ of bare FeF_2_, FeF_2_@NGC‐0.5, FeF_2_@NGC, and FeF_2_@NGC‐2, along with high‐rate cycling performance of bare FeF_2_ and FeF_2_@NGC. Reproduced with permission.^[^ ^220^ ^]^ Copyright 2022, Elsevier. f) Transmission electron microscopy (TEM) images of GeO_x_/NC composites. The yellow dotted circle highlighted the porous structure. Reproduced with permission.^[^ ^116^ ^]^ Copyright 2023, Elsevier. g) Scanning electron microscopy (SEM) images of the carbon textile/Co_3_O_4_NWs (COCT) cathode at low and high magnification. Reproduced with permission.^[^ ^181^ ^]^ Copyright 2016, Elsevier. h) Crystallographic structure of HTB‐FeF_3_·0.33H_2_O. Reproduced with permission.^[^ ^231^ ^]^ Copyright 2018, Elsevier.

Liu's group^[^ ^291^ ^]^ used ion sputtering to encapsulate gold nanoparticles in manganese dioxide (MnO_2_) nanowires as oxygen carriers. In comparison to the bare MnO_2_ air cathode, the discharge products nucleate or grow along the Au nanoparticles during the discharge process. Their findings indicated that the main discharge reaction occurred only in the presence of the Au nanoparticles, demonstrating a significant catalytic effect that reduced the free energy of the reaction and facilitated the rapid generation of discharge products. Specifically, the rapid disproportionation of NaO_2_ formed on Au/MnO_2_, as illustrated in **Figure** **18**a, suggests that either Au or MnO_2_ can promote the chemical decomposition of NaO_2_, thereby increasing the specific capacity.

Ni's research team^[^ ^213^ ^]^ prepared FeF_2_–rGO nanocomposites containing binder polyacrylic acid (PAA) as the cathode of Na‐MH batteries. They discovered for the first time that the FeF2–rGO–PAA electrode exhibited an increase in specific capacity after the charge–discharge process, rising from an initial capacity of 180 to 200 mAh g^−1^ after 30 cycles (Figure 18b). This increase can be attributed to the strong adhesive properties of PAA, which reduced the barrier to the conversion reaction and resulted in a higher capacity.

##### Metal Activated Cathode Materials

Studies indicate that incorporating specific elements into the cathode can enhance cathode activity and boost the battery's specific capacity. Specifically, adding metallic elements (e.g., Co) or novel metal‐containing compounds facilitates strong synergistic interactions—such as inhibition, decomposition, and catalysis—with otherwise inactive discharge products not participating in the conversion process. This activates these cathodic products, incorporating them into the conversion reaction pathway. Consequently, active material utilization increases, enabling sustained reversible conversion within the battery. Non‐metallic elements can similarly enhance batteries with diverse properties. These approaches not only achieve a high specific capacity after many cycles but also help prevent rapid capacity decay.

For example, Liu et al.^[^ ^129^ ^]^ utilized a microporous carbon matrix (Co, N‐MPC) prepared by incorporating cobalt and nitrogen elements, using CoZn‐ZIFs as the precursor, as the S cathode carrier for Na–S batteries. By adjusting the ratio of Co in the zeolite imidazoline framework (ZIF), they effectively utilized the advantages of nitrogen and abundant micropores to strongly interact with the pyrolysis products and sulfur, thereby activating the capacity of active sulfur. This resulted in a high specific capacity of 1134.63 mAh g^−1^ after 100 cycles at 0.2 °C. Furthermore, the CV curve of N‐MPC‐10% at a scanning speed of 0.1 mVs^−1^ in the voltage range of 0.8–2.8 V confirmed the S@Co (Figure 18c). The peaks observed during the positive and negative scans are closely related to the conversion process of sulfur, with the two oxidation curves nearly overlapping, indicating that the conversion reaction is reversible. Notably, the peaks for S@Co, N‐MPC‐10% were the most pronounced, indicating the most complete conversion of reactive sulfur species.

Sun and his colleagues^[^ ^180^ ^]^ employed atomic layer deposition (ALD) to modify Co_3_O_4_ within carbon nanotubes, resulting in CNT/Co_3_O_4_ nanocomposites for use in Na–O_2_ batteries. Co_3_O_4_ facilitates the decomposition of both peroxides and superoxides of the discharge products, reducing the surface coverage of products on the air electrode and removing obstructions to O_2_ passage. This enhancement increases O_2_ utilization and facilitates greater capacity development of the active material, resulting in a high specific capacity of 715 mAh g^−1^ (Figure 18d).

Maulana and colleagues^[^ ^220^ ^]^ used Fe(NO_3_)_3_⚫9H_2_O as an iron source, along with dopamine containing catechol and amine groups, to prepare encapsulated FeF_2_ nanoparticles within N‐doped graphitic carbon (FeF_2_@NGC), successfully preventing the undesirable phase transition of FeF_2_ to Na_3_FeF_6_ and FeF_3_. This improvement enhanced the invertibility of the conversion reaction, resulting in FeF_2_@NGC exhibiting a reversible specific capacity of 214.2 mAh g^−1^ (see Figure 18e).

Wang et al.^[^ ^30^ ^]^ successfully designed HCS@Se by introducing heteroatoms into hollow carbon spheres using the steam infiltration method. The introduction of oxygen‐rich groups and N elements induces the evolution of electron transfer behavior, resulting in improved interfacial electron transfer. Thus, the Se electrode is activated during cycling, improving the utilization of the active material, due to the interfacial C–Se bond and the distribution of Se. As a cathode in the Na–Se system, it also has a specific capacity of 593.9 mAh g^−1^ after 100 cycles at a current density of 0.1 C.

##### Enhancing Reaction Kinetics

In addition to the above strategies mentioned above, the reversible specific capacity can also be enhanced by establishing a 3D network structure on the cathode side to improve the ion transport rate. Based on the mechanism of the conversion reaction, the process requires a constant supply of Na⁺. Therefore, timely supplementation of Na⁺ during the conversion reaction can help accelerate the overall reaction process. Specifically, researchers employ hot‐melt, hydrothermal, and other chemical methods to composite materials that produce a cathode with a 3D network structure. These channels serve as the fundamental transmission pathways for ions and electrons, continuously delivering Na⁺ during the charging and discharging processes to increase the concentration of Na⁺ measured by the electrode and promote the conversion reaction.

Furthermore, building upon this 3D network structure, a new configuration can be constructed to provide shorter transport pathways for Na⁺ ions, thereby accelerating the conversion reaction rate and achieving a high reversible capacity. Ma and his coworkers^[^ ^94^ ^]^ synthesized an amorphous GeO_x_/NC composite material as an S cathode carrier using a melt diffusion method with ethylenediamine (EDA) serving as both a carbon and nitrogen source along with GeO_2_. The interconnected 2D nanosheets were assembled into a 3D structure, establishing a continuous ion/electron network that accelerates ion and electron migration. Additionally, in the ether electrolyte, the metal sulfide phase formed a stable porous structure during charging and discharging, significantly reducing the migration distance of Na⁺, thus providing impressive rate capability (Figure 18f). This material exhibits a reversible capacity of 860 mAh g^−1^ over 1000 cycles with no significant capacity decay.

Li et al.^[^ ^181^ ^]^ used hydrothermal synthesis of CO_3_O_4_ nanowires on carbon textiles as cathode carriers for Na–O_2_ batteries. The CO_3_O_4_ NWs were grown vertically and uniformly on the carbon framework without the use of a binder, as seen in Figure 18g. The generation of pin‐pricked structures on the surface of the carbon fibers contributes to the formation of a low‐resistance transport path for the rapid migration of sodium ions. This shorter transport pathway, compared to that provided by the carbon framework, allows the cathode material to exhibit excellent transport properties for both electrons and ions, thereby increasing the energy density of the battery.

Wei's team^[^ ^231^ ^]^ utilized a solvothermal method to synthesize hydrogen fluoride (HF) and ferric nitrate (III) non‐hydrated iron (Fe(NO_3_)_3_⚫9H_2_O) as the fluorine and iron sources, respectively, resulting in spherical FeF_3_⚫0.33H_2_O/MWCNTs cathodes with a mesoporous structure. The intertwined MWCNTs were employed to form a mesh structure, constructing a 3D electron‐conducting network that facilitates the diffusion of sodium ions. Moreover, the hexagonal tungsten–bronze (HTB) FeF_3_·0.33H_2_O structure provides ample accommodation and transport space for Na⁺ due to its large hexagonal cavity (Figure 18h), which enhances the rate capability of the electrode material, resulting in a higher specific capacity.

Zeng et al.^[^ ^267^ ^]^ added Se to 3D interconnected mesoporous carbon nanofibers to prepare an additive‐free flexible film electrode, Se@PCNFs, for high‐performance Na–Se batteries. First, the 3D interconnected PCNFs framework has excellent electronic conductivity, which will greatly reduce the time required for electron and ion transport. Second, the high porosity of PCNFs helps ions diffuse in the electrolyte, thereby accelerating the transport rate of sodium ions, resulting in faster reaction kinetics and exhibiting high reversible capacity (520 mAh g^−1^ after 80 cycles at 0.05 A g^−1^).

### 4.3 Severe Surface Side Reactions

Since the operation of a conversion‐type cathode battery involves a multistep chemical reaction, potential reactions can occur between various substances within the battery. This, in turn, results in the accumulation of excess products, leading to a significant decrease in the electrochemical performance of the battery. In theory, the battery undergoes electron and ion transfer, with oxidation reactions at the anode and reduction reactions at the cathode. However, in practice, side reactions often accompany these primary reactions.

#### 4.3.1 Manifestations of Side Reactions

**Figure 19:**
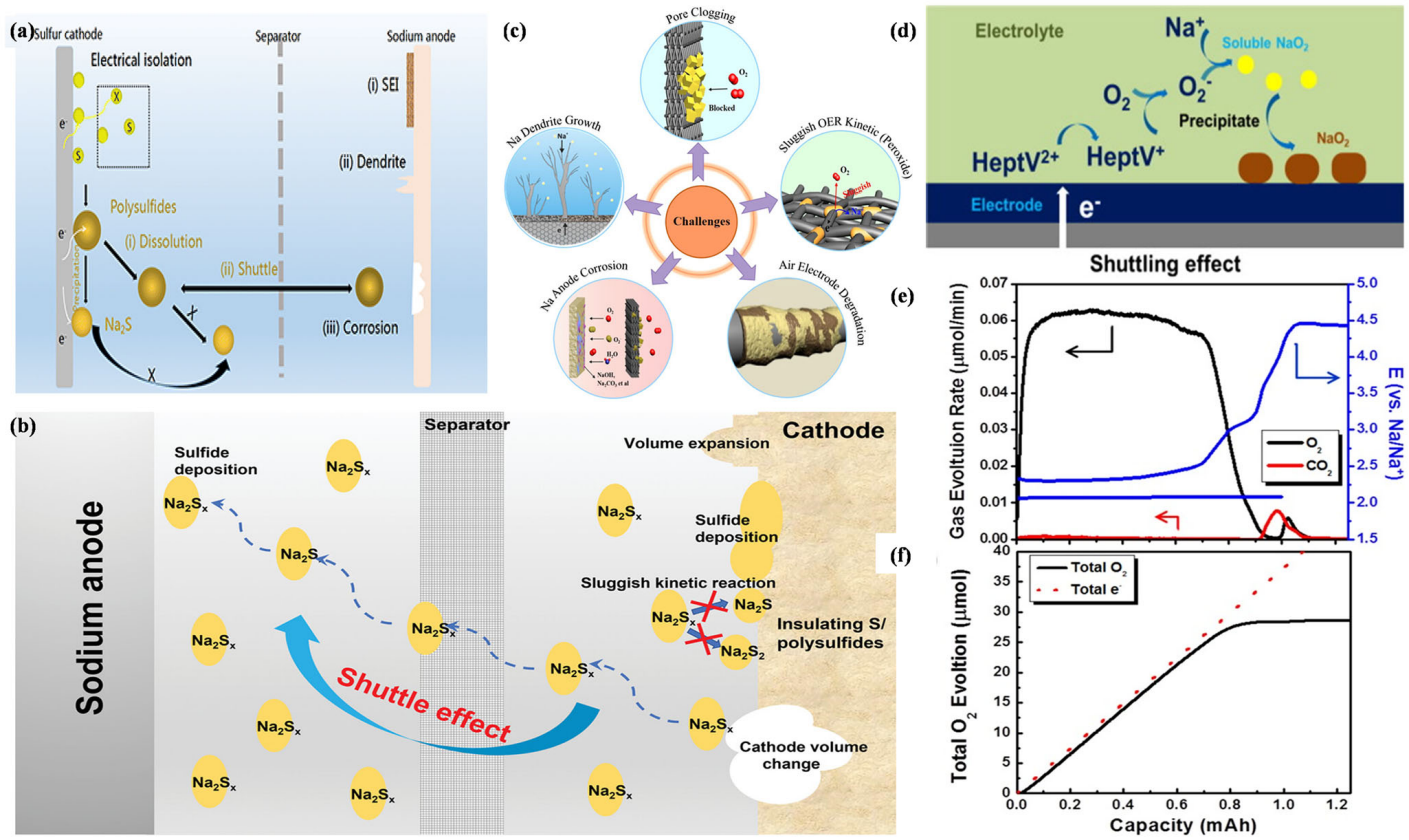
The image illustrates the side reaction of sodium metal batteries comprising conversion‐type cathodes. a) Problems faced by sodium–sulfur batteries. Reproduced with permission.^[^ ^292^ ^]^ Copyright 2003, Royal Society of Chemistry. b) Shuttle effect in RT Na–S battery. Reproduced under the terms of the CC‐BY 4.0 license.^[^ ^49^ ^]^ Copyright 2022, Xiaoting Lin et al. c) Key challenges for sodium–oxygen battery electrodes. Reproduced with permission.^[^ ^294^ ^]^ Copyright 2019, John Wiley and Sons. d) Model diagram of the shuttle effect of HeptVBr_2_ on NaO_2_. Reproduced with permission.^[^ ^299^ ^]^ Copyright 2023, Elsevier. e) The Na–O_2_ battery discharge/charge curve during the first cycle, indicating the beginning of electrolyte oxidation at a flat curve of 4.4 V, and f) Comparison of integral values of oxygen evolution (solid black line) with theoretical integral values of oxygen evolution (dotted red line). Reproduced with permission.^[^ ^296^ ^]^ Copyright 2016, John Wiley and Sons.

On the cathode side, excluding avoidable cases such as artificial operation and introduction of impurities, the side reactions are mainly manifested as improper operation and the introduction of impurities. The side reactions primarily manifest as additional interactions among the product, electrolyte, and anode. These include repeated diffusion of the product in the electrolyte, reactions between the electrolyte and the consumed product, and interactions between the electrode and the electrolyte, leading to erosion. As a result, most batteries experience side reactions. When these additional reactions occur, the conversion reaction rate decreases, the electrode may deform, and the electrolyte can decompose, resulting in low reversible capacity, poor cycling stability, short cycle life, and, in severe cases, battery failure or even explosion. The phenomena and effects of side reactions in different types of conversion‐type cathodes are as follows:

Na–S battery: When S is used as the conversion‐type cathode in SIBs, the side reaction is primarily manifested as a “shuttle effect” (see **Figure** **19**a),^[^ ^292^ ^]^ which leads to the loss of active substance and a decline in capacity. During the charging and discharging process, soluble long‐chain sodium polysulfides (NaPSs) produced by the conversion reaction of sulfur are generated first.^[^ ^293^ ^]^ Driven by the electric field and concentration gradients, these long‐chain sodium polysulfides migrate from the cathode side to the anode side through the electrolyte, where side reactions occur, resulting in the irreversibility of the conversion reaction (see Figure 19b).^[^ ^294^ ^]^

Additionally, some short‐chain polysulfides generated by reactions at the anode can diffuse back to the cathode side and participate in subsequent conversion reactions, generating more long‐chain sodium polysulfides, which contribute to the self‐discharge phenomenon of the battery. Yang et al.^[^ ^295^ ^]^ observed the diffusion of sodium polysulfides, NaPSs, and the discharge capacity of the battery with the help of the preparation of different separators for use in Na–S batteries. It was found that the discharge capacity of the battery decreases as the diffusion of NaPSs in the anode increases, thus demonstrating that the Na–S battery undergoes a “shuttle effect”.

Na–O_2_ battery: Compared with S cathode, when using an O_2_ conversion‐type cathode, the side reactions are primarily manifested as electrolyte dissolution, electrode passivation (Figure 19c), and the shuttle effect (Figure 19d), all of which directly impact the normal operation of the battery.^[^ ^143^ ^]^ During the discharge process, reduced oxygen species (O_2_−, HOO−, HO−) and single‐linear oxygen (^1^O_2_) are formed in the Na–O_2_ battery, which then react with the electrolyte. Notably, the nucleophilic attack of O_2_− on organic electrolytes generates by‐products (CO_2_, H_2_O, carboxylic acids, etc.), affecting the reversibility of the conversion reaction and severely hindering the cycling process. This issue is further influenced by the presence of H_2_O or other proton sources in the electrolyte.^[^ ^147^ ^]^

For example, Black et al.^[^ ^296^ ^]^ utilized diphosphor as an electrolyte to prepare Na–O_2_ batteries for by‐product exploration and found that NaO_2_ and solvated O_2_^−^ side‐reacted with both the carbon cathode and the diethylene glycol dimethyl ether electrolyte, producing Na‐carboxylate decomposition products that could not be decomposed in Na–O_2_ batteries. These by‐products progressively accumulated during battery charge/discharge cycling, leading to battery failure. Additionally, the oxidation process of the Na–O_2_ battery electrolyte is illustrated in Figure 19e,f.

Na‐MH battery: In SIBs with transition metal halides as cathodes, side reactions primarily manifest as the erosion of the cathode structure and the decomposition of the electrolyte. The presence of metal elements, particularly active metals, makes them prone to dissolution in the electrolyte, resulting in degradation of the electrode structure and the loss of active substances, which leads to capacity decline.^[^ ^297^ ^]^

Additionally, some metals are in direct contact with the electrolyte, consuming it and reducing the battery's service life. Zhan's team^[^ ^200^ ^]^ investigated the issues caused by the dissolution of Ni and Fe in batteries by testing the solubility of FeCl_2_ and NiCl_2_ using ICP‐OES. They found that FeCl_2_ has the highest solubility (0.06 mol%) in NaAlCl_4_, leading to the extraction of Na⁺ from the base to form γ‐alumina and consequently consuming the electrolyte. Furthermore, the Fe particles appeared to be crushed, which disrupted the electron conduction network, rendering them electrochemically inactive and contributing to the capacity decline of the battery.

Other batteries: Na–Se batteries, like Na–S batteries, also suffer from the “shuttle effect” problem. In Na–Se batteries, when long‐chain polyselenides are formed, they will be driven by the electric field and concentration gradient to move to the anode to reduce short‐chain and insoluble polyselenides. Among them, insoluble polyselenides can cause electrode passivation and enhanced polarization, and short chains can be oxidized again on the cathode side. In the following cycle, they will repeatedly diffuse between the cathode and anode, producing the “shuttle effect”,^[^ ^104^, ^298^ ^]^ while in sodium–metal sulfide and oxide batteries, the surface of the electrode often becomes rough during cycling, and the by‐reaction of gradual pulverization often occurs.^[^ ^251^ ^]^

#### 4.3.2 Strategies Toward Stable Cycling Performance

To address the aforementioned side reaction issues, researchers have focused on modifying the surface of the cathode carrier to create a protective barrier or a hindering mechanism. Some scholars^[^ ^300^ ^]^ have attempted to shield the cathode by constructing a coating on its surface, which can prevent reactions with highly oxidizing substances in the battery. However, thinner coatings have been found to provide only short‐term protection and can negatively impact Na⁺ ion mobility to some extent. Conversely, leveraging metal polarity, anchoring discharge products, and introducing active substances to increase reaction sites can impede the dissolution and reaction of discharge products in the electrolyte, leading to an extended cycle life. Additionally, adjusting the size of micropores and inhibiting the detrimental transformation of products can prevent the diffusion of discharge products into the electrolyte, thereby addressing the side reaction problem. The application of these three strategies in conversion‐type cathodes for S, O_2_, and Na‐MH batteries will be discussed in detail in the following sections.

##### Metal Anchored Products

Anchoring discharge products on the surface of conversion‐type cathodes and preventing their dissolution in the electrolyte can effectively inhibit the occurrence of side reactions. Carbon‐based cathodes generally achieve high reactant utilization efficiency. However, they lack the ability to form strong interactions with discharge products to inhibit their dissolution into the electrolyte.^[^ ^301^ ^]^ Therefore, an “intermediary” is introduced into the battery, which anchors the discharge products to the cathode carrier, thereby reducing the likelihood of side reactions. The anchoring materials for discharge products typically include transition metal compounds, such as metal oxides and metal sulfides, which chemically capture soluble products in the electrolyte through polar metal bonds.

**Figure 20:**
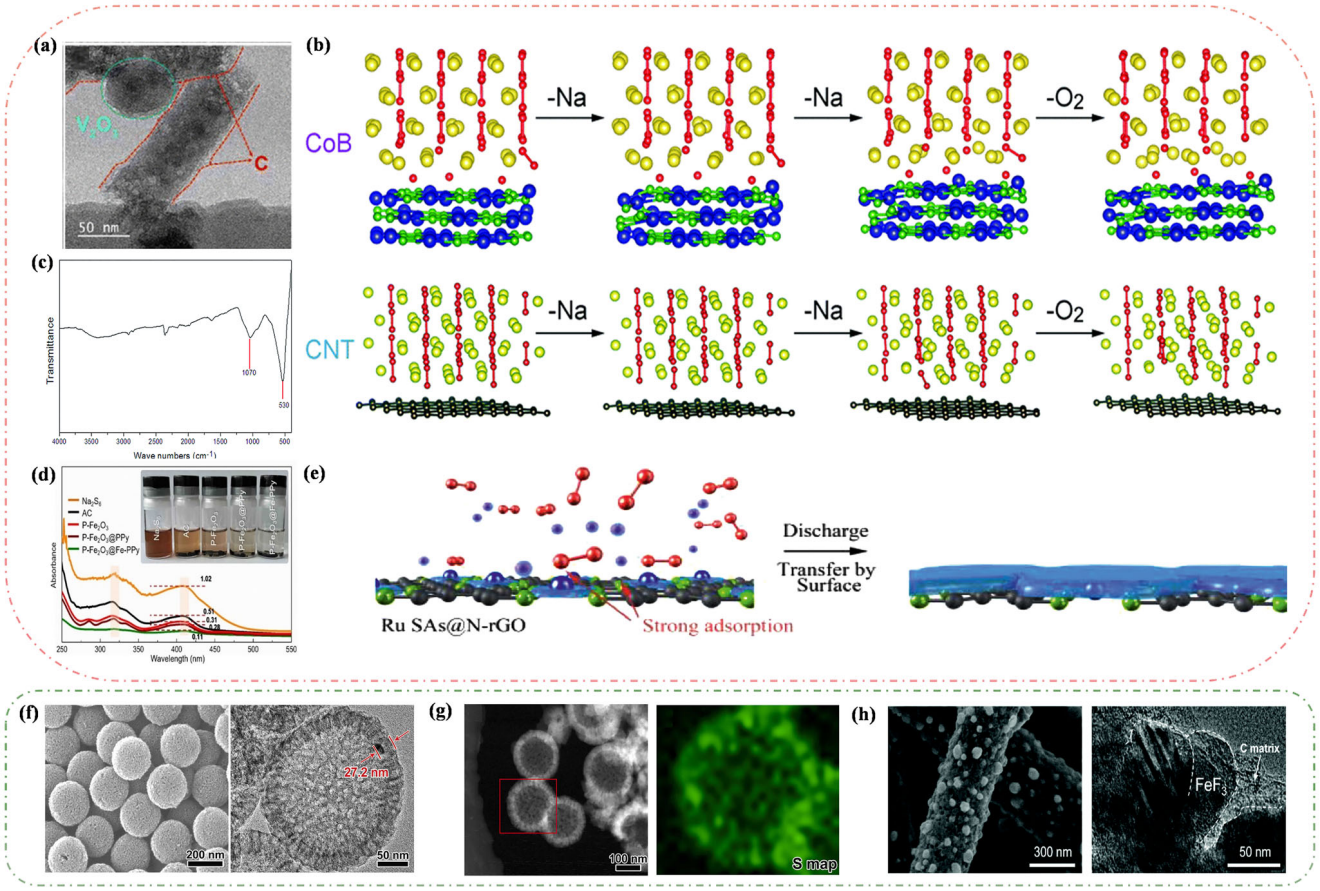
Summary diagram of side reactions on the conversation‐type cathode side. a) HRTEM images of the SeS_2_/V_2_O_3_@C. Reproduced under the terms of the CC BY‐NC 3.0 license.^[^ ^302^ ^]^ Copyright 2018, Xianglong Huang et al. b) IR spectra of nanosheets FeF_3_/graphene. Reproduced with permission.^[^ ^153^ ^]^ Copyright 2011, RSC Publishing. c) Geometric change of adsorbed Na_2_O_2_ during charging. Reproduced with permission.^[^ ^212^ ^]^ Copyright 2008, RSC Publishing. d) UV/V is the spectra of pure Na_2_S_6_ solution mixed with other cathode materials (insert photos of each solution). Reproduced with permission.^[^ ^303^ ^]^ Copyright 2023, John Wiley and Sons. e) Schematic diagram of RuSAs inducing surface‐mediated discharge through strong adsorption of intermediate NaO_2_. Reproduced with permission.^[^ ^182^ ^]^ Copyright 2022, Elsevier. f) SEM images and TEM image of MHCS, and g) STEM image of S@MHCS‐3 after cycling, along with the corresponding elemental mappings of sulfur. Reproduced with permission.^[^ ^305^ ^]^ Copyright 2023, Elsevier. h) SEM image magnification and high‐resolution TEM image of FeF_3_/CNFs. Reproduced with permission.^[^ ^232^ ^]^ Copyright 2012, Royal Society of Chemistry.

For example, Huang et al.^[^ ^302^ ^]^ modified porous carbon with polar V_2_O_3_ and then compounded it with activated SeS_2_ to form a SeS_2_/V_2_O_3_@C cathode for Na–S batteries. The tight coupling between carbon nanorods and V_2_O_3_ is clearly evident in the TEM image of SeS_2_/V_2_O_3_@C (**Figure** **20**a), indicating that V_2_O_3_ firmly anchors the surface and ensures structural stability without compromising the carbon structure. The addition of strongly polar V_2_O_3_ inhibits side reactions by interacting with polysulfides through Na─O and V─S bonds, anchoring the polysulfides on the cathode surface and preventing their dissolution in the electrolyte.

Ma and his colleagues^[^ ^153^ ^]^ synthesized porous CoB nanosheets modified on the surface of carbon nanotubes as a cathode for Na–O_2_ batteries, using cobalt chloride and sodium borohydride as raw materials after heat treatment. During the discharge process, the strong interaction between Na_2_O_2_ and the CoB interface is utilized, breaking the O─O bond of Na_2_O_2_ near the CoB interface and immobilizing it on the CoB surface, effectively avoiding side reactions (see Figure 20b). Notably, after charging eliminates the Na_2_O_2_ on the CoB surface, the remaining Na_2_O_2_ is spontaneously attracted back to the surface, allowing the reaction to continue until the adsorbed Na_2_O_2_ is completely decomposed.

Shen's team^[^ ^212^ ^]^ prepared FeF_3_/graphene composites with a lamellar structure as the cathode for Na‐MH batteries using the sol–gel method. Since the conversion‐type cathode FeF_3_ is a transition metal compound, the C─F bond between FeF_3_ and graphene eliminates the need for additional substances, allowing FeF_3_ to be better anchored on the graphene surface. Furthermore, the FTIR spectroscopy scan of the sample (Figure 20c) confirmed the existence of C─F bonds ≈1070 cm^−1^. Therefore, the anchoring of FeF_3_ effectively prevents the dissolution of active metals in the electrolyte and inhibits the generation of by‐products.

However, in the study of Na–Se batteries, it was found that carbon can form a strong interaction with the discharge products. Zeng et al.^[^ ^261^ ^]^ prepared selenium/carbon composite materials (Se@CNFs‐CNT) by co‐heating selenium powder and electrospun polyacrylonitrile (PAN)‐carbon nanofibers in a sealed environment. Carbon nanotubes, as an additive, form CNFs‐CNT as a host to restrict Se molecules through C–Se bonds, inhibiting the dissolution and shuttle phenomenon of polyselenide. In addition, by comparing the cycle performance of Se@CNFs‐CNT and Se@CNFs electrodes, the current efficiency of Se@CNFs‐CNT is almost 100%, indicating that the shuttle reaction has indeed been effectively inhibited.

##### Increasing Active Sites

Increasing the number of reaction sites on the surface of the cathode carrier can lead to a tighter binding of discharge products and utilize the catalytic effect to improve reaction kinetics, thereby reducing the generation of by‐products. Based on the mechanism of conversion reactions, the multistep electrochemical reaction of conversion cathodes can be limited by the slow conversion of intermediate products. Additionally, the charge transfer process is hindered due to the wide band gap of the products.^[^ ^177^ ^]^ Under slow reaction kinetics, the continuous accumulation of products on the cathode side leads to electrode passivation, adversely affecting subsequent charge/discharge reactions. Therefore, adding active substances containing metal elements to increase the active sites for the products, along with leveraging the catalytic properties of these substances, can promote the conversion reaction,^[^ ^130^ ^]^ facilitating the rapid formation of insoluble products from intermediates and preventing side reactions.

Zhang's group^[^ ^303^ ^]^ prepared porous Fe_2_O_3_ core–shell structured composites with monolithic sulfur encapsulated by a Fe(CN)_6_^4−^‐doped polypyrrole polar film as the cathode. Interfacial polymerization was initiated by adding an appropriate amount of Fe(CN)_6_^4−^ as an oxidant, creating abundant Fe redox catalytic sites. The catalytic effect of the Fe and metal oxide centers was utilized to significantly accelerate the sulfur redox process. Fe acted as a redox mediator, promoting the complete conversion of medium‐chain Na_2_S_4_ to Na_2_S, thus suppressing the shuttle effect (Figure 20d).

Jin and his colleagues^[^ ^182^ ^]^ prepared ruthenium single atoms (Ru‐SAs) dispersed on nitrogen (N)‐doped reduced graphene oxide (Ru‐SAs@N‐rGO) as cathode materials. With Ru atoms anchored by four nitrogen atoms on the cathode surface and highly dispersed, more Ru–N sites were available for the products, which facilitated the in situ conversion of NaO_2_ to Na_2_₋_x_O_2_ by leveraging the stronger chemisorption effect on the intermediate superoxide, thereby improving reaction kinetics (Figure 20e) and inhibiting the dissolution and transport of NaO_2_ in the electrolyte.

Zhu et al.^[^ ^304^ ^]^ prepared Pt/Ir‐doped carbon nanotubes (Pt_0.8_Ir_0.2_@CNT) as an air cathode. The uniform distribution of Pt/Ir nanoparticles within carbon nanotubes formed active reaction sites. During the discharge process, Na^+^ rapidly diffuses to the cathode, generating hollow spheres at the Pt_0.8_Ir_0.2_ sites on the nanotube surface. The charging process also demonstrated that the hollow spheres contracted rapidly and disappeared, indicating that the addition of the bimetallic Pt_0.8_Ir_0.2_ catalysts accelerated the process of oxygen reduction reaction (ORR) for the discharged product growth and complete decomposition during the oxygen evolution reaction (OER), thus avoiding the formation of by‐products.

##### Regulating Surface Micropores

In addition to the two methods mentioned above, side reactions can be inhibited through the physical approach of surface microporous wrapping, which prevents the malignant transformation of intermediate products. Specifically, the pores on the surface of the cathode carrier and the discharge products should be of an appropriate size. This ensures that while they inhibit additional reactions, they do not hinder the interaction with Na⁺ ions in the electrolyte.^[^ ^272^ ^]^ Appropriately sized defects within the cathode enable effective confinement of reactants or discharge products. Optimal suppression of undesirable side reactions—including conversion to hazardous compounds and electrolyte dissolution—requires intimate contact between these trapped species and the cathode framework.^[^ ^131^ ^]^ Selecting the appropriate pore size for the product can be challenging. Therefore, modulating the pore size, physically constraining the products, generating and decomposing discharged products within the pores, and limiting the formation of certain intermediate phases can effectively minimize by‐product generation.

Luo's research team^[^ ^305^ ^]^ confined sulfur copolymers within the inner nanopores of sulfhydryl‐functionalized mesoporous hollow carbon spheres (MHCS) to create the S@MHCS cathode for Na–S batteries. The TEM and SEM images (Figure 20f) clearly show the prominent nanopore structure of the MHCS. The use of surface nanopores to physically confine sulfur monomers inhibited the formation of soluble long‐chain polysulfides during the conversion reaction. Furthermore, a homogeneous distribution of sulfur and a strong signal were observed in cyclic SEM images before and after the introduction of the S@MHCS‐3 cathode (Figure 20g), confirming the suppression of the shuttling effect.

Ma et al.^[^ ^306^ ^]^ employed an atomic deposition method to prepare ZnO/C and anchor palladium (Pd) nanoparticles on the surface, resulting in Pd/ZnO/C as an O_2_ carrier. During the discharge process, a feather‐like structure formed on the cathode surface. As the number of cycles increased, the “feathers” thinned and fragmented into nanowires, creating numerous pores. These pores not only facilitate the entry of oxygen into the cathode but also store more discharge products, thereby preventing their diffusion into the electrolyte.

Sun and his colleagues^[^ ^232^ ^]^ prepared FeF_3_‐C nanocomposite cathodes using sodium difluoroborate (NaDFOB) to form Na‐MH batteries with Fe(acac)_3_ and polyacrylonitrile (PAN) nanofibers (NFs). The SEM image of FeF_3_/CNFs (Figure 20h) reveals that the high specific surface area and void structure of the carbon matrix network securely embed FeF_3_, protecting it from electrolyte dissolution and thus avoiding by‐product formation.

Luo et al.^[^ ^239^ ^]^ prepared a Se/C composite cathode by injecting cyclic Se_8_ into mesoporous carbon at high temperatures. The mesoporous carbon formed spherical particles, uniformly filling the mesoporous carbon spheres with Se, and increasing the average pore size from 1.6 to 4.1 nm. In the Na–Se battery, the low‐order polyselenide intermediates formed during the sodiumation process are confined to the mesoporous carbon and stabilized in the pores of the mesoporous carbon, avoiding the shuttle reaction of polyselenide. Therefore, the Na–Se battery using the Se/C composite cathode has stable cycle performance. It can provide a high reversible capacity after 1000 charge–discharge cycles without any capacity loss during this period.

## 5 Conclusions and perspectives

**Figure 21:**
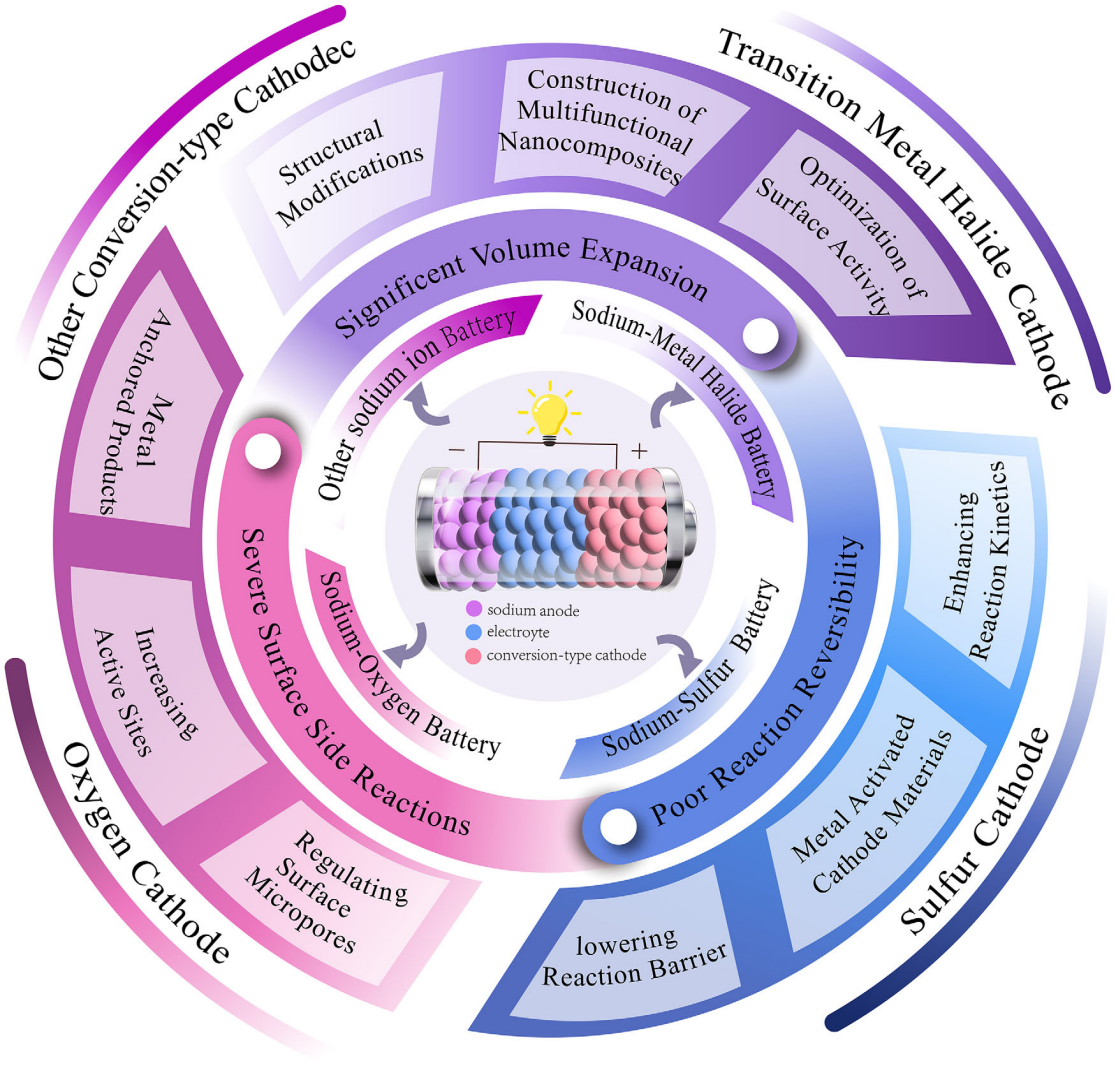
Overview of the problems faced by conversion‐type cathodes and corresponding strategies to address these challenges.

In summary, this work comprehensively reviews conversion‐type cathode materials for sodium‐ion batteries, with a particular focus on their fundamental reaction mechanisms. The multi‐electron conversion process provides these cathodes with several compelling advantages, including high theoretical energy density, intrinsic safety, environmental compatibility, and the flexibility to utilize a wide range of abundant elements. Among these benefits, the high energy density highlights the strong potential of conversion‐type cathodes for next‐generation SIBs, while the abundance and low cost of raw materials further enhance their practical appeal. In addition, their low‐toxicity or nontoxic characteristics improve recyclability, reduce environmental impact, and offer distinct safety advantages, especially in oxygen‐containing or metal–halide systems. Building on the conversion reaction mechanism, we discuss the major challenges and corresponding strategies associated with conversion‐type cathodes. The conversion process typically induces substantial volume changes and partially irreversible transformations, which can trigger side reactions that degrade cycling stability and shorten battery lifespan. Among the available mitigation approaches, engineering and modifying cathode hosts or carriers have emerged as one of the most effective strategies, with notable progress achieved in recent years. To accelerate the practical implementation of conversion‐type cathode materials, the key challenges and their targeted solutions can be categorized into three major directions (**Figure** **21**):

1. The products formed through conversion reactions undergo significant volume expansion due to repeated size changes, which can lead to electrode cracking or pulverization. To address this, three strategies have been proposed: constructing hollow or porous structures on the cathode carrier surface, building frameworks using nanofibers, and modifying the morphology of the surface products. These approaches aim to increase the specific surface area of the cathode carrier and mitigate volume variation during cycling.
2. The formation of wide‐bandgap products during conversion reactions often results in irreversible reactions and reduced practical specific capacity. To achieve fully reversible conversion, efforts focus on lowering the free energy barrier of the reaction and inhibiting the formation of undesirable phases, thereby improving product utilization and enhancing specific capacity. Additionally, providing short diffusion pathways facilitates rapid ion and electron transport, promotes the conversion process, and enables reversible specific capacity closer to the theoretical value.
3. Side reactions commonly accompany conversion processes, mainly due to the diffusion of soluble species and reactions at the electrode–electrolyte interface. To suppress these side reactions, anchoring sites or active centers can be introduced on the cathode host surface to chemically bind with the products, thereby stabilizing the interface and promoting desired reaction pathways. Moreover, confining the conversion reaction within defined spatial structures can inhibit the formation of soluble species, effectively minimizing parasitic reactions.

Overall, conversion‐type cathode materials exhibit distinct yet complementary strengths. While S and Se cathodes deliver high performance in sodium‐ion batteries, their conversion reactions produce soluble polysulfides/polyselenides, resulting in shuttle effects and irreversibility. In comparison, transition metal oxides (TMOs) offer better reversibility and tunable conductivity, though their specific capacity remains low. Importantly, TMOs can adsorb and catalyze the conversion of polysulfides/polyselenides, thereby improving reaction kinetics and cycling stability. Their integration into Na–S/Na–Se systems helps suppress shuttle effects, enhance reversibility, and mitigate volume expansion.

**Figure 22:**
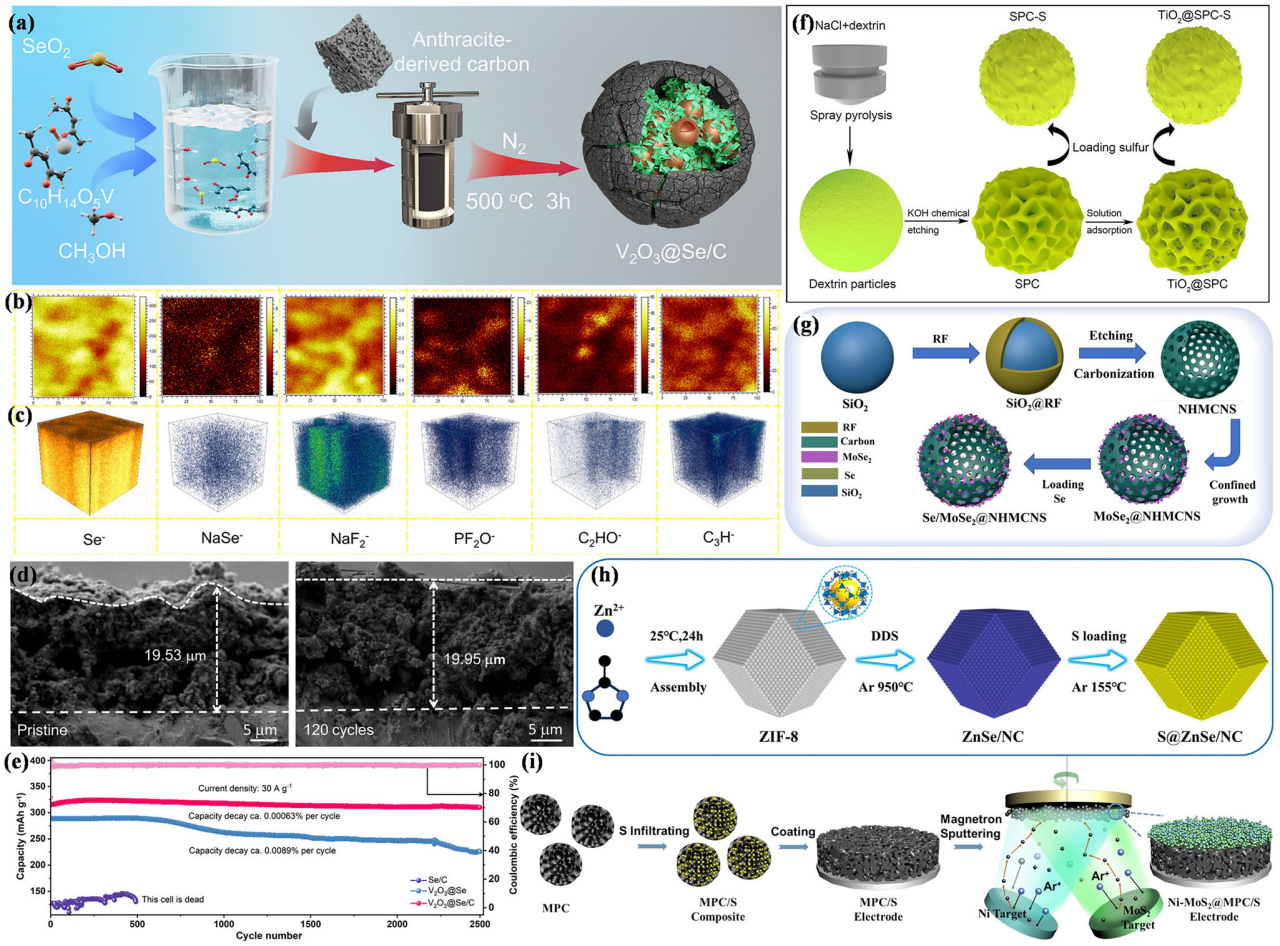
a) Schematic diagram of the synthesis process of V_2_O_3_@Se/C composite material, b) 2D variation of the TOF‐SIMS intensity corresponding to the ion fragments, and c) 3D depth profile distribution of the ion fragments corresponding to the V_2_O_3_@Se/C cathode. d) Volume expansion of V_2_O_3_@Se/C electrodes during cycling. e) The long‐term cycling curves of the Se/C, V_2_O_3_@Se, and V_2_O_3_@Se/C cathodes under a current density of 30 A g^−1^. Reproduced with permission.^[^ ^307^ ^]^ Copyright 2025, John Wiley and Sons. f) Schematic diagram of the preparation process of TiO_2_@SPC‐S. Reproduced with permission.^[^ ^308^ ^]^ Copyright 2025, Elsevier. g) Schematic diagram of the preparation of Se/MoS_e2_@NHMCNS. Reproduced with permission.^[^ ^309^ ^]^ Copyright 2025, Elsevier. h) Schematic illustration of the preparation processes of S@ZnSe/NC. Reproduced with permission.^[^ ^310^ ^]^ Copyright 2025, Elsevier. i) Schematic diagram of the preparation process for Ni‐MoS_2_@MPC/S cathode. Reproduced with permission.^[^ ^311^ ^]^ Copyright 2025, Elsevier.

Recent studies have indeed explored this direction. Wang et al.^[^ ^307^ ^]^ prepared V_2_O_3_‐coated Se nanospheres embedded in porous carbon (V_2_O_3_@Se/C), as shown in **Figure** **22**a. Unlike carbon‐only hosts, V_2_O_3_ strongly adsorbs and catalytically converts long‐chain Na_2_Se_x_, thereby enhancing selenium utilization, improving cycling stability, and mitigating volume expansion (Figure 22b–d). This composite cathode maintained a reversible capacity of over 310 mAh g^−1^ after 250 cycles, even at an ultrahigh current density of 30 A g^−1^, corresponding to an ultralow capacity decay rate of merely 0.00063% per cycle (Figure 22e). Similarly, Ma et al.^[^ ^308^ ^]^ designed TiO_2_‐modified porous carbon (TiO_2_@SPC‐S), as shown in Figure 21f, which can trap polysulfides, reduce interfacial resistance and energy barriers, facilitate charge transfer, and accommodate volume changes. Other materials, such as MoSe_2_ (Figure 22g),^[^ ^309^ ^]^ ZnSe (Figure 22h),^[^ ^310^ ^]^ CoSe_2_,^[^ ^312^ ^]^ MoS_2_(Figure 22i),^[^ ^311^ ^]^ FeS_2_, have also been employed in Na–S or Na–Se batteries. The compatibility between different conversion‐type cathodes enables their hybrid use, as demonstrated by Jiang's group in a Na–S–Se battery.^[^ ^313^ ^]^ Thus, composite cathode systems represent a promising avenue for future development.

Strategic optimization of conversion‐type cathode materials is essential for improving the electrochemical performance of sodium‐ion batteries. While the early commercialization of HT Na–S and ZEBRA batteries has demonstrated their potential in stationary energy storage, significant challenges still hinder their broad practical application. Therefore, future research should adopt a more targeted approach. We propose the following key research directions as a reference for researchers.

1. First, a deeper understanding of the reaction mechanisms in sodium metal batteries is urgently needed. Although conversion‐type cathode materials have been extensively studied in lithium systems, research in sodium batteries has largely centered on anodes. Additionally, critical aspects remain unclear, such as the detailed reaction pathways of Na–S batteries in different electrolytes, the nucleation and decomposition behavior of discharge products in Na–O_2_ batteries, and the sodium storage mechanisms in TMH systems. This mechanistic ambiguity hinders rational material design and limits progress in the field. Therefore, establishing a foundational understanding of these processes is a prerequisite for developing effective modification strategies.
2. Furthermore, modification approaches for conversion‐type cathodes should be developed with attention to performance trade‐offs. Current methods often lead to conflicting outcomes; for instance, increasing the specific surface area improves active material utilization but also promotes interfacial side reactions. Similarly, constructing porous or hollow structures mitigates volume expansion at the cost of reduced volumetric specific capacity. Optimizing these modifications to balance capacity, cycle life, and energy density represents a central challenge for commercialization.
3. Simultaneously, interfacial stability between the cathode and electrolyte must be systematically addressed. Issues such as electrode passivation in Na–O_2_ batteries and the shuttle effect in Na–S batteries significantly impair cycling performance. Moreover, interfaces involving advanced materials, such as solid electrolytes and metal halide cathodes, remain poorly understood. Developing modification strategies that enhance interfacial compatibility is critical to achieving long‐term stability and high performance.
4. More importantly, advanced computational and characterization tools should be leveraged to overcome existing technical barriers. While computational studies have begun to reveal structure–property relationships in sodiation processes, predicting intermediate phases during conversion reactions remains difficult. Experimentally, limitations in characterization techniques, such as the difficulty in detecting sodium peroxide in Na–O_2_ batteries, have led to ongoing debates regarding reaction products. Therefore, the introduction of new modeling methods and in situ or operando characterization platforms will be vital to elucidating reaction mechanisms and guiding material design, ultimately leading to significant breakthroughs in their electrochemical performance.

**Figure 23:**
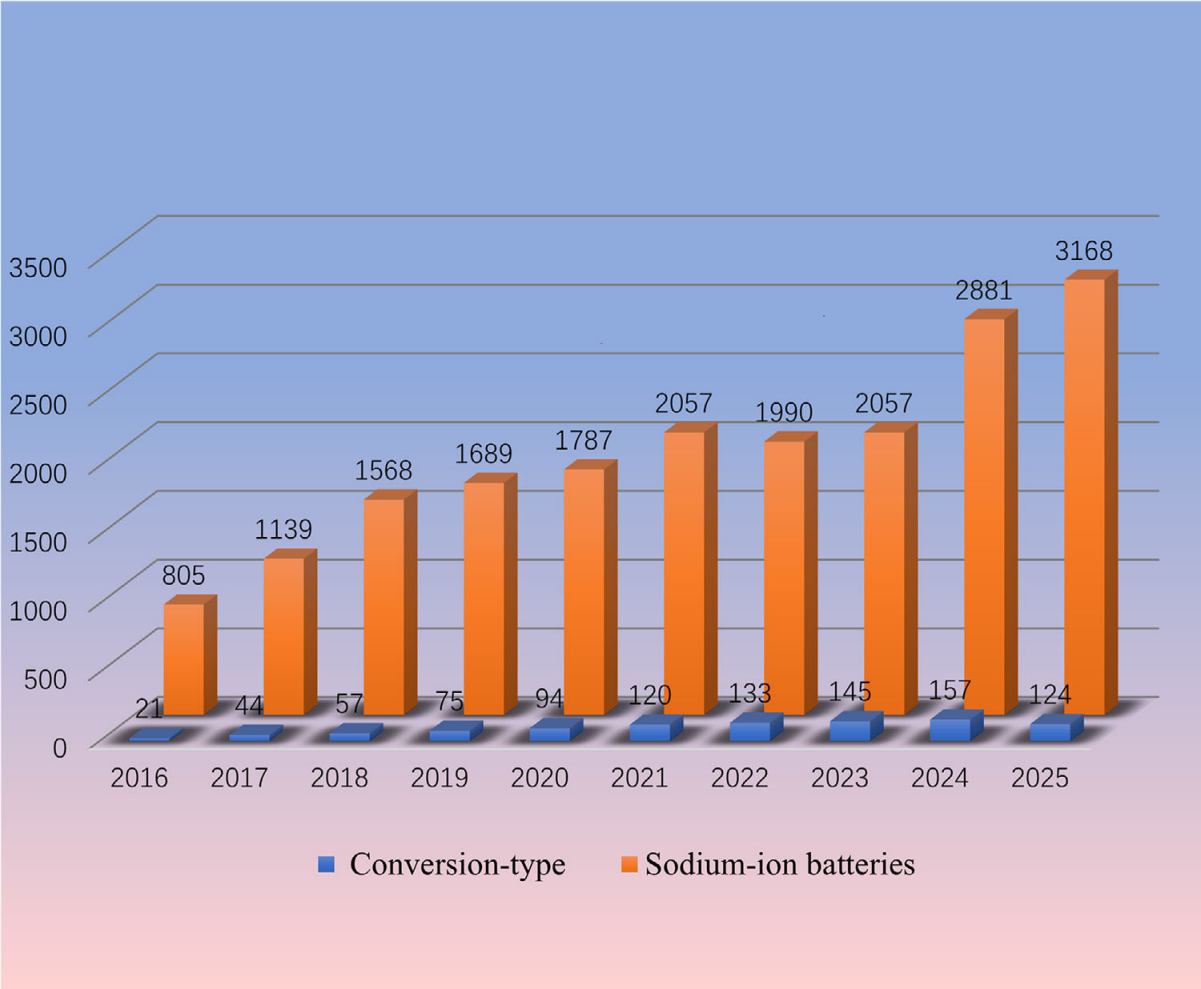
Number of publications on sodium‐ion batteries and conversion‐type over the last ten years. The orange bars represent articles related to sodium‐ion batteries, while the blue bars indicate research on conversion‐type materials.

In conclusion, conversion‐type cathode materials represent the most promising pathway for developing high‐energy‐density sodium‐ion batteries. As shown in **Figure** **23**, research interest in sodium‐ion batteries and conversion‐type materials has steadily increased over the past decade. Driven by market demands, there is an urgent need to enhance the specific capacity and economic efficiency of sodium‐based battery systems. However, neither sodium‐ion batteries nor conversion‐type cathodes can overcome commercialization hurdles until their fundamental mechanisms and intrinsic limitations are thoroughly elucidated. Therefore, we hope this review provides a systematic overview of recent advances in conversion‐type cathode materials. It is also hoped to stimulate further innovative research and accelerate the technological breakthroughs needed for sodium batteries to achieve success in future energy applications.

## Conflict of Interest

The authors declare no conflict of interest.
